# Lysophagy protects against ANXA11 amyloid fibril toxicity and propagation in FTLD

**DOI:** 10.1186/s40035-026-00561-5

**Published:** 2026-06-28

**Authors:** Honglin Zheng, Haiyang Luo, Yongting Lu, Yapei Yuan, Na Zhang, Suying Duan, Zongping Xia, Yuming Xu

**Affiliations:** 1 Department of Neurology, The First Affiliated Hospital of Zhengzhou University, Zhengzhou University, Jian-She East Road, Zhengzhou, 450000 Henan China; 2 NHC Key Laboratory of Prevention and Treatment of Cerebrovascular Disease, Zhengzhou, China; 3 Henan Key Laboratory of Cerebrovascular Diseases, Zhengzhou University, Zhengzhou, China; 4 Department of Nephrology, The First Affiliated Hospital of Zhengzhou University, Zhengzhou University, Zhengzhou, China; 5 Department of Pathology, The Second Affiliated Hospital of Zhengzhou University, Zhengzhou University, Zhengzhou, China; 6 Clinical Systems Biology Laboratories, Translational Medicine Center, The First Affiliated Hospital of Zhengzhou University, Zhengzhou, China; 7 Tianjian Laboratory of Advanced Biomedical Sciences, School of Life Sciences, Zhengzhou University, Zhengzhou, China

**Keywords:** Annexin A11, Frontotemporal lobar degeneration, Lysophagy, Lysosomal membrane permeabilization, Prion-like propagation, Cerebral organoids

## Abstract

**Background:**

Accumulation of Annexin A11 (ANXA11) aggregates is a distinct pathological hallmark of amyotrophic lateral sclerosis (ALS) and frontotemporal lobar degeneration (FTLD). While genetic studies have linked *ANXA11* mutations (e.g., D40G) to disease, the precise molecular events converting aggregation into neurotoxicity and intercellular propagation remain elusive. We hypothesize that lysosomal integrity serves as a critical checkpoint in ANXA11 proteinopathy and that its failure drives disease progression.

**Methods:**

To model the human pathology of ANXA11, we generated pre-formed fibrils (PFFs) of wild-type and FTLD/ALS-linked D40G mutant ANXA11. Human iPSC-derived neurons, 3D cerebral organoids, and bulk RNA-sequencing were employed to investigate neurotoxicity. High-resolution imaging, lentiviral knockdown, and biochemical assays were performed to delineate the lysosomal damage response and the subsequent "prion-like" spreading of aggregates.

**Results:**

The internalized ANXA11 fibrils accumulated in lysosomes, triggering lysosomal membrane permeabilization (LMP). The D40G mutation exacerbated this toxicity, leading to severe LMP, mitochondrial depolarization, and specific transcriptional downregulation of the dynactin subunit ACTR10. Mechanistically, we identified a protective signaling axis involving p38 MAPK, MK2, and HSP27 that senses ANXA11-induced lysosomal damage and initiates lysophagy. Notably, in human cerebral organoids, failure of this lysophagic clearance facilitated the cytoplasmic escape of ANXA11, thereby accelerating its seeding activity and propagation to neighboring cells. Pharmacological or genetic modulation of this pathway significantly altered neuronal survival.

**Conclusions:**

Our study established lysosomal rupture as a primary driver of ANXA11-associated neurodegeneration and validated the p38/MK2/HSP27 axis as a crucial defense mechanism in human neural tissue. These findings provide a novel mechanistic link between lysosomal quality control and ANXA11 propagation, highlighting that enhancing lysophagic flux represents a promising translational strategy to halt the progression of FTLD and ALS.

**Graphical abstract:**

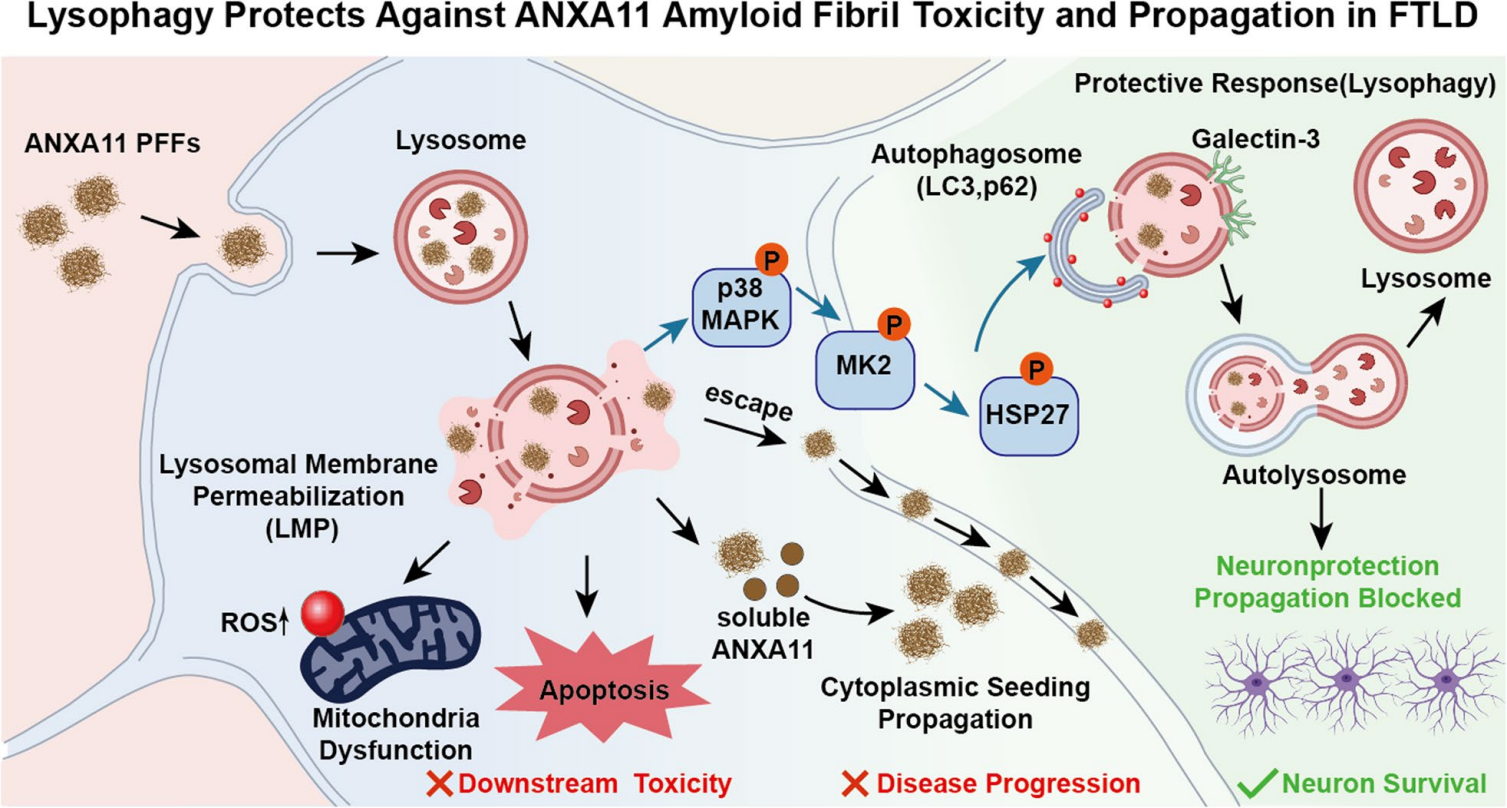

**Supplementary Information:**

The online version contains supplementary material available at 10.1186/s40035-026-00561-5.

## Background

Frontotemporal lobar degeneration (FTLD) is a neurodegenerative disorder characterized by progressive degeneration of the frontal and temporal lobes, leading to significant cognitive and behavioral impairments [1, 2]. A pathological hallmark of these conditions is the accumulation of misfolded proteins into amyloid-like fibrils. While TDP-43 is the predominant pathological protein, mutations in the Annexin A11 (*ANXA11*) gene have recently emerged as a critical genetic cause of multisystem proteinopathy [3], including FTLD [4, 5]. Under physiological conditions, ANXA11 functions as a molecular tether, linking RNA granules to lysosomes for long-distance neuronal transport [6]. However, pathogenic mutations, particularly those within the low-complexity domain (LCD), accelerate the phase transition of ANXA11 from liquid droplets to irreversible, amyloid-like fibrils [7–9]. Recent cryo-electron microscopy (cryo-EM) studies have definitively identified ANXA11 homomeric and heteromeric amyloid filaments in the brains of FTLD-TDP type C patients [4], establishing ANXA11 aggregation as a distinct and potent driver of neurodegeneration.

A prevailing hypothesis in neurodegeneration is the "prion-like" propagation of misfolded proteins, where pathological seeds are released from affected neurons and internalized by neighboring cells [10, 11]. Upon internalization, these aggregates typically converge on the endolysosomal pathway. Lysosomes are essential organelles responsible for degrading cellular waste and damaged proteins. However, the accumulation of proteolysis-resistant amyloid fibrils can overwhelm the lysosomal capacity, posing a severe threat to cellular integrity.

When lysosomes become overloaded with rigid fibrils, they are susceptible to lysosomal membrane permeabilization (LMP) [12, 13]. LMP is a catastrophic cellular event; it results in the leakage of luminal protons and proteolytic enzymes, such as cathepsins, into the cytosol. This leakage not only triggers downstream cascades leading to mitochondrial dysfunction and cell death, but may also paradoxically facilitate the escape of internalized seeds into the cytoplasm, promoting the templated misfolding of endogenous proteins [14, 15]. Thus, maintaining lysosomal integrity is a pivotal checkpoint in preventing both neurotoxicity and the propagation of proteinopathy.

To mitigate the damage caused by lysosomal rupture, cells deploy two key quality control mechanisms: the endosomal sorting complex required for transport (ESCRT) machinery for membrane repair [16], and lysophagy, a selective form of autophagy that sequesters and degrades irreversibly damaged lysosomes [17, 18]. While these mechanisms are known to protect against other protein aggregates, it remains unclear whether and how neurons mobilize lysophagy to defend against ANXA11 amyloid fibrils. Furthermore, the specific upstream signaling pathways that sense ANXA11-induced lysosomal stress and initiate this protective response are yet to be defined.

In the present study, we investigated how ANXA11 preformed fibrils (PFFs) impact lysosomal integrity and protein aggregation in human neuroblastoma cells, induced pluripotent stem cell (iPSC)-derived neurons, and cerebral organoids. We aimed to elucidate the cellular mechanisms that protect against ANXA11-induced lysosomal stress and explore the effects of FTLD/ALS-associated mutations on the propagation of ANXA11 aggregates.

## Materials and methods

### Expression and purification of ANXA11

The full-length human *ANXA11* (WT/D40G) (amino acids 1–505) was cloned into the pCold TF vector (Takara Bio, #3365, Beijing, China) containing an N-terminal His-TF tag followed by an HRV 3C protease cleavage site. The recombinant plasmid was transformed into *E.coli* BL21 (DE3) competent cells. Transformed bacteria were cultured in LB medium at 37°C until the OD600 reached 0.6–0.8, followed by induction with 0.5 mM isopropyl β-D-1-thiogalactopyranoside (IPTG), and then cultured at 16 °C for 16–18 h with shaking. Cells were harvested by centrifugation at 4000 rpm for 30 min at 4 °C. The pellet was resuspended in lysis buffer (50 mM phosphate buffer, 300 mM NaCl, 10 mM imidazole, pH 8.0) and lysed by high-pressure homogenization. The lysate was centrifuged at 12,000 rpm for 40 min at 4 °C, followed by filtration through a 0.22-μm membrane (Millipore, Burlington, MA). The clarified lysate was applied to a Ni–NTA column pre-equilibrated with lysis buffer. After washing, the bound proteins were eluted with a linear 100–500 mM imidazole gradient. The eluted fractions containing ANXA11 were pooled and further purified by anion-exchange chromatography (HiTrap Q HP column, GE Healthcare) with a NaCl gradient (50 mM–1 M) in 20 mM Tris–HCl (pH 8.0). Finally, the purified protein was dialyzed against storage buffer (20 mM Tris–HCl, 150 mM NaCl, pH 7.4). Protein purity was assessed by SDS-PAGE, and concentration was determined by the Bradford assay.

### Preparation of ANXA11 PFFs

Specifically, 50 μM of recombinant ANXA11 protein was incubated in 20 mM Tris–HCl (pH 7.4), 150 mM NaCl buffer, then agitated at 1000 rpm at 37 °C for 72 h. Then, the PFFs were sonicated by an ultrasonic cell disruptor at 10% of its peak amplitude (Scientz-IID, Ningbo, China) at the 3 s on–3 s off processor duty cycle for 2 min. Sequentially, the prepared ANXA11 PFFs were immediately frozen and stored in a − 80 °C freezer. The morphology and amyloid nature of the PFFs were characterized by Congo red and Thioflavin S staining, as well as transmission electron microscopy (TEM).

### Congo red and Thioflavin S staining

A 2 µL aliquot of the sample was air-dried on glass slides. For Congo red staining, slides were stained with 0.5% (*w*/*v*) Congo red solution (Servicebio, #G1056, Wuhan, China) for 2 min, and rinsed with 100% ethanol. Slides were mounted with neutral gum, and images were captured under natural and polarized light using a polarized light microscope (Carl Zeiss, Axioscope 5 Pol, Jena, Germany). For Thioflavin-S staining, slides were incubated in 0.2% (*w*/*v*) Thioflavin-S solution (Servicebio, GDP1028) for 2 min, washed with ethanol, dehydrated in xylene, and mounted with an anti-fade agent. Images were captured by polarized or fluorescence microscopy (Nikon, Eclipse Ti2, Tokyo, Japan) under appropriate channels.

### TEM

The ANXA11 PFFs underwent two-fold dilution, adsorbed to glow-discharged 400-mesh carbon-coated copper grids for 2 min, quickly washed twice with Tris–HCl (50 mM, pH 7.4), and floated upon two drops of 0.75% uranyl formatted for 30 s. The grids were allowed to dry before imaging under HT7800 TEM operating at 80 kV. The images were captured and digitized with an ER-80 CCD (8 mega pixels).

The cells were cultured on a Cell Desk polystyrene cover slip (Sumitomo Bakelite Co., Ltd., Tokyo, Japan) at 25,000 cells per well, treated with 2 µM PFFs, fixed with 2% formaldehyde and 2.5% glutaraldehyde in 0.1 M sodium-phosphate buffer (pH 7.4), and washed 3 × 5 min in the same buffer. The cells were post-fixed for 1 h with 1% osmium tetroxide and 1% potassium ferrocyanide in 0.1 M sodium-phosphate buffer (pH 7.4), dehydrated in a graded series of ethanol, and embedded in Epon812 (TAAB Co., Ltd., T026, Aldermaston, UK). Ultra-thin (80 nm) sections were stained with saturated uranyl acetate and lead citrate solution. Electron micrographs were obtained with a JEM-1400plus transmission electron microscope (JEOL, Tokyo, Japan).

### Cell cultures

Human neuroblastoma SH-SY5Y cells obtained from Shanghai Zhong Qiao Xin Zhou Biotechnology Co., Ltd. (ZQ0050) and HEK 239 T cells obtained from Procell Life Science & Technology (CL-0005) were cultured in Dulbecco’s modified Eagle’s medium (DMEM)/F12 medium (Gibco, 11320033, Grand Island, NY) supplemented with 10% (*v*/*v*) fetal bovine serum (FBS, Cell-Box, AUS-01S-02, Hong Kong, China), and penicillin–streptomycin-glutamine (Gibco, 10378016). The cells were maintained at 37 °C in a humidified incubator with 5% CO₂. The cells were routinely maintained within 20 passages to minimize phenotypic drift associated with long-term culture. Mycoplasma contamination was monitored on a monthly basis using the MycoAlert™ Mycoplasma Detection Kit (LT07-318, Lonza, Switzerland), and all tests were consistently negative.

The cells were seeded to reach 60%–80% confluency and transfected with plasmids using Lipofectamine 3000 (Thermo Fisher Scientific, L3000015, Waltham, MA) according to the manufacturer’s protocol. In each well of the 24-well plate, 0.5 μg DNA and 1 μL P3000 reagent were diluted in 25 μL Opti-MEM and mixed with 1.5 μL Lipofectamine 3000 diluted in 25 μL Opti-MEM. After a 10–50 min incubation at room temperature, the complexes were added to the cells in antibiotic-free medium. The medium was replaced with fresh complete medium 4–6 h later, and cells were analyzed 24–72 h post-transfection.

### iPSC-derived neuron culture

Human iPSCs (H9 line, WiCell Research Institute, Madison, WI) were maintained under feeder-free conditions on Matrigel-coated plates in mTeSR1 medium (StemCell Technologies, 85850, Vancouver, Canada). The identity of the iPSC line was verified by short tandem repeat (STR) profiling, karyotype analysis, and immunocytochemical staining for pluripotency markers (OCT4, SOX2, NANOG, and TRA-1–60). Mycoplasma contamination was routinely monitored and excluded. iPSC-derived neurons were generated according to the manufacturer’s instructions for the BrainPhys neuronal medium (Stemcell, 05833). The iPSC-derived neural progenitor cells were obtained following the manufacturer’s instructions for the iPSC neural induction medium (Gibco, A1647801). The iPSCs were passaged at an appropriate density to the Matrigel-coated plates with 10 μM Y27632 in an iPSC culture medium. The medium was changed to a complete iPSC neural induction medium on day 1 and the morphology of cell colonies was confirmed to be uniform on day 2. The complete neural induction medium was changed on days 2, 4 and 6. Neural progenitor cells (P0) were harvested and expanded on day 7. Neural progenitor cells were expanded for three passages. Differentiation into neurons was initiated at the third passage. The neuronal differentiation medium consisted of Neurobasal (Gibco, 21103049), 2% B27 (Gibco, 17504044) and 1% GlutaMAX (Gibco, 35050061), and subsequent experiments began on the seventh day of differentiation.

### Western blot

3D cerebral organoids (3DCOs) at 40 days or cultured cells were lysed in RIPA buffer (Servicebio, G2002) containing phosphatase inhibitors and protease inhibitors (P1005, Beyotime). Proteins were extracted by centrifugation at 13,000 × *g* for 10 min at 4 °C. Protein concentration was determined using the Pierce BCA protein assay kit as per the manufacturer’s instructions (ThermoFisher, A65453). Then the protein mixture was heated at 95 °C in 5 × sample buffer for 10 min. A total amount of 10 µg total protein was separated by SDS-PAGE, and transferred to a PVDF membrane (Millipore). The membranes were incubated with primary antibody (Table S1) at 4 °C overnight. A ChemiDoc imaging system (Bio-Rad, Hercules, CA) was used for immunoblot visualization with ECL (ABclonal, RM00021, Wuhan, China), and protein bands were quantified with ImageJ (version 1.53t, Bethesda, MD).

### Dot blot

For dot blot analysis, 5 µg of total protein from each sample was spotted directly onto a nitrocellulose membrane (Millipore) and air-dried for 10 min at room temperature. The nitrocellulose membrane was blocked with non-fat dry milk (5% in TBST) for 1 h at room temperature. The information of primary antibodies used in this study is provided in Table S1.

After washing the primary antibodies with TBST, the membrane was incubated with HRP-conjugated Goat Anti-Rabbit IgG (Proteintech, SA00001-2, 1:5000 dilution in TBST) for 1 h at room temperature. Signals were detected using an ECL reagent (ABclonal) and visualized with a ChemiDoc imaging system (Bio-Rad).

### Fluorescent labelling of ANXA11 aggregates

ANXA11 aggregates (100 µg) were fluorescently labeled with either Alexa Fluor 488 (Abcam, ab236553, Cambridge, UK) or Alexa Fluor 647 (Abcam, ab269823) at a protein-to-dye ratio of 1:1 (*w*/*w*), according to the manufacturer’s protocol. The labeling reaction was carried out by incubating the samples for 20 min at room temperature without agitation, as gentle static incubation was sufficient for efficient coupling. The reaction was terminated by adding 20 µL quencher and incubation for 5 min.

### Cell treatment with ANXA11 fibrils

ANXA11 PFFs were added to cultured cells at a final concentration of 5 μg/mL using Lipofectamine 3000 (Thermo Fisher Scientific, L3000015) as a protein delivery reagent, according to the manufacturer’s instructions. Briefly, fibrils were mixed with Lipofectamine 3000 in Opti-MEM medium for 15 min at room temperature to allow complex formation, and the complexes were then applied to cells at the indicated final concentrations. This approach ensured efficient delivery of fibrils into the cytoplasm and avoided potential artifacts from spontaneous internalization.

### siRNA knockdown

For knockdown of RB1 Inducible Coiled-Coil 1 (*RB1CC1*), *ALIX*, and *TSG101*, both siRNA and shRNA approaches were employed. Initially, three distinct siRNAs targeting each gene were tested for knockdown efficiency. A most effective siRNA was selected for each target, and the corresponding shRNA was subsequently used for stable knockdown. The following shRNA sequences were used: *RB1CC1* shRNA: 5′-GCAAAGAAATTAGGGAATCTT-3′; *ALIX* shRNA: 5′-CCAGAACAAATGCAGTGATAT-3′; *TSG101* shRNA: 5′- GCCTTATAGAGGTAATACATA-3′. For transient knockdown, siRNAs (10 pmol) were transfected into cells using Lipofectamine RNAiMAX (Invitrogen, 13778075, Carlsbad, CA) according to the manufacturer’s instructions.

For stable knockdown of *RB1CC1*, *ALIX*, and *TSG101*, shRNA sequences were cloned into the pLKO.1 lentiviral vector (Addgene, #8453, Watertown, MA). Lentiviral particles were produced by transfecting HEK293T cells with the shRNA-containing pLKO.1 vector, along with packaging plasmids (psPAX2 and pMD2.G) using Lipofectamine 3000 according to the manufacturer’s protocol. Forty-eight hours post-transfection, the supernatant containing lentivirus was collected, filtered, and used to infect SH-SY5Y cells. Infected cells were selected with puromycin (2 μg/mL) for 7 days to establish stable knockdown cell lines. After puromycin selection, stable cell lines were replated into 24-well plates. PFFs and Lipofectamine 3000 were added to the culture medium.

### iPSC-derived cerebral organoid culture

3DCOs were generated from iPSC lines according to the manufacturer’s instructions of the STEMdiff Cerebral Organoid Kit (Stemcell, #08570). Briefly, on day 0, any differentiated cells in the iPSC culture were removed. iPSC colonies were dissociated into single-cell suspensions with Accutase (Stemcell, #07902). In total, 9000 cells were then plated into each well of a 96-well round-bottom ultra-low attachment plate (Corning, #7007) in the embryoid body (EB) formation medium supplemented with 10 μM Y-27632 (Merck, # Y0503). Then 100 μL of EB formation medium was added on days 2 and 4. EBs were moved to 24-well ultra-low attachment plates (Corning, #3473) and cultured in the induction medium for another 2 days. EBs were then embedded into 15 μL of Matrigel and were cultured in the expansion medium for 4 days in 6-well ultra-low adherent plates (Corning, #3471) for organoid formation. In the maturation stage, the medium was changed to a maturation medium and plates were moved onto an orbital shaker for further maturation. The maturation medium was replaced every 3 days. 3DCOs were collected at 40 days in vitro (DIV) for immunostaining and immunoblotting.

### Live-cell imaging and kymograph analysis

To evaluate axonal transport, human iPSC-derived neurons were cultured on glass-bottom dishes. Lysosomes were tracked using 50 nM LysoTracker™ Red DND-99 (30-min incubation). For RNA granules, neurons expressing fluorescently tagged ANXA11 and G3BP1 were utilized. Following PFFs treatments (5 µg/mL), time-lapse imaging was performed in Live Cell Imaging Solution using a Zeiss LSM 980 confocal microscope equipped with an environmental chamber (37 °C, 5% CO₂). Images were acquired continuously for 30 s.

Kymographs were generated from time-lapse stacks using the Multi Kymograph plugin in ImageJ. The average velocity (µm/s) of motile particles was calculated from the slopes of diagonal trajectories. Particles with a total displacement of < 2 µm over the 30 s recording were defined as stationary. The fraction of stationary events was calculated as the percentage of stationary trajectories relative to total trajectories per neurite. At least 10 independent neurites were analyzed per condition across biological replicates.

### Immunostaining

For cell culture, SH-SY5Y cells and iPSC-derived neurons were seeded onto sterilized glass coverslips placed in 24-well plates and treated with AF488- or AF647-labeled ANXA11 PFFs at a concentration of 5 μg/mL for 24 h. After treatment, cells were fixed with 4% paraformaldehyde at room temperature for 15 min. The fixed cells were then washed three times with PBS and stored in PBS until further processing.

At 40 DIV, 3DCOs were fixed in 4% paraformaldehyde for 30 min and washed with phosphate-buffered saline (PBS), dehydrated with 30% sucrose at 4 °C, then embedded with an optical cutting temperature compound (VWR, Radnor, PA) and frozen in liquid nitrogen. Frozen tissues were sectioned at 15 μm using a cryostat and collected on ultra-frosted glass microscope slides. Sections were stored at − 20 °C until use.

For immunostaining, both cell culture and tissue sections were permeabilized with 0.3% Triton X-100 in PBS and blocked with 5% bovine serum albumin in PBS. Sections were incubated with primary antibodies diluted in the blocking buffer overnight at 4 °C, washed three times with PBS, and incubated with fluorescently conjugated secondary antibodies (Alexa Fluor 488 and 594 conjugates, ab150077 and ab150116, Abcam, 1:500) for 2 h at room temperature and washed three times with PBS before mounting with glass coverslips. Nuclei were stained with DAPI (4′,6-diamidino-2-phenylindole). Confocal image stacks covering the entire cell volume were acquired using a Zeiss LSM 980 confocal microscope. Z-stack images were collected where appropriate, and all imaging parameters were kept constant across groups for quantitative comparison.

For the quantification of ANXA11-GFP inclusions, cells were operationally scored as 'positive for inclusions' only if they exhibited one or more distinct, intensely bright, and irregularly shaped focal aggregates characteristic of solid-like assemblies. This criterion strictly distinguished pathological aggregates from diffuse cytosolic expression or the dynamic, spherical liquid-like droplets often observed under baseline conditions. For each condition, a minimum of 150 cells from randomly selected fields of view were manually counted by an investigator blinded to the experimental treatments across at least three independent biological replicates.

### Flow cytometry (FCM)

For flow cytometric analysis, cells treated with ANXA11 PFFs and CA-074Me (MedChemExpress, HY-100201, Monmouth Junction, NJ) were trypsinized and scraped from the culture plate. The cell suspension was centrifuged at 200 × *g* for 5 min at 4 °C and resuspended in 1 mL of FCM buffer, consisting of 2 mM EDTA, 0.5% (*w*/*v*) bovine serum albumin, and 0.1% (*w*/*v*) sodium azide in PBS (pH 7.4). After an additional round of centrifugation, the pellet was resuspended in 200 μL of FCM buffer containing 2% (*w*/*v*) paraformaldehyde (Carl Roth, Karlsruhe, Germany) to fix the cells for 15 min. After resuspension in 400 μL of FCM buffer, the cells were transferred to an FCM tube (Sarstedt, Nümbrecht, Germany) for analysis using a BD FACSVerse™ flow cytometer (BD Biosciences, San Jose, CA) with the following excitation/emission settings: 488/511–543 nm (for ANXA11-AF488). For each sample, 14,000–20,000 events were recorded. Data analysis was performed using FlowJo software (FlowJo, LLC, Ashland, OR).

### Apoptosis detection by FCM

Apoptosis was assessed using an Annexin V-PE/PI staining kit (BioLegend, 640914, San Diego, CA). Cells were harvested by trypsinization, washed with PBS, and resuspended in binding buffer. The cells were then stained with Annexin V-APC and Propidium Iodide (PI) according to the manufacturer's protocol. Briefly, 5 µL of Annexin V-PE and 5 µL of PI were added to the cells, followed by incubation for 15 min at room temperature in the dark. The samples were analyzed immediately using a BD LSRFortessa flow cytometer (BD Biosciences). The FCM data were analyzed using FlowJo software.

The results were plotted on a 2D dot plot where the x-axis represents Annexin V-PE fluorescence intensity (Comp-APC-A::Annexin V), and the y-axis represents PI fluorescence intensity (Comp-PE-A::PI). Quadrant analysis was used to categorize the populations into early apoptotic (Annexin V-positive/PI-negative), late apoptotic (Annexin V-positive/PI-positive), and viable cells (Annexin V-negative/PI-negative).

### Lysotracker and GFP-mCherry-LC3B dual-colour staining

The lysotracker staining was conducted according to the LysoTracker Green user manual (ThermoFisher, A66439). The GFP-mCherry-LC3B dual-colour staining was conducted using the pCMV-mCherry-GFP-LC3B plasmid (Beyotime, D2816) according to the manufacturer’s instructions.

### ROS detection

Cells were treated with 2.5 μmol/L 2,7-dichlorodihydrofluorescein (DCFH-DA, D6883-50MG, Sigma-Aldrich) and heated at 37 °C for 1 h. Then, FCM was used to detect oxidation of the intracellular fluorophores at an excitation wavelength of 488 nm and an emission wavelength of 535 nm. The results are expressed as the mean fluorescence intensity.

### JC-1 staining for mitochondrial membrane potential analysis

Mitochondrial membrane potential (Δψm) was assessed using JC-1 (Beyotime, #C2006) according to the manufacturer’s instructions. Briefly, cells were cultured to approximately 70% confluence in 6-well plates. After treatment with the experimental compounds, the cells were washed twice with PBS, followed by incubation with 2 μM JC-1 dye in PBS for 20 min at 37 °C. After incubation, the cells were washed twice with PBS to remove excess dye and analyzed using a fluorescence microscope (excitation at 490 nm and emission at 530 nm for the monomeric form and excitation at 525 nm and emission at 590 nm for the aggregated form). The ratio of red (aggregated JC-1) to green (monomeric JC-1) fluorescence intensity was calculated to assess mitochondrial membrane potential, with higher red-to-green fluorescence ratio indicating higher Δψm.

### Lysosomal labeling and live-cell imaging

To assess lysosomal acidification and integrity, SH-SY5Y seeded in glass-bottom dishes (MatTek, P35G-1.5–14-C, Ashland, MA) were stained with the acidotropic probe LysoTracker™ Red DND-99 (Thermo Fisher Scientific, A66439). Ideally, cells were incubated with pre-warmed culture medium containing 50–75 nM LysoTracker Red for 30–60 min at 37 °C in a humidified 5% CO₂ incubator. During the final 10 min of incubation, nuclei were counterstained with Hoechst 33342 (1 µg/mL; Thermo Fisher Scientific). Following incubation, the medium was replaced with phenol red-free Live Cell Imaging Solution (Invitrogen) to reduce background autofluorescence. Live-cell imaging was immediately performed using a confocal laser scanning microscope (e.g., Leica SP8 or Zeiss LSM 880) equipped with a stage-top incubator maintained at 37 °C and 5% CO₂. LysoTracker Red signals were excited at 577 nm, and emission was collected at 590 nm. Image analysis was performed using ImageJ/Fiji software, quantifying the mean fluorescence intensity or the number of LysoTracker-positive puncta per cell.

The CELLImage Mini automated live-cell imaging system (Chongqing Lianqing Ruiqi Co., Ltd., Chongqing, China) was surface-sterilized with alcohol and placed inside an SH-SY5Y cell incubator. SH-SY5Y cells were seeded into cell culture vessels at a density of 2×10^4^ cells/cm^2^. Following ANXA11 PFFs treatment, the vessels were transferred to the observation area of the imaging system. A dynamic monitoring protocol was configured to capture cell images at an interval of 4 h per cycle, for a total of 6 cycles.

### Bulk RNA sequencing

RNA was extracted with the TRIzol reagent (Invitrogen). The quantity and quality of RNA samples for bulk RNA sequencing were determined by Agilent 2100 Bioanalyzer (Agilent, Santa Clara, CA). mRNA samples were sequenced using PromethION (Oxford Nanopore Technologies, Oxford, UK). Reads were mapped to the human reference genome hg38. Raw reads were filtered with an average read quality score ≥ 7 and read length ≥ 500 bp. The expression level was estimated by reads per gene/transcript per 10,000 reads mapped. Differential expressions was analyzed using the DESeq2R package. DESeq2 provides statistical routines for determining the differential expression in digital gene expression data using a model based on the negative binomial distribution. The resulting *P* values were adjusted using Benjamini and Hochberg’s approach for controlling the false discovery rate. Genes with a* P*-value < 0.05 and foldchange ≥ 1.2 found by DESeq2 were assigned as differentially expressed. The statistical enrichment of differentially expressed genes in Kyoto Encyclopedia of Genes and Genomes (KEGG) pathways was tested using KOBAS.24 (Beijing, China).

### Colocalization analysis by intensity profile

Colocalization analysis was performed by evaluating the fluorescence intensity profiles along a defined line across regions of interest. Confocal images were acquired using a Zeiss LSM 980 confocal microscope. Fluorescence intensities of different channels were measured along a 15-μm distance in the image using the Zen 3.0 software (Zeiss). Fluorescence intensity was plotted against distance to visualize the relative distribution and potential overlap of these signals. Peaks in the intensity profiles correspond to regions of high signal overlap, indicating colocalization between the different channels. Graphs were generated using GraphPad Prism (GraphPad Software, La Jolla, CA).

### Multiplex immunohistochemical staining

The collected tissue sections were dewaxed with conventional xylene and hydrated through a graded alcohol series. Multiplex immunohistochemistry was performed using a seven-color staining kit (AFIHC027, AiFang Biologcal, Changsha, China) according to the manufacturer’s instructions. Sections were subjected to antigen retrieval with microwave heating, incubated with 3% hydrogen peroxide for 15 min at room temperature, and blocked with 10% goat serum for 15 min. The following primary antibodies were applied sequentially overnight at 4 °C: NeuN (AFRM0088, AiFang), MAP2 (17490-1-AP, Proteintech), PAX6 (AFRM0226, AiFang), TBR1 (AFRM0110, AiFang), and TUJ1 (AFRM0073, AiFang). Between each two rounds, antibodies were removed by microwave treatment, and sections were incubated with polymer-HRP anti-mouse/rabbit universal secondary antibody IgG (AFIHC001, AiFang Biologcal) and the TYR fluorescent dye for 8 min, followed by three washes with PBST. After completing all staining rounds, sections were incubated with DAPI for nuclear staining at room temperature for 10 min, washed three times with PBST, mounted with anti-fade mounting medium, and imaged using an eight-channel fluorescence digital slide scanner (AF-KL-20-8, AiFang Biologcal).

### Statistical analysis

Statistical analyses were performed using GraphPad Prism 8 software. All data were tested for normality and variance. Comparisons between two groups were performed with Student’s *t*-test. Comparisons among three or more groups were performed with one-way analysis of variance followed by Tukey’s post-hoc test. Data are presented as means ± SEM. The sample size for each statistical analysis is indicated in the corresponding figure legends. Statistical significance was set at *P* < 0.05.

## Results

### ANXA11 aggregates are internalized into lysosomes in neurons

**Fig. 1:**
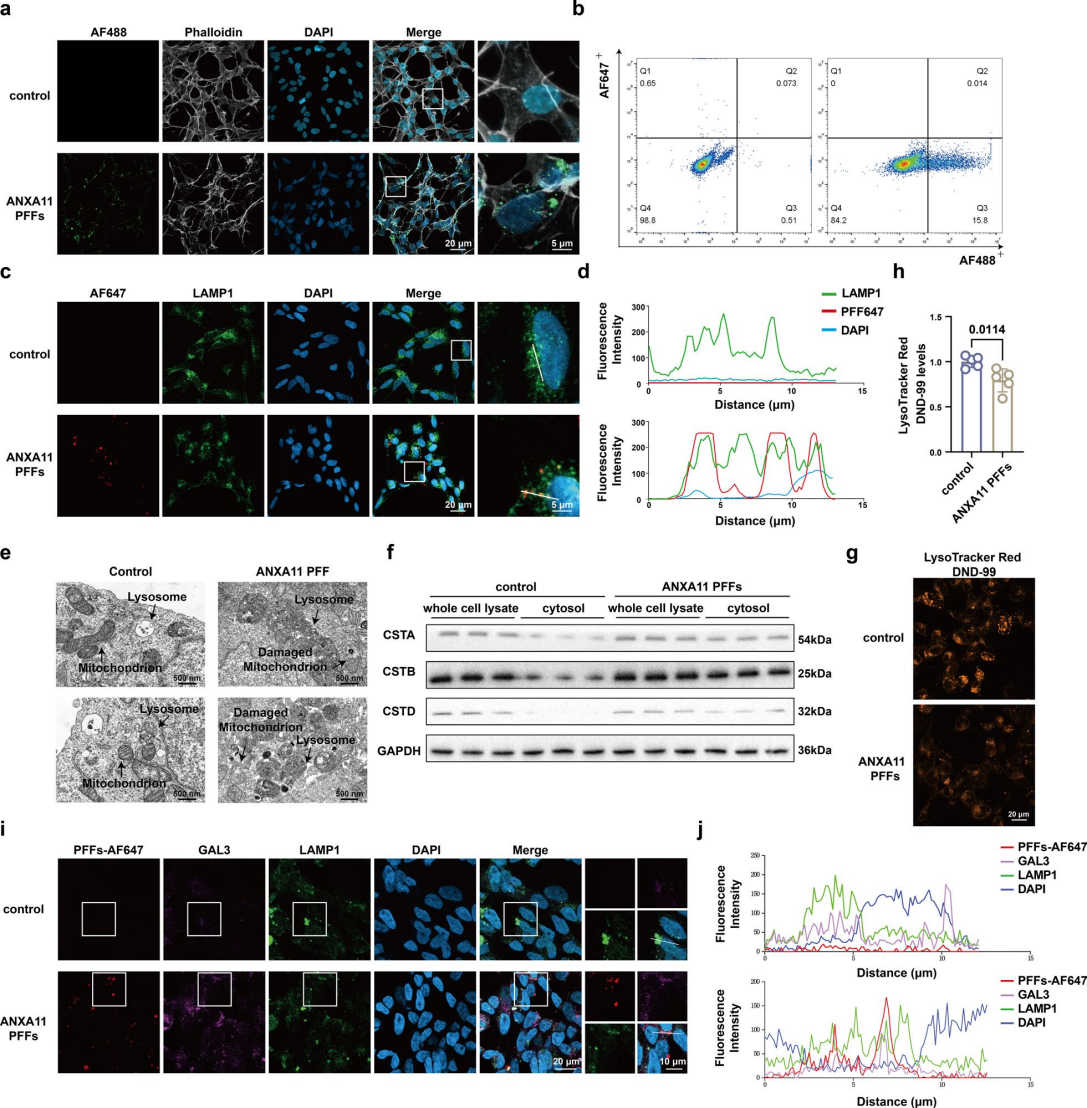
Internalized ANXA11 fibrils accumulate in lysosomes and trigger lysosomal membrane permeabilization (LMP) in neurons.** a** Representative confocal microscopy images of SH-SY5Y cells treated with Alexa Fluor 488-labeled ANXA11 preformed fibrils (PFFs-AF488, green) for 24 h. Cytoskeleton was stained with phalloidin (white) and nuclei with DAPI (blue). The images show intracellular localization of ANXA11 aggregates. **b** Flow cytometry analysis quantifying the uptake of ANXA11 PFFs by SH-SY5Y cells after 24 h treatment. **c** Confocal images showing the subcellular localization of internalized ANXA11 PFFs-AF647 (red) and the lysosomal marker LAMP1 (green). **d** Fluorescence intensity profile analysis along the white line drawn in (**c**), demonstrating the colocalization of ANXA11 PFFs with lysosomes. **e** Transmission electron microscopy images of SH-SY5Y cells treated with ANXA11 PFFs. Arrows indicate electron-dense aggregates within lysosomes and damaged mitochondria with disrupted cristae. **f** Western blot analysis of cathepsins (CSTA, CTSB, and CTSD) in cytosolic versus whole-cell lysate fractions. The presence of mature cathepsins in the cytosolic fraction upon PFFs treatment indicates lysosomal leakage. GAPDH was used as a loading control. **g** Representative images of Lysotracker Red staining in SH-SY5Y cells treated with or without ANXA11 PFFs, assessing lysosomal acidity and integrity. **h** Quantification of Lysotracker Red fluorescence intensity shown in (**g**). **i** Immunofluorescence images showing the recruitment of galectin-3 (GAL3, purple), a marker of lysosomal rupture, to LAMP1-positive lysosomes (green) containing ANXA11 PFFs-AF647 (red). **j** Fluorescence intensity profile analysis along the line in (**i**), showing the colocalization of GAL3 with ruptured lysosomes containing ANXA11 PFFs. Data are presented as mean ± SEM. Statistical significance was determined using Student’s* t*-test (**h**). Exact *P*-values are indicated in the corresponding graphs

Given that ANXA11 is an aggregation-prone protein and forms amyloid-like fibrils [4, 5], we first investigated whether neurons can internalize extracellular ANXA11 aggregates. ANXA11 PFFs labeled with the fluorescent dye Alexa Fluor 488 (PFFs-AF488) were added to SH-SY5Y cells. We confirmed the intracellular localization of ANXA11 aggregates in SH-SY5Y cells through F-actin labeling and confocal imaging (Fig. 1a). FCM analysis revealed that approximately 15.8% of the SH-SY5Y cells internalized AF488-labeled ANXA11 PFFs after 24 h of treatment (Fig. 1b).

To determine whether the internalized ANXA11 PFFs can trigger intracellular aggregation of cytosolic ANXA11, we assessed seeding in an ANXA11-GFP reporter system. While a slight upward trend was occasionally observed, PFF treatment alone did not induce a statistically significant increase in the detergent-insoluble ANXA11 (Fig. S1a, b). Consistently, HEK293T cells expressing ANXA11-GFP showed comparable aggregate distribution patterns in control and PFF-treated conditions, with no obvious changes in inclusion number and size (Fig. S1c). These findings suggested that, after internalization, a substantial fraction of incoming fibrils may remain compartmentalized from the cytosolic ANXA11 pool.

We next examined the subcellular localization of the internalized ANXA11 aggregates. During treatment, the PFFs-AF647 intensity on lysosomal-associated membrane protein 1 (LAMP1)-positive lysosomes increased in a time-dependent manner, and PFFs-AF647 and LAMP1 were highly colocalized after 24-h treatment (Fig. 1c, d). In contrast, only a small proportion of PFFs were localized on early endosome antigen 1 (EEA1)-positive early endosomes (Fig. S2a, b), suggesting that ANXA11 aggregates mainly accumulate in lysosomes after uptake. TEM revealed the presence of numerous low electron-density materials within lysosomes, accompanied by signs of mitochondrial damage in SH-SY5Y cells treated with PFFs (Fig. 1e).

While liposome-mediated delivery was initially utilized in our 2D assays to achieve rapid, synchronized intracellular accumulation of PFFs for acute biochemical detection, we rigorously evaluated whether this lysosomal damage could occur under physiological conditions without transfection reagents. Notably, prolonged incubation led to spontaneous endocytosis (passive uptake) of ANXA11 PFFs, which strongly triggered the recruitment of galectin-3 (GAL3) to ruptured lysosomes (Fig. S3a–c). Furthermore, this spontaneous internalization effectively triggered the recruitment of the autophagy marker LC3 (Fig. S3d–f). Importantly, treatment with the lipofection reagent alone failed to induce significant GAL3 or LC3 puncta formation. These results definitively confirm severe LMP and subsequent lysophagic responses as pathological consequences of ANXA11 fibril engulfment, rather than non-specific artifacts of lipid-mediated membrane perturbation.

### Accumulation of ANXA11 in lysosomes leads to lysosomal rupture

Lysosomes play a crucial role in the degradation and recycling of cellular material, which involves various enzymes, including proteases called cathepsins. Cathepsins are synthesized as inactive proenzymes, which are activated by autocleavage or other proteases into smaller mature forms in the acidic late endosomes and lysosomes [19]. To examine whether the accumulation of ANXA11 PFFs leads to the release of these proteases, we separated the PBS- or PFFs-treated SH-SY5Y cells into cytosolic and membrane fractions. Results showed that the mature cathepsins including CTSA, CTSB, and CTSD were released from membrane fraction into cytosol only upon ANXA11 PFFs treatment (Fig. 1f). To investigate the effects of ANXA11 PFF accumulation within lysosomes, we stained the cells with LysoTracker Red and observed a gradual reduction in both the intensity and the number of red lysosomal puncta (Fig. 1g, h). When lysosomes are ruptured, cytosolic lectins gain access to glycan groups on the intraluminal side of lysosomal membrane proteins; thus the lectin GAL3 is often used as a marker of lysosome damage [20]. We observed that some PFFs colocalized with GAL3 (Fig. 1i). Colocalization analysis showed GAL3 accumulation around lysosomes containing PFFs, indicating that lysosomes engulfing ANXA11 PFFs underwent membrane damage (Fig. 1j).

### ANXA11 PFFs trigger the RB1CC1-dependent lysophagy and autophagic flux

**Fig. 2:**
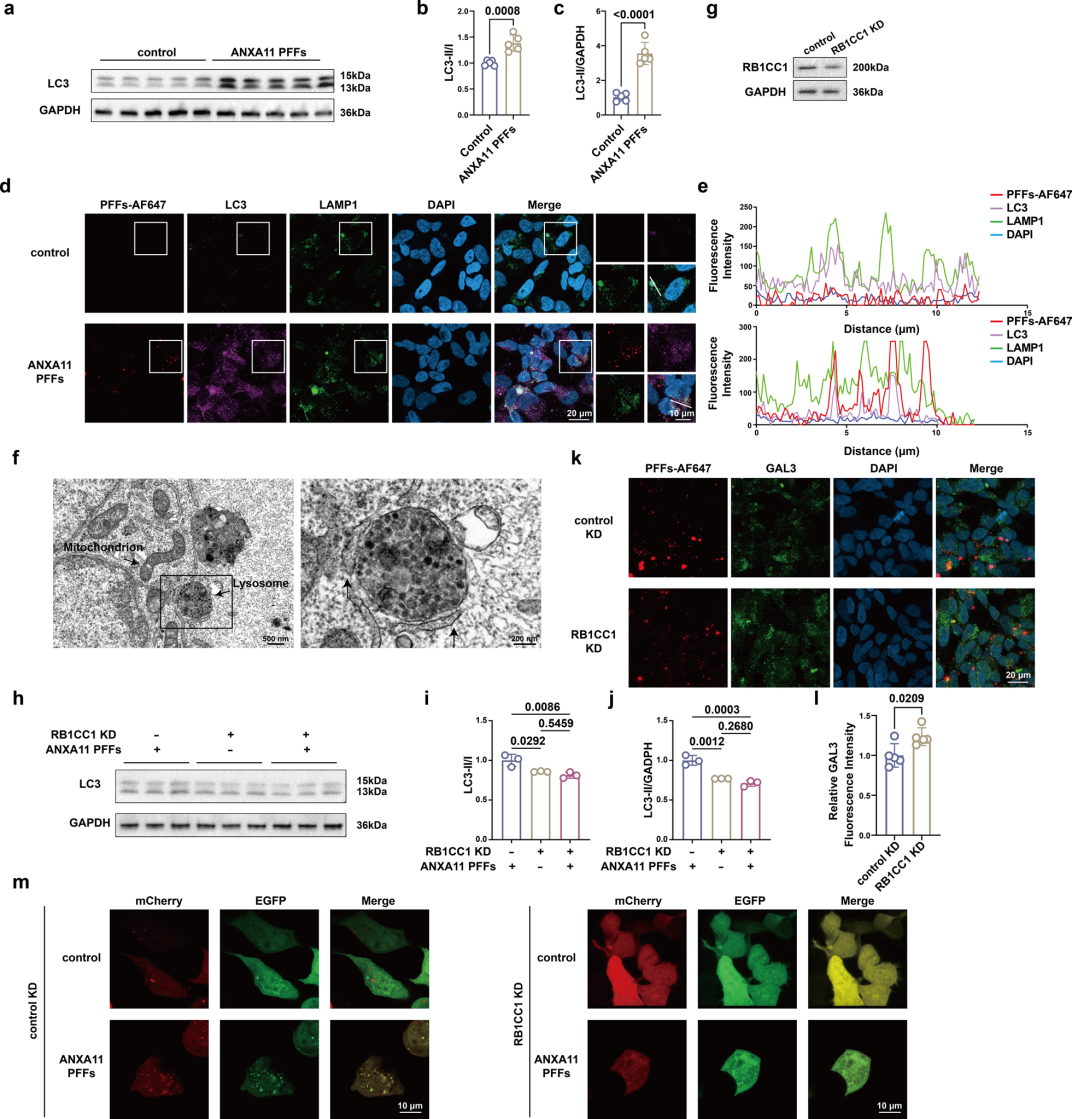
ANXA11 PFFs trigger RB1CC1-dependent lysophagy and autophagic flux.** a** Western blot analysis of LC3 levels in SH-SY5Y cells treated with or without ANXA11 PFFs. GAPDH served as a loading control. **b**, **c** Quantification of the LC3-II/LC3-I ratio (**b**) and LC3-II/GAPDH ratio (**c**) from the blots in (**a**), indicating increased autophagic activity upon PFFs treatment. **d** Representative confocal images showing the recruitment of LC3 (purple) to LAMP1-positive lysosomes (green) containing ANXA11 PFFs-AF647 (red). Nuclei were stained with DAPI (blue). **e** Fluorescence intensity profile analysis along the white line in (**d**), confirming the colocalization of ANXA11 PFFs, LAMP1, and LC3. **f** Transmission electron microscopy images showing double-membrane autophagosomes engulfing electron-dense lysosomal contents (arrows), a hallmark of lysophagy, in cells treated with ANXA11 PFFs. **g** Western blot confirming the knockdown efficiency of RB1CC1 in SH-SY5Y cells. **h** Western blot analysis of LC3 turnover in Control KD and RB1CC1 KD cells treated with or without ANXA11 PFFs. **i**, **j** Quantification of LC3-II/I (**i**) and LC3-II/GAPDH (**j**) ratios from (**h**), showing that RB1CC1 knockdown prevents the PFF-induced LC3 lipidation. **k** Immunofluorescence staining of galectin-3 (GAL3, green) in Control KD and RB1CC1 KD cells treated with ANXA11 PFFs-AF647 (red). **l** Quantification of relative GAL3 fluorescence intensity in (**k**). The increase in GAL3 signal in RB1CC1 KD cells indicates an accumulation of ruptured lysosomes due to impaired clearance. **m** Analysis of autophagic flux using the mCherry-EGFP-LC3 reporter. Control KD cells treated with PFFs show red puncta (indicating autolysosomes, where GFP is quenched by acidity), whereas RB1CC1 KD cells retain yellow/green fluorescence. Data are presented as mean ± SEM. Statistical significance was determined using Student’s *t*-test (**b, c**) or one-way ANOVA with Tukey’s post hoc test (**i, j, l**). Exact* P*-values are indicated in the corresponding graphs

Previous studies have established that damaged lysosomes are cleared by lysophagy, a selective form of autophagy [17]. Lysophagy can block the leakage of content from damaged lysosomes. Therefore, we investigated whether lysophagy prevents the escape of ANXA11 PFF seeds from damaged lysosomes. We observed that PFF treatment induced accumulation of microtubule-associated protein 1 light chain 3 (MAP1LC3/LC3) (Fig. 2a–c). This upregulation of LC3-II by PFF treatment was also observed in iPSC-derived neurons (Fig. S4a–c). Three-color images of LC3, LAMP1 and AF647-labeled ANXA11 PFFs demonstrated that the accumulated LC3 highly colocalized with LAMP1, suggesting that LC3 is assembled on lysosomes (Fig. 2d, e). Following treatment with ANXA11 PFFs and the autophagy inhibitor bafilomycin A1, TEM revealed the presence of double-membrane structures encapsulating electron-dense lysosomal contents, consistent with morphologic features of lysophagy (Fig. 2f).

To delineate whether the recruitment of LC3 to lysosomes is mediated specifically by lysophagy rather than noncanonical LC3 lipidation, we knockdown *RB1CC1* in SH-SYSY cells (*RB1CC1*-KD). This genetic ablation disrupts canonical autophagosome formation while leaving noncanonical LC3 lipidation intact. We found that in *RB1CC1*-KD cells (Fig. 2g, Fig. S4d, e), PFFs seldom induced LC3 accumulation on lysosomes (Fig. S4f, g). Immunoblots of LC3 also showed increased LC3-II levels with PFF treatment in WT cells but not in *RB1CC1*-KD cells (Fig. 2h–j). To determine whether lysophagy prevented the propagation of ANXA11 aggregation through ruptured lysosomes, we compared WT and *RB1CC1*-KD cells treated with PFFs. *RB1CC1*-KD cells showed a higher percentage of GAL3 dot-positive cells than WT cells when treated with PFFs, suggesting that damaged lysosomes remained abundant due to impaired lysophagic flux (Fig. 2k, l).

To directly monitor autophagic flux upon ANXA11 fibril exposure, we utilized the tandem fluorescent-tagged mCherry-EGFP-LC3 reporter (Fig. S4h). ANXA11 PFFs markedly increased red-only puncta, indicating enhanced autophagosome-lysosome fusion and progression of lysophagy. Conversely, this effect was abolished in *RB1CC1*-deficient cells (Fig. 2m).

### The ESCRT machinery mediates lysosomal membrane repair to mitigate the ANXA11 fibril-induced cytotoxicity

As lysosomal rupture can be extremely damaging to the cell, lysosomal quality control begins with an attempt to repair damaged lysosomes via the ESCRT machinery. In the absence of lysosomal repair, ruptured lysosomes are targeted for degradation via lysophagy [16, 21]. Therefore, ESCRT-mediated lysosomal repair precedes lysophagy, particularly in cases of less severe damage [22]. We next explored whether the ESCRT machinery also contributes to preventing the propagation of ANXA11 aggregation. To this end, we first examined whether the uptake of exogenous ANXA11 aggregates triggers the recruitment of the ESCRT machinery to lysosomes. Charged multivesicular body protein 2A (CHMP2A) and CHMP2B, core components of the ESCRT-III complex [23, 24], were confirmed to rapidly accumulate upon treatment with the L-leucyl-L-leucine methyl ester (LLOMe), a well-established lysosomotropic agent known to induce lysosomal membrane permeabilization [25, 26] (Fig. S5a-f), indicating prompt ESCRT recruitment in response to lysosomal damage. We next analyzed the subcellular distribution of ESCRT-III components following exposure to ANXA11 PFFs and observed recruitment of CHMP2A and CHMP2B precisely at LAMP1-positive lysosomes containing internalized fibrils (Fig. S6a, c). Fluorescence intensity profiling confirmed the precise spatial colocalization of ANXA11 aggregates, the lysosomal membrane, and ESCRT-III subunits (Fig. S6b, d). Co-immunoprecipitation assays revealed that ANXA11 physically interacts with ESCRT-III subunits CHMP2A and CHMP2B. Both proteins were specifically detected in GFP immunoprecipitates from ANXA11-GFP-expressing cells but not in control samples, indicating a specific association between ANXA11 and the ESCRT machinery (Fig. S7a). Morphological analysis further confirmed that the intracellular ANXA11 fibrils triggered the transition of CHMP2B into distinct punctate aggregates (Fig. S7b).

To establish the functional necessity of this repair mechanism, we disrupted the ESCRT pathway by knocking down ALIX and TSG101, key upstream factors required for ESCRT-III assembly [16, 27] (Fig. S5g-j, Fig. S7c). We hypothesized that compromising lysosomal repair would exacerbate membrane damage and necessitate a compensatory upregulation of lysophagy. Consistent with this, ALIX/TSG101-depleted cells exhibited a significant increase in GAL3 puncta accumulation upon PFF treatment compared to controls (Fig. S7d, e), indicating a failure to repair ruptured membranes. Furthermore, this exacerbation of lysosomal damage in ESCRT-deficient cells was accompanied by a marked elevation in LC3 recruitment to lysosomes (Fig. S7f, g). Collectively, these data demonstrate that the ESCRT machinery functions as a primary defensive barrier against ANXA11-induced lysosomal rupture, and its failure shifts the burden of quality control toward lysophagic clearance.

### The FTLD/ALS-linked ANXA11 D40G mutant exhibits enhanced lysosomal disruption and seeding capacity

Previous studies have indicated that the amino-terminal variant p.D40G, located within the low-complexity domain of ANXA11, enhances its aggregation propensity [8]. To determine whether the FTLD/ALS-associated D40G mutation affects the aggregation propensity of ANXA11, we purified recombinant wild-type (WT) and D40G mutant ANXA11 proteins and induced fibril formation in vitro. TEM revealed that both WT and D40G ANXA11 formed characteristic amyloid-like fibrils; however, the D40G fibrils appeared thicker and more densely packed than WT fibrils (Fig. S8a, b, upper panels). Atomic force microscopy (AFM) further confirmed that the D40G fibrils displayed a more intertwined network with increased height, indicating enhanced fibril compactness and rigidity (Fig. S8a, b, lower panels).

**Fig. 3:**
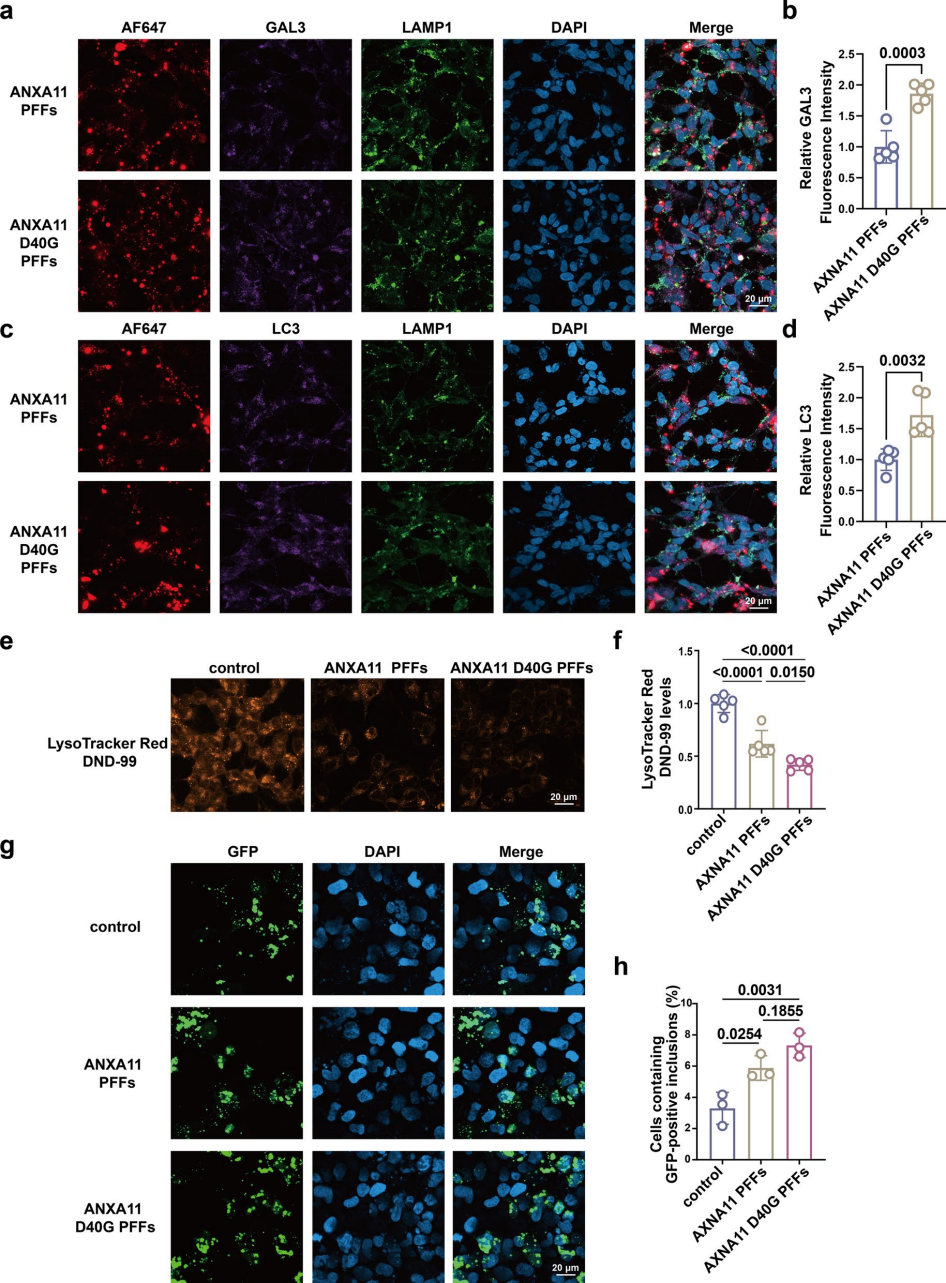
The FTLD/ALS-linked ANXA11 D40G mutant exhibits enhanced lysosomal disruption and seeding capacity.** a** Representative immunofluorescence images showing the accumulation of GAL3 (purple) puncta in SH-SY5Y cells treated with WT or D40G mutant ANXA11 PFFs-AF647 (red). Lysosomes were labeled with LAMP1 (green) and nuclei with DAPI (blue). **b** Quantification of relative GAL3 fluorescence intensity from (**a**). The results indicate that D40G fibrils induce more severe lysosomal membrane rupture compared to WT fibrils. **c** Representative confocal images displaying the recruitment of LC3 (purple) to LAMP1-positive lysosomes (green) containing internalized WT or D40G ANXA11 PFFs (red). **d** Quantification of relative LC3 fluorescence intensity from (**c**), showing enhanced autophagic response to D40G-induced damage. **e** Representative images of LysoTracker Red staining in cells treated with WT or D40G ANXA11 PFFs to assess lysosomal integrity. **f** Quantification of LysoTracker Red fluorescence intensity from (**e**). The D40G mutant caused a more pronounced reduction in lysosomal acidity. **g** Representative images of HEK293T cells expressing ANXA11-GFP treated with WT or D40G ANXA11 seeds, showing induced intracellular aggregation. **h** Quantification of the percentage of cells containing GFP-positive inclusions from (**g**). Data are presented as mean ± SEM. Statistical significance was determined using Student’s *t*-test (**b, d**) or one-way ANOVA with Tukey’s post hoc test (**f, h**). Exact* P*-values are indicated in the corresponding graphs

To determine whether the FTLD/ALS-associated D40G mutation alters the lysosomal response to ANXA11 fibrils, we compared lysosomal integrity and lysophagy activation between cells treated with WT and D40G ANXA11 PFFs. Immunofluorescence staining revealed a marked increase in GAL3-positive puncta in cells exposed to D40G fibrils compared with WT fibrils (Fig. 3a, b), indicating that the D40G mutant induces more severe lysosomal rupture. Consistent with enhanced lysosomal stress, we observed a pronounced increase in LC3 accumulation on LAMP1-positive lysosomes in cells treated with D40G fibrils (Fig. 3c, d). This suggested that lysophagy is more robustly activated in response to lysosomal damage induced by D40G fibrils. Together, these findings demonstrate that the D40G mutation exacerbates both lysosomal disruption and subsequent autophagic responses, providing a mechanistic explanation for the enhanced aggregate propagation potential of the mutant. The reduction of LysoTracker fluorescence intensity was even more pronounced in cells exposed to D40G fibrils, suggesting that the D40G mutation induces more severe impairment of lysosomal function (Fig. 3e, f).

Crucially, we investigated whether this exacerbated lysosomal failure facilitates the prion-like propagation of ANXA11. Using the ANXA11-GFP reporter system, we found that cells exposed to D40G seeds exhibited a higher frequency of induced intracellular aggregation compared to those treated with WT fibrils (Fig. 3g, h). Morphologically, these seeded aggregates appeared as highly irregular, solid-like inclusions. This pattern is distinctly different from the diffuse or spherical liquid-like state of unseeded ANXA11-GFP, indicating a liquid-to-solid phase transition templated by the internalized fibrils. Collectively, these data suggest that the D40G mutation enhances the structural stability of ANXA11 fibrils, thereby increasing their membranolytic potential and promoting the cytoplasmic escape required for efficient seeding of endogenous proteins.

To uncover the molecular mechanisms driving the enhanced lysosomal toxicity and accumulation of the D40G mutant, we performed bulk RNA sequencing on SH-SY5Y cells treated with WT or D40G ANXA11 PFFs (Fig. S9a). Transcriptomic analysis revealed that D40G fibrils induced a distinct gene expression profile compared to WT fibrils. Unlike a generalized stress response, the D40G mutation triggered highly specific transcriptional perturbations in pathways critical for lysosomal function (Fig. S9b). Specifically, we identified a significant downregulation of actin-related protein 10 (ACTR10), a dynactin subunit essential for the retrograde transport of lysosomes along microtubules. This suggests that D40G aggregates may mechanically and transcriptionally impede the transport of damaged lysosomes to the soma for degradation. Conversely, we observed upregulation of WNK1, a known negative regulator of autophagy, and ITPR2, an ER calcium release channel (Fig. S9c). These transcriptional changes indicate that the D40G mutation exerts a "multi-hit” toxicity. It not only physically ruptures lysosomes but also transcriptionally suppresses transport machinery and autophagic flux while exacerbating calcium dysregulation, thereby creating a vicious cycle of accumulation and toxicity.

### The FTLD/ALS-linked D40G mutation induces profound paralysis of axonal transport of RNA granules and lysosomes

To validate whether the D40G-specific transcriptional suppression of ACTR10 translates to a biologically relevant protein depletion, we assessed ACTR10 protein levels using confocal microscopy. Both SH-SY5Y cells and human iPSC-derived neurons showed a statistically significant reduction in ACTR10 fluorescence intensity following treatment with D40G ANXA11 PFFs, compared to both WT PFFs and controls (Fig. S9d–h). Fluorescence intensity profiling further corroborated the localized loss of ACTR10 structural integrity (Fig. S9e). Western blot analysis confirmed that the total cellular protein level of ACTR10 was specifically diminished in the D40G PFF-treated group (Fig. S9i, j). Given that ACTR10 is an indispensable dynactin subunit strictly required for the retrograde transport of organelles, its profound depletion at the protein level provides robust biochemical evidence supporting our "multi-hit" toxicity model, demonstrating that D40G fibrils actively dismantle the essential cellular transport machinery.

To determine whether this physical lysosomal damage and the D40G-driven depletion of ACTR10 translate into functional transport deficits, we evaluated the axonal transport machinery. High-resolution immunofluorescence analysis first revealed that exposure to ANXA11 PFFs induced an abnormal, clustered accumulation of LAMP1-positive lysosomes within MAP2-positive neurites, indicating a severe disruption of organelle trafficking (Fig. S10a). Because physiological ANXA11 functions as a tether for RNA granules, we performed dual-color live-cell imaging to test whether the PFF-induced damage causes a secondary collapse of this specific cargo transport. Kymograph analysis revealed that while control neurons exhibited robust, processive co-transport of ANXA11 and G3BP1 (RNA granules), PFF treatment completely abolished this directed movement, resulting in massive stationary aggregates and a functional paralysis of RNA granule transport (Fig. S10b, c).

Furthermore, we directly tracked lysosomal motility using LysoTracker Red in iPSC-derived neurons. While lysosomes in control neurons displayed active bidirectional axonal transport, exposure to WT PFFs significantly impaired their motility. Strikingly, treatment with the FTLD/ALS-linked D40G mutant induced nearly complete paralysis of lysosomal trafficking, evidenced by an overwhelming fraction of stationary events and a severely reduced average velocity (Fig. S10d-f). Together, these live-cell functional assays provide definitive evidence that D40G aggregates orchestrate a "multi-hit" collapse of the neuronal transport machinery, corroborating our transcriptomic and biochemical findings.

### Lysosomal damage by ANXA11 fibrils triggers the p38/MK2/HSP27 signaling axis to promote lysophagy

**Fig. 4:**
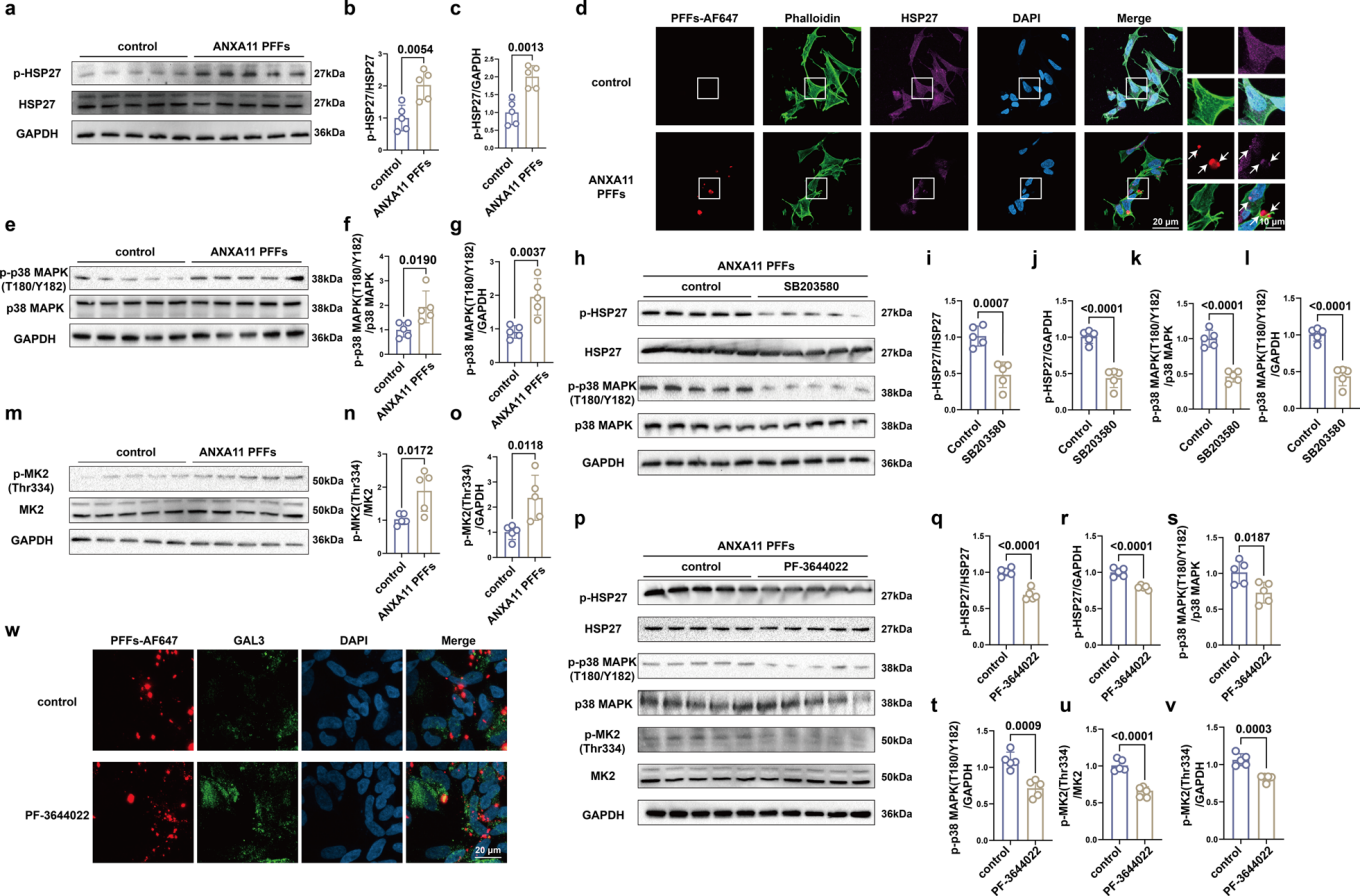
Lysosomal damage by ANXA11 fibrils activates the p38/MK2/HSP27 signaling axis to promote lysophagy.** a** Western blot analysis of phosphorylated HSP27 (p-HSP27) and total HSP27 expression in SH-SY5Y cells treated with or without ANXA11 PFFs. GAPDH was used as a loading control. **b**, **c** Quantification of p-HSP27 levels normalized to total HSP27 (**b**) and GAPDH (**c**) from (**a**). **d** Representative immunofluorescence images showing the formation of HSP27 puncta (purple) and their colocalization with ANXA11 PFFs-AF647 (red). Phalloidin (green) stains F-actin and DAPI (blue) stains nuclei. Arrows indicate colocalization sites. **e** Western blot analysis of phosphorylated p38 MAPK (p-p38, T180/Y182) and total p38 MAPK levels. **f**, **g** Quantification of p-p38 levels normalized to total p38 (**f**) and GAPDH (**g**) from (**e**). **h** Western blot analysis of p-HSP27 and p-p38 MAPK levels in cells treated with ANXA11 PFFs in the presence or absence of the p38 MAPK inhibitor SB203580. **i**–**l** Quantification of relative protein levels from (**h**), showing that p38 inhibition effectively blocks the phosphorylation of HSP27. **m** Western blot analysis of phosphorylated MK2 (p-MK2, Thr334) and total MK2 levels. **n**, **o** Quantification of p-MK2 levels normalized to total MK2 (**n**) and GAPDH (**o**) from (**m**). **p** Western blot analysis of the p38/MK2/HSP27 pathway in cells treated with ANXA11 PFFs in the presence or absence of the MK2 inhibitor PF-3644022. **q–v** Quantification of phosphorylation levels from (**p**). Inhibition of MK2 significantly reduces the phosphorylation of HSP27 and upstream signaling components. **w** Representative immunofluorescence images of galectin-3 (GAL3, green) in cells treated with ANXA11 PFFs-AF647 (red) in the presence or absence of the p38 inhibitor PF-3644022. The increase in GAL3 puncta upon inhibition indicates exacerbated lysosomal damage due to impaired protective signaling. Data are presented as mean ± SEM. Data are presented as mean ± SEM. Statistical significance was determined using Student’s *t*-test (**b, c, f, g, i, j, k, l, n, o, q, r, s, t, u, v**). Exact* P*-values are indicated in the corresponding graphs

Having established that ANXA11 PFFs induce lysosomal damage and subsequent lysophagy, we sought to investigate the upstream signaling mechanisms that initiate this protective response. Previous studies have reported that HSP27 is rapidly recruited to sites of lysosomal damage and participates in the initiation of lysophagy [28]. HSP27 is recognized for its protective role in neurodegeneration by maintaining aggregation-prone proteins in a folding-competent state, facilitating phase separation, and performing diverse functions including anti-apoptotic and anti-oxidant activities, regulating cytoskeletal dynamics, and sustaining mitochondrial proteostasis [29, 30]. HSP27 is phosphorylated at serine 15, 78, and 82 in response to oxidative stress and hyperosmotic stress [31, 32]. Therefore, we investigated whether the ANXA11 fibril-induced lysosomal damage promotes HSP27 phosphorylation. We found that ANXA11 fibril treatment robustly and significantly enhanced HSP27 phosphorylation (Fig. 4a–c), and that HSP27 formed robust puncta in cells upon treatment with ANXA11 fibrils (Fig. 4d).

In other cellular stress contexts that involve lysophagy, phosphorylation of HSP27 has been observed downstream of p38 MAPK α/β activation [33]. This prompted us to investigate whether ANXA11 fibrils induce phosphorylation of p38 MAPK at T180/Y182. We observed a significant increase in phosphorylated, active p38 MAPK that was induced by lysosomal stress in SH-SY5Y cells (Fig. 4e–g). Treatment with SB203580, an ATP-competitive inhibitor of p38 MAPK [34], markedly reduced the phosphorylation levels of both HSP27 and p38 MAPK (Fig. 4h–l).

MAPKAPK2 or MK2 is a downstream effector kinase of p38 MAPK known to phosphorylate HSP27 in response to heat or oxidative stress [32]. Therefore, to evaluate whether MK2 is similarly activated in the cellular stress response induced by ANXA11 fibrils, we employed a phospho-specific antibody targeting Threonine 334 (T334) (Fig. 4m–o). To further confirm that MK2 acts downstream of p38 MAPK to mediate HSP27 activation, we employed PF-3644022, a potent and highly selective ATP-competitive inhibitor of MK2 [35], to pharmacologically block MK2 activity in ANXA11 PFF-treated cells. As expected, treatment with the MK2 inhibitor PF-3644022 abrogated the phosphorylation of p38 MAPK, MK2, and HSP27 in response to lysosomal damage (Fig. 4p–v). Moreover, GAL3 staining showed that inhibition by PF-3644022 resulted in a marked increase in damaged lysosomes (Fig. 4w).

To definitively establish the causal and protective role of this signaling axis, we performed gain-of-function experiments through lentivirus-mediated overexpression of HSP27 (OE-HSP27). In SH-SY5Y cells, OE-HSP27 significantly attenuated the ANXA11 PFF-induced lysosomal rupture, as evidenced by a marked reduction in GAL3 puncta and the successful restoration of lysosomal acidification (Fig. S11a–d). Furthermore, we investigated whether boosting this protective chaperone response could halt the prion-like spreading of ANXA11. Utilizing our neuron-to-neuron propagation model, we transduced recipient iPSC-derived neurons with HSP27 (Fig. S11e). Remarkably, OE-HSP27 in recipient neurons not only rescued lysosomal integrity (Fig. S11f, g), but also drastically reduced the accumulation of internalized ANXA11 seeds (Fig. S11h). Collectively, these gain-of-function results demonstrate that targeted activation of the HSP27 axis is sufficient to preserve lysosomal function and restrict the intercellular propagation of ANXA11 aggregates.

### Lysophagy impairment and autophagy deficiency synergize to markedly enhance ANXA11 seeding

**Fig. 5:**
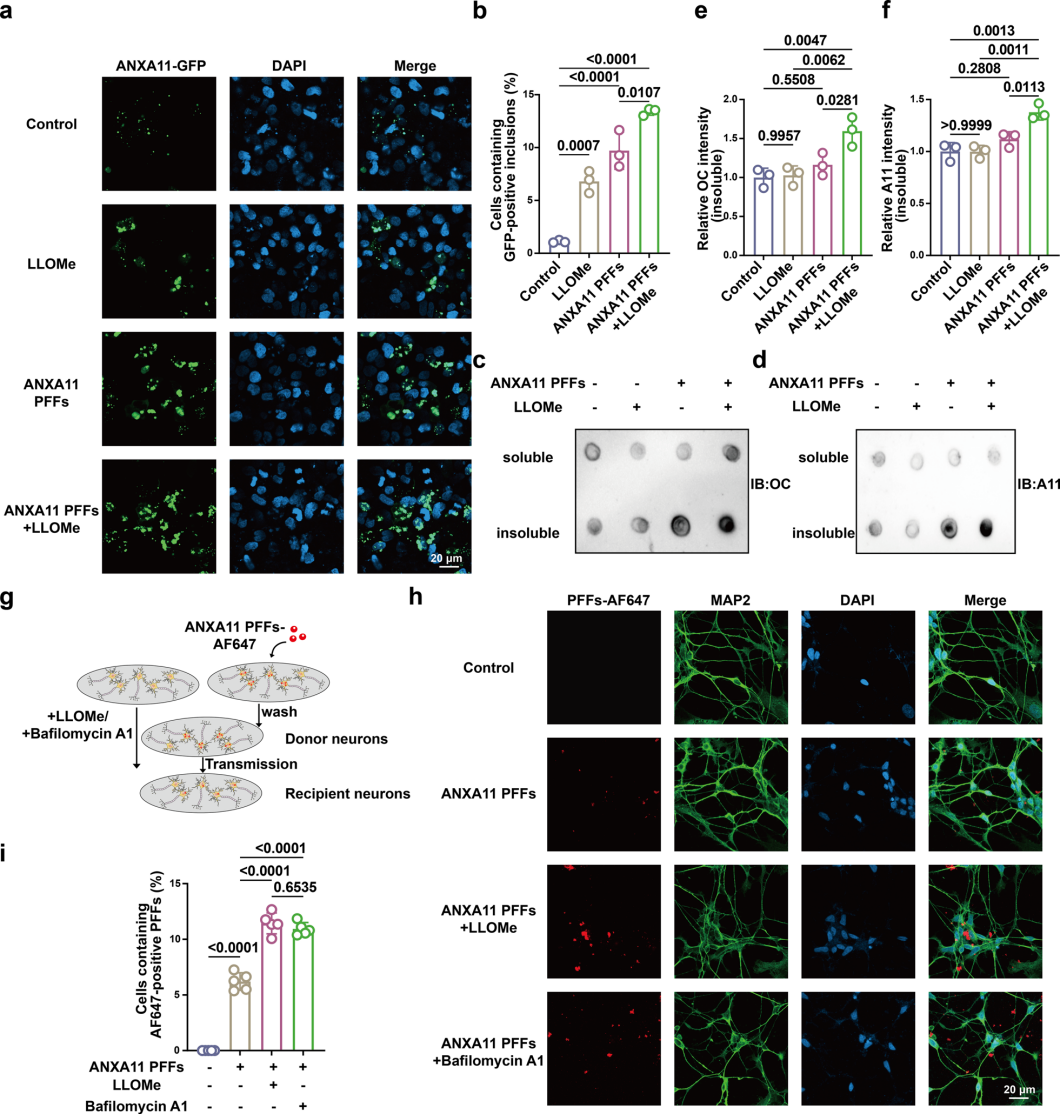
Lysosomal rupture and autophagy deficiency synergistically promote the cytoplasmic seeding and neuronal propagation of ANXA11 aggregates.** a** Representative confocal microscopy images of SH-SY5Y cells stably expressing ANXA11-GFP. Cells were treated with ANXA11 PFFs (5 µg/mL) for 24 h, followed by exposure to the lysosomotropic agent LLOMe (1 mM) to induce lysosomal membrane permeabilization. Nuclei were counterstained with DAPI (blue). Note the marked increase in intracellular ANXA11-GFP inclusions in the combined treatment group. **b** Quantification of the percentage of cells containing ANXA11-GFP positive inclusions from the experiment in (**a**). **c, d** Dot blot analysis of cell lysates fractionated into Triton X-100 soluble and insoluble fractions. Membranes were probed with the conformation-dependent antibodies OC (targeting amyloid fibrils) (**c**) and A11 (targeting prefibrillar oligomers) (**d**). **e, f** Densitometric quantification of the relative signal intensity of OC (**e**) and A11 (**f**) in the insoluble fractions, normalized to controls. **g** Schematic illustration of the neuron-to-neuron transmission assay. Donor iPSC-derived neurons were loaded with AF647-labeled ANXA11 PFFs, washed to remove extracellular fibrils, and the conditioned medium/lysate was transferred to recipient neurons. Recipient cells were subsequently treated with LLOMe or BafA1 to assess the impact of lysosomal integrity and autophagic flux on aggregate propagation. **h** Representative confocal immunofluorescence images of recipient iPSC-derived neurons stained for the neuronal marker MAP2 (green). Internalized intracellular ANXA11 PFFs-AF647 are shown in red. The confocal imaging ensures that the red fluorescent signals represent genuine intracellular aggregates rather than extracellular membrane-bound particles. **i** Quantification of the percentage of recipient neurons containing ANXA11 PFFs-positive aggregates. The data demonstrate that both lysosomal rupture (LLOMe) and autophagy inhibition (BafA1) significantly enhance the accumulation of pathological seeds in recipient neurons. Data are presented as mean ± SEM. Statistical significance was determined using one-way ANOVA with Tukey’s post hoc test (**b, e, f, i**). Exact *P*-values are indicated in the graphs

We propose that lysosomes serve as a critical barrier sequestering internalized fibrils from the cytosolic pool of naïve ANXA11. To experimentally test whether lysosomal rupture is the rate-limiting step for pathological seeding, we utilized a "break-and-seed" assay. SH-SY5Y cells expressing ANXA11-GFP were exposed to PFFs, followed by treatment with the lysosomotropic agent LLOMe to induce widespread membrane permeabilization. While PFFs alone induced moderate intracellular aggregation, the pharmacological disruption of lysosomal integrity triggered a synergistic and massive increase in ANXA11-GFP inclusion formation (Fig. 5a, b). These massively induced inclusions exhibited a distinctly irregular, solid-like morphology, further supporting a catastrophic liquid-to-solid phase transition of the cytosolic ANXA11 pool driven by the escaped seeds.

To characterize the structural nature of these induced aggregates, we performed dot blot analyses using conformation-specific antibodies. The combination of PFFs and lysosomal rupture resulted in a dramatic increase of both OC-reactive species (indicating mature amyloid fibrils) and A11-reactive species (indicating toxic oligomers) in the insoluble fraction (Fig. 5c–f). These data rigorously demonstrate that lysosomal leakage facilitates the contact between internalized seeds and endogenous proteins, thereby catalyzing templated misfolding.

We examined whether the same phenomenon occurs in a neuronal model by using iPSC-derived neurons to assess the intercellular transmission of ANXA11 aggregates. To investigate this process, we established a co-culture system that enables specific assessment of the mechanisms underlying cell-to-cell transmission in neuronal cells and allows clear distinction between donor and recipient neurons (Figs. 5g and S12). In this experiment, donor neurons were treated with Alexa Fluor 647-labeled ANXA11 PFFs (PFFs-AF647), which were subsequently transmitted to and internalized by the recipient neurons. Following stringent washing steps to remove residual extracellular seeds, confocal Z-stack optical sectioning confirmed that the transmitted PFFs-AF647 were localized intracellularly within the recipient MAP2-positive neurons. We monitored the propagation of AF647-labeled ANXA11 fibrils into recipient neurons under conditions of lysosomal stress. Consistent with our cell line data, inducing lysosomal rupture with LLOMe significantly enhanced the accumulation of PFFs within recipient MAP2-positive neurons (Fig. 5h, i). Crucially, we asked whether the failure of the repair/clearance machinery mimics this effect. Pharmacological blockade of autophagic flux using Bafilomycin A1 resulted in a comparable increase in fibril burden (Fig. 5h, i).

### ANXA11 PFFs induce lysosomal membrane permeabilization-dependent mitochondrial dysfunction, oxidative stress, and caspase-3-mediated apoptosis

To determine the cellular consequences of lysosomal damage induced by ANXA11 fibrils, we next examined whether LMP triggers downstream mitochondrial dysfunction and apoptotic signaling.

**Fig. 6:**
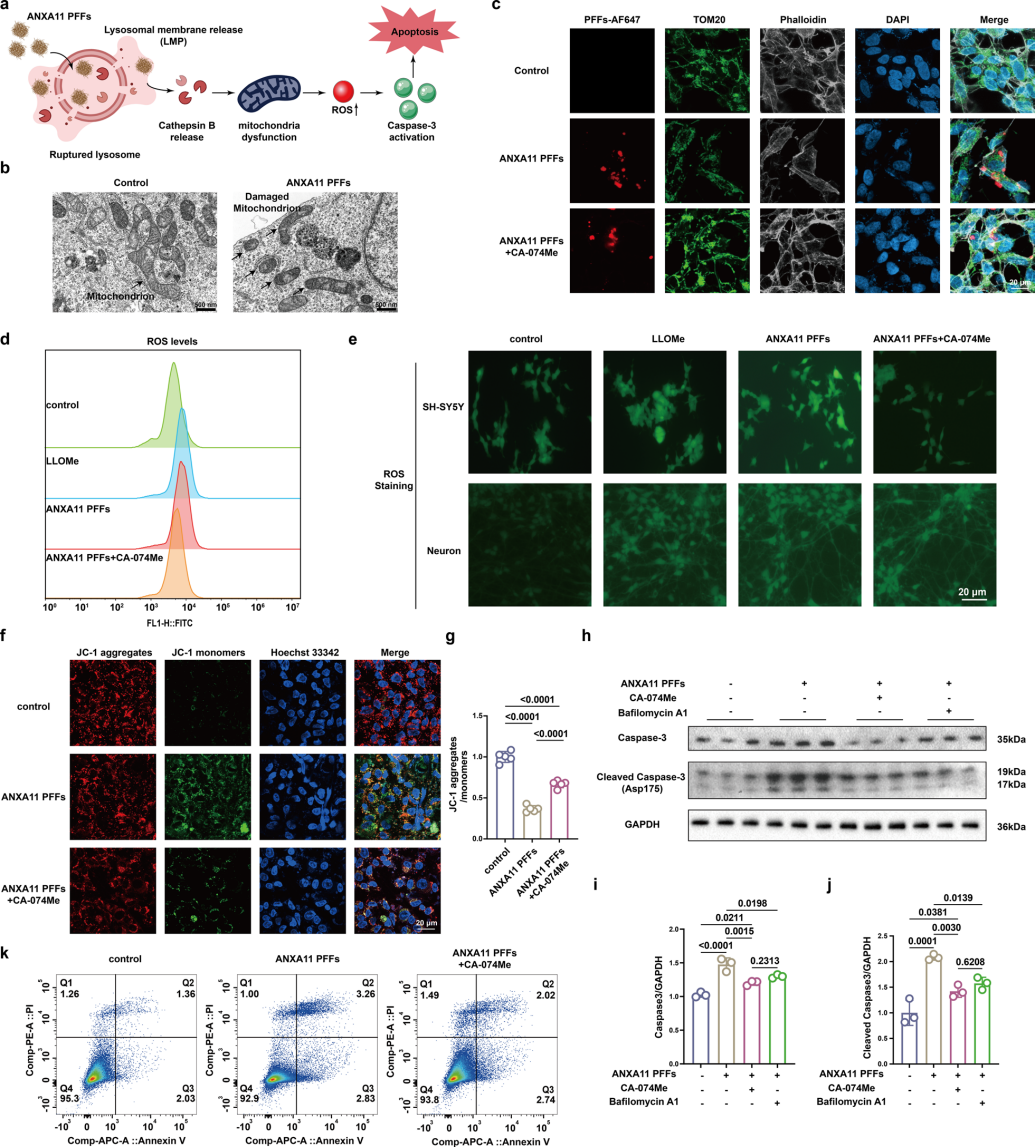
ANXA11 fibril-induced lysosomal rupture triggers a cathepsin B-dependent cascade of mitochondrial dysfunction, oxidative stress, and apoptosis.** a** Schematic illustration of the proposed cytotoxic mechanism: lysosomal membrane permeabilization (LMP) induced by ANXA11 fibrils leads to the cytosolic release of cathepsin B, which subsequently triggers mitochondrial dysfunction, ROS accumulation, and Caspase-3-mediated apoptosis. **b** Representative transmission electron microscopy images of SH-SY5Y cells. Control cells exhibit healthy mitochondria with intact cristae, whereas ANXA11 PFFs-treated cells display swollen mitochondria with disrupted cristae (black arrows). **c** Representative confocal images showing mitochondrial morphology. Cells were stained for the mitochondrial outer membrane marker TOM20 (green), F-actin (Phalloidin, white), and nuclei (DAPI, blue). ANXA11 PFFs (red) induce mitochondrial fragmentation and network disruption. **d** Flow cytometric analysis of intracellular reactive oxygen species (ROS) levels using the DCFH-DA probe. Treatment with the specific Cathepsin B inhibitor CA-074Me attenuates the ROS surge induced by ANXA11 PFFs. **e** Representative fluorescence images of ROS generation in SH-SY5Y cells and iPSC-derived neurons. The green signal represents the fluorescence emitted by the oxidized DCF probe, visually indicating the levels of intracellular ROS. These images confirm the induction of oxidative stress upon PFFs exposure and its mitigation by lysosomal protection. **f** Assessment of mitochondrial membrane potential using JC-1 staining. In healthy cells, JC-1 forms red aggregates; in depolarized mitochondria, it exists as green monomers. ANXA11 PFFs induce a shift toward green fluorescence (depolarization), which is prevented by CA-074Me. **g** Quantification of the JC-1 aggregate-to-monomer fluorescence ratio from (**f**). **h** Western blot analysis of apoptotic signaling. ANXA11 PFFs increase levels of cleaved Caspase-3 (Asp175). This activation is partially blocked by inhibiting cathepsin B (CA-074Me) or autophagic flux (Bafilomycin A1). GAPDH served as a loading control. **i, j** Densitometric quantification of total Caspase-3 (**i**) and cleaved Caspase-3 (**j**) levels normalized to GAPDH. **k** Flow cytometric quantification of apoptosis using Annexin V-PE/Propidium Iodide (PI) double staining. The proportion of early (Q3) and late (Q2) apoptotic cells was significantly increased by ANXA11 PFFs but rescued by CA-074Me treatment. Data are presented as mean ± SEM. Statistical significance was determined using one-way ANOVA with Tukey’s post hoc test (**g**, **i**, **j**). Exact *P*-values are indicated in the graphs

TEM revealed preserved mitochondrial ultrastructure in control cells, whereas the ANXA11 PFF-treated cells exhibited swollen mitochondria with disrupted cristae, indicative of mitochondrial damage (Fig. 6b). Consistent with these ultrastructural changes, confocal imaging showed that the internalized ANXA11 PFFs showed a disrupted spatial association with the mitochondrial network labeled by TOM20, compared to control cells, accompanied by pronounced cytoskeletal rearrangement (Fig. 6c). Importantly, pharmacological inhibition of lysosomal rupture by CA-074Me markedly attenuated these mitochondrial abnormalities, suggesting that mitochondrial dysfunction occurs downstream of LMP.

We next assessed oxidative stress following ANXA11 PFF exposure. Flow cytometric analysis using DCFH-DA revealed a robust increase in intracellular ROS levels in ANXA11 PFF-treated cells, comparable to that induced by the LLOMe (Fig. 6d). This elevation in ROS was significantly reduced by CA-074Me, indicating that oxidative stress is largely dependent on lysosomal disruption. Fluorescence imaging confirmed that exposure to ANXA11 PFFs or LLOMe triggered a robust increase in intracellular green fluorescence, which represents oxidized DCF generated by elevated ROS levels. Importantly, this PFF-induced oxidative stress was mitigated by the Cathepsin B inhibitor CA-074Me in both SH-SY5Y cells and iPSC-derived neurons (Fig. 6e).

To determine whether mitochondrial functional integrity was compromised, we examined mitochondrial membrane potential using JC-1 staining. Control cells primarily exhibited JC-1 aggregates, reflecting intact membrane potential, whereas ANXA11 PFF treatment caused a marked shift toward JC-1 monomers, indicative of mitochondrial depolarization (Fig. 6f, g). This effect was substantially rescued by inhibition of lysosomal cysteine protease activity, further supporting a causal link between LMP and mitochondrial dysfunction.

Given the convergence of mitochondrial impairment and oxidative stress, we next evaluated activation of the intrinsic apoptotic pathway. Immunoblot analysis demonstrated that ANXA11 PFFs significantly increased the levels of cleaved caspase-3, while the total caspase-3 level was moderately elevated (Fig. 6h-j). Pharmacological inhibition of lysosomal rupture by CA-074Me or blockade of autophagic flux by bafilomycin A1 partially attenuated caspase-3 activation, indicating that lysosomal damage and impaired lysosomal clearance jointly contribute to apoptotic signaling.

Finally, apoptotic cell death was quantified by Annexin V/propidium iodide staining. Flow cytometric analysis revealed a significant increase in early and late apoptotic populations following ANXA11 PFF treatment, whereas CA-074Me significantly reduced apoptosis induction (Fig. 6k). Collectively, these results demonstrate that the ANXA11 fibril-induced lysosomal membrane permeabilization initiates a pathological cascade involving mitochondrial dysfunction, oxidative stress, and caspase-3-dependent apoptosis (Fig. 6a).

### ANXA11 PFFs are engulfed by lysosomes in cerebral organoids, leading to lysosomal damage and apoptosis

**Fig. 7:**
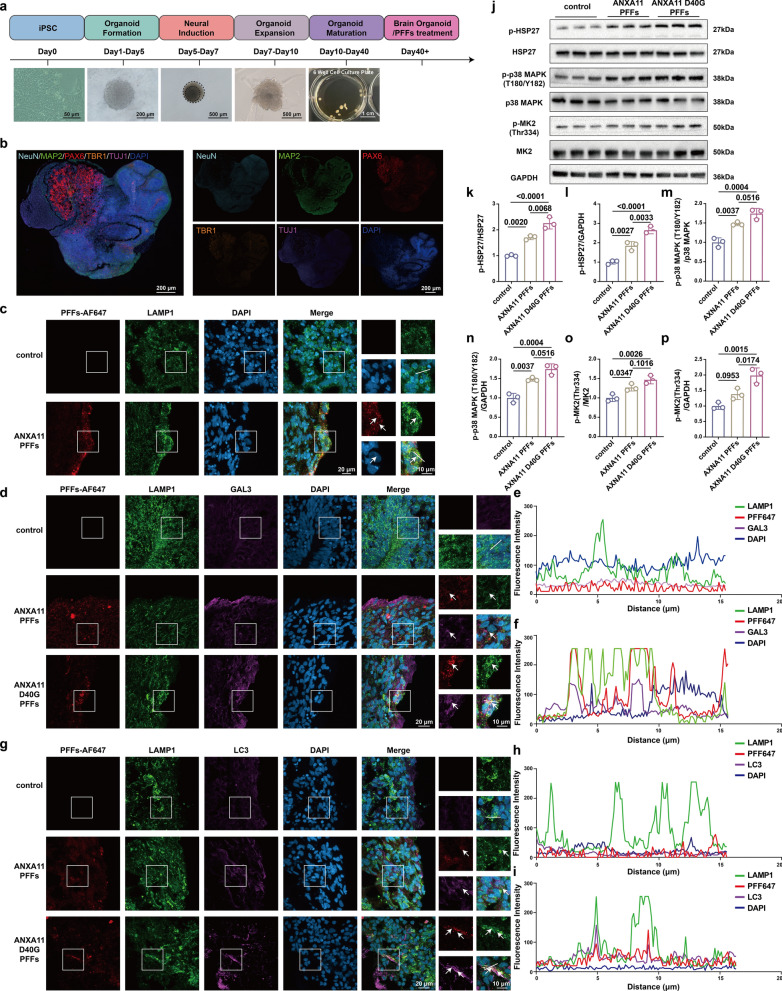
The FTLD/ALS-linked D40G mutation exacerbates lysosomal rupture and drives a hyperactive lysophagic response in human cerebral organoids.** a** Schematic timeline illustrating the generation of human iPSC-derived cerebral organoids and the experimental window for ANXA11 PFFs treatment (Day 40 +). **b** Multiplex immunofluorescence characterization of Day 40 organoids. The tissue exhibits a complex cytoarchitecture containing PAX6^+^ neural progenitors (red), TUJ1^+^ early neurons (purple), MAP2^+^ mature neurons (green), TBR1^+^ deep-layer cortical neurons (orange), and NeuN^+^ neuronal nuclei (white/cyan). Nuclei are counterstained with DAPI (blue). **c** Representative confocal images showing the internalization of AF647-labeled ANXA11 PFFs (red) into the organoid parenchyma and their specific colocalization with LAMP1^+^ lysosomes (green). **d** Assessment of lysosomal membrane permeabilization (LMP). Organoids were treated with WT or D40G ANXA11 PFFs. Note the widespread accumulation of galectin-3 (GAL3, purple) puncta on lysosomes in the D40G group, indicating severe membrane rupture. **e**, **f** Fluorescence intensity profile analyses along the white lines in (**d**), demonstrating the recruitment of GAL3 to lysosomes containing WT (**e**) and D40G (**f**) fibrils. The D40G profile shows a higher degree of signal overlap. **g** Evaluation of the lysophagic response. Immunostaining revealed the recruitment of the autophagy marker LC3 (purple) to LAMP1^+^ lysosomes (green) containing ANXA11 aggregates (red). **h**, **i** Fluorescence intensity profile analyses along the white lines in (**g**)**,** confirming the colocalization of LC3 with ruptured lysosomes in WT (**h**) and D40G (**i**) treated organoids. **j** Western blot analysis of the p38/MK2/HSP27 signaling axis in organoid lysates following treatment with WT or D40G ANXA11 PFFs. GAPDH served as a loading control. **k**–**p** Densitometric quantification of the phosphorylation levels of HSP27 (**k**, **l**), p38 MAPK (**m**, **n**), and MK2 (**o**, **p**). The data indicate that the D40G mutant induces a significantly more robust activation of this protective stress response pathway compared to WT fibrils. Data are presented as mean ± SEM. Statistical significance was determined using one-way ANOVA with Tukey’s post hoc test (**k**, **l**, **m**, **n**, **o**, **p**). Exact *P*-values are indicated in the graphs

To validate the translational relevance of our findings within a complex tissue architecture, we generated human iPSC-derived cerebral organoids (Fig. 7a). Multiplex immunohistochemistry imaging confirmed the successful development of laminated neural structures containing PAX6^+^ progenitors and MAP2^+^/TBR1^+^/NeuN^+^ mature neurons (Figs. 7b and S13a), establishing a physiologically relevant model for FTLD pathology.

We next investigated whether the spatial containment and lysosomal targeting of ANXA11 fibrils are conserved in 3D tissue. Following incubation, internalized ANXA11 PFFs penetrated the organoid parenchyma and accumulated specifically within LAMP1-positive compartments (Fig. 7c). Fluorescence intensity profiling confirmed the precise sequestration of fibrils within the lysosomal lumen (Fig. S13b, c).

Crucially, we assessed whether the organoid model recapitulates the mutation-dependent lysosomal toxicity observed in monolayer cultures. Immunofluorescence staining for GAL3 revealed that ANXA11 fibrils induced widespread LMP within the neuronal layers (Fig. 7d). Quantitative analysis demonstrated that the FTLD/ALS-linked D40G mutant triggered a significantly more severe disruption of lysosomal integrity compared to WT fibrils, as evidenced by the higher frequency and intensity of GAL3 puncta (Fig. S13c).

Consistently, the D40G mutant elicited a robust upregulation of the lysophagic response. We observed a marked accumulation of LC3 on ruptured lysosomes containing D40G aggregates, significantly exceeding the response triggered by WT fibrils (Figs. 7g and S13d). Furthermore, biochemical analysis of organoid lysates confirmed that this intensified cellular stress translated into hyperactivation of the protective signaling axis: phosphorylation levels of p38 MAPK, MK2, and HSP27 were significantly elevated in organoids exposed to D40G fibrils (Fig. 7j–p).

To determine whether the severe lysosomal damage and hyperactive stress response induced by D40G fibrils ultimately lead to neuronal loss in a complex tissue environment, we assessed cell death using Terminal deoxynucleotidyl transferase dUTP nick end labeling (TUNEL) staining. Consistent with our findings in monolayer cultures, organoids exposed to D40G PFFs exhibited extensive apoptotic cell death. Quantitative analysis revealed a significantly higher frequency of TUNEL-positive nuclei in the D40G-treated group compared to those treated with WT fibrils or vehicle control (Fig. S14a, b). This result confirmed that the D40G mutation confers a heightened neurotoxicity that compromises neuronal survival within 3D brain-like tissues, mirroring the aggressive neurodegeneration observed in FTLD patients.

Collectively, these data demonstrate that human cerebral organoids recapitulate the critical pathological hierarchy of ANXA11 proteinopathy—uptake, membrane rupture, and consequent lysophagic induction—and provide tissue-scale evidence that the D40G mutation acts as a potent accelerator of lysosomal failure.

## Discussion

The identification of *ANXA11* mutations in FTLD/ALS has expanded the genetic landscape of multisystem proteinopathies [8, 36, 37]; however, the cellular mechanisms linking ANXA11 aggregation to neuronal death and progressive spreading remain elusive. In this study, we provide evidence that the endolysosomal system is impaired in ANXA11 proteinopathy. We demonstrate that the internalized ANXA11 amyloid fibrils, particularly those harboring the FTLD/ALS-linked D40G mutation, act as potent damaging agents that rupture lysosomes. This event triggers a dual response: a toxic cascade involving cathepsin release and mitochondrial dysfunction, and a protective response orchestrated by the p38/MK2/HSP27 signaling axis to initiate lysophagy. Crucially, utilizing human iPSC-derived neurons and cerebral organoids, we establish that lysophagy functions as a pivotal checkpoint. Failure of this quality control mechanism allows ANXA11 seeds to escape into the cytoplasm, thereby accelerating the intercellular transmission and templated misfolding of endogenous proteins.

While lipid-mediated transfection is frequently employed in in vitro models to synchronize fibril internalization and achieve sufficient intracellular concentrations for high-resolution dynamic imaging, this method bypasses the physiological endocytic pathway and can induce artificial membrane perturbation. To validate the physiological relevance of our findings, we demonstrated that spontaneous endocytosis of naked ANXA11 PFFs successfully recapitulated the subsequent lysophagic response, as evidenced by endogenous GAL3 recruitment. This physiological uptake route and the resulting endolysosomal rupture closely mirror the established prion-like propagation mechanisms of other amyloidogenic proteins, such as α-synuclein and tau [13, 38].

However, distinguishing our study from generic models of amyloid toxicity, the downstream physiological consequence of ANXA11-induced LMP is profoundly unique. ANXA11 functions as an essential molecular tether connecting RNA granules to motile lysosomes for long-distance axonal transport [6]. Our live-cell imaging revealed that the ANXA11 PFF-induced endolysosomal damage not only triggers a local lysophagic clearance response, but also leads to a catastrophic secondary failure of the RNA granule transport machinery. This dual-hit mechanism, prion-like endolysosomal rupture coupled with the specific functional collapse of RNA tethering, highlights a novel, ANXA11-specific pathway in the pathogenesis of neurodegeneration that extends beyond traditional synuclein-focused models.

Consistent with this dual-hit mechanism, our work characterized LMP as the primary driver of ANXA11-mediated neurotoxicity. Although lysosomes function as the terminal degradation station for endocytosed cargoes [13, 39], our data indicate that the rigid, amyloid-like structure of ANXA11 PFFs overwhelms this organelle's capacity. The consequence is catastrophic: the leakage of luminal cathepsins (CTSB, CTSD) into the cytosol, which was observed to precede mitochondrial depolarization and ROS accumulation. Crucially, we validated that this severe LMP and the ensuing lysophagic clearance occurred independently of Lipofectamine 3000-mediated delivery. Spontaneous endocytosis of ANXA11 PFFs in both 2D cultures and complex 3D cerebral organoids consistently triggered these pathological phenotypes, confirming that the 'inside-out' lysosomal rupture is a physiologically relevant mechanism driving fibril toxicity and intercellular spreading. This lysosome-mitochondria axis of toxicity suggests a convergent mechanism for neurodegeneration induced by internalized amyloid fibrils [40–42]. However, distinct from soluble oligomers that may form pores, our ultrastructural analysis suggests that ANXA11 fibrils physically disrupt the membrane integrity. This process is significantly exacerbated by the D40G mutation [42]. The enhanced rigidity and compactness of D40G fibrils, as revealed by our AFM data, likely make them more disruptive to the lysosomal membrane and more resistant to enzymatic degradation, providing a mechanistic rationale for the aggressive clinical phenotype associated with this mutation.

Beyond the physical disruption of lysosomal membranes, our transcriptomic analysis revealed that the D40G mutation imposes a specific transcriptional block on lysosomal quality control. We identified a significant downregulation of ACTR10, a crucial adaptor for the dynein-dynactin complex. Since retrograde transport is essential for delivering damaged lysosomes to the soma for degradation, the suppression of ACTR10 likely causes the "traffic jam" of aggregate-filled lysosomes we observed in neurites [43]. Furthermore, the concomitant upregulation of WNK1 and ITPR2 creates a maladaptive environment. WNK1 is known to inhibit autophagy initiation [44], while ITPR2 upregulation could sensitize cells to calcium overload [45], a phenomenon consistent with the severe mitochondrial depolarization observed downstream of lysosomal rupture. Thus, the enhanced toxicity of D40G fibrils is not merely a consequence of their rigid structure, but stems from a dual mechanism: physical membrane abrasion and a transcriptional reprogramming that cripples the neuron’s transport and repair machinery.

Mechanistically, we identified the specific signaling pathway that couples lysosomal damage to the autophagy machinery. We show that lysosomal rupture by ANXA11 fibrils activates p38 MAPK and its downstream effector MK2, resulting in the phosphorylation of HSP27. The phosphorylated HSP27 acts as a stress-responsive chaperone that facilitates the recruitment of autophagy receptors to ubiquitinated lysosomes [33]. Our data placing p38/MK2 upstream of this response in ANXA11 proteinopathy highlight a potential druggable node. While chronic p38 activation is often viewed as detrimental in neuroinflammation, our findings suggest its acute activation serves a vital homeostatic function in sensing membrane damage. This duality implies that therapeutic strategies must be carefully calibrated to enhance the cytoprotective MK2-HSP27 branch without triggering deleterious inflammatory cascades.

The most significant translational insight from our study is the role of lysophagy in halting the cytoplasmic invasion and seeding activity of ANXA11. The prevailing model of disease progression posits that pathological aggregates must gain access to the cytosolic pool of native proteins to template their misfolding. Our results from the *RB1CC1*-KD and pharmacological inhibition models demonstrate that lysosomes effectively sequester the internalized fibrils. Efficient lysophagy ensures engulfment and degradation of ruptured lysosomes, neutralizing the seeds. Conversely, when lysophagy is compromised, the ruptured lysosomes release seeds into the cytoplasm, where they nucleate the aggregation of endogenous ANXA11. This model is particularly relevant to the pathogenesis of FTLD/ALS, where the lysosomal clearance capacity is dually challenged by aging and genetic susceptibility. First, as the most significant risk factor for these neurodegenerative disorders, aging is intrinsically accompanied by a progressive decline in lysosomal acidification, proteolytic enzyme activity, and overall autophagic flux [46]. Second, it is now well established that a wide array of genes causing FTLD/ALS encode proteins that localize to lysosomes or directly modulate endolysosomal function. Indeed, pathogenic mutations in genes including *C9orf72*, *CHMP2B, GRN*, *TBK1*, *VCP*, *TMEM106B* and *SQSTM1/p62* severely compromise lysosomal homeostasis and autophagy, establishing lysosome dysfunction as a central, convergent pathogenic mechanism across the FTLD/ALS spectrum [47–49]. Our findings suggest that the rigid, rupture-inducing nature of ANXA11 fibrils might act synergistically with these pre-existing, age-dependent or genetically driven lysosomal deficits. In this double-hit scenario, a compromised lysosomal system may fail to mount an effective repair or lysophagic response (such as the p38/MK2/HSP27 axis identified here), thereby accelerating the cytoplasmic escape of amyloid seeds and the progressive spreading of pathology across the neural network.

Our study also underscores the value of using human-relevant models. While 2D cell lines provided mechanistic clarity, our validation in 3D cerebral organoids confirms that these pathological events, uptake, LMP, and apoptosis, occur in a complex tissue environment resembling the human brain. The observation of profound lysosomal damage and cell death in organoids treated with ANXA11 PFFs bridges the gap between biochemical assays and patient pathology.

## Conclusion

In conclusion, our study suggests that lysosomal integrity dictates the fate of neurons exposed to ANXA11 aggregates. We define the p38/MK2/HSP27 axis as a key sensor of ANXA11-induced lysosomal stress and identify lysophagy as an essential barrier against the cell-to-cell propagation of amyloid seeds. Therapeutic strategies capable of boosting this lysosomal quality control pathway, or stabilizing lysosomal membranes, hold significant promise for modifying the course of ANXA11-associated ALS and FTD.

## Supplementary Information

Additional file 1. **Fig. S1.** Exogenous ANXA11 PFFs alone are insufficient to trigger significant aggregation of cytosolic ANXA11-GFP in HEK293T cells. **Fig. S2.** Internalized ANXA11 PFFs show minimal accumulation in early endosomes. **Fig. S3.** Spontaneous endocytosis of ANXA11 PFFs effectively triggers lysosomal membrane permeabilization and lysophagy without lipid-mediated artifacts. **Fig. S4.** Validation of ANXA11 PFF-induced autophagic flux and RB1CC1 knockdown efficiency. **Fig. S5.** Validation of LLOMe-induced lysosomal damage controls and ESCRT knockdown efficiency. **Fig. S6. **Recruitment of ESCRT-III components CHMP2A and CHMP2B to ANXA11 PFF-positive lysosomes. **Fig. S7.** ANXA11 interacts with ESCRT-III components, and ESCRT deficiency exacerbates lysosomal damage and lysophagic flux. **Fig. S8.** Morphological characterization of WT and D40G ANXA11 amyloid fibrils. **Fig. S9.** Transcriptomic profiling reveals D40G-specific dysregulation of lysosomal trafficking and calcium signaling pathways. **Fig. S10. **ANXA11 amyloid fibrils trigger a catastrophic secondary collapse of RNA granule and lysosomal axonal transport. **Fig. S11.** Overexpression of HSP27 protects against ANXA11 PFF-induced lysosomal damage and attenuates intercellular propagation. **Fig. S12.** Generation and characterization of human iPSC-derived neurons. **Fig. S13. **ANXA11 D40G fibrils induce severe lysosomal pathology in cerebral organoids. **Fig. S14.** ANXA11 D40G fibrils trigger severe apoptotic cell death in human cerebral organoids. **Table S1.** The main primary antibodies used in this study.

Additional file 2. Original blots.

## Data Availability

All data supporting the findings of this study are available within the paper and its Supplementary Information.

## Abbreviations

ACTR10: Actin-related protein 10
ALS: Amyotrophic lateral sclerosis
ANXA11: Annexin A11
FTLD: Frontotemporal lobar degeneration
GAL3: Galectin-3
HSP27: Heat shock protein 27
iPSC: Induced pluripotent stem cell
LAMP1: Lysosomal-associated membrane protein 1
LC3: Microtubule-associated protein 1A/1B-light chain 3
LLOMe: L-leucyl-L-leucine methyl ester
LMP: Lysosomal membrane permeabilization
MAPK: Mitogen-activated protein kinase
MK2: MAPK-activated protein kinase 2
PFFs: Pre-formed fibrils
ROS: Reactive oxygen species
TEM: Transmission electron microscopy
TUNEL: Terminal deoxynucleotidyl transferase dUTP nick end labeling
WT: Wild-type
