# Supplementary material for: Lysophagy protects against ANXA11 amyloid fibril toxicity and propagation in FTLD
Source: Transl Neurodegener. 2026 Jun 28;15:29. doi: 10.1186/s40035-026-00561-5 (PMC13310443; doi:10.1186/s40035-026-00561-5)
Supplement: Supplementary file 1 — Additional file 1. Fig. S1. Exogenous ANXA11 PFFs alone are insufficient to trigger significant aggregation of cytosolic ANXA11-GFP in HEK293T cells. Fig. S2. Internalized ANXA11 PFFs show minimal accumulation in early endosomes. Fig. S3. Spontaneous endocytosis of ANXA11 PFFs effectively triggers lysosomal membrane permeabilization and lysophagy without lipid-mediated artifacts. Fig. S4. Validation of ANXA11 PFF-induced autophagic flux and RB1CC1 knockdown efficiency. Fig. S5. Validation of LLOMe-induced lysosomal damage controls and ESCRT knockdown efficiency. Fig. S6. Recruitment of ESCRT-III components CHMP2A and CHMP2B to ANXA11 PFF-positive lysosomes. Fig. S7. ANXA11 interacts with ESCRT-III components, and ESCRT deficiency exacerbates lysosomal damage and lysophagic flux. Fig. S8. Morphological characterization of WT and D40G ANXA11 amyloid fibrils. Fig. S9. Transcriptomic profiling reveals D40G-specific dysregulation of lysosomal trafficking and calcium signaling pathways. Fig. S10. ANXA11 amyloid fibrils trigger a catastrophic secondary collapse of RNA granule and lysosomal axonal transport. Fig. S11. Overexpression of HSP27 protects against ANXA11 PFF-induced lysosomal damage and attenuates intercellular propagation. Fig. S12. Generation and characterization of human iPSC-derived neurons. Fig. S13. ANXA11 D40G fibrils induce severe lysosomal pathology in cerebral organoids. Fig. S14. ANXA11 D40G fibrils trigger severe apoptotic cell death in human cerebral organoids. Table S1. The main primary antibodies used in this study. [file 40035_2026_561_MOESM1_ESM.docx]

**Supplementary information**

**
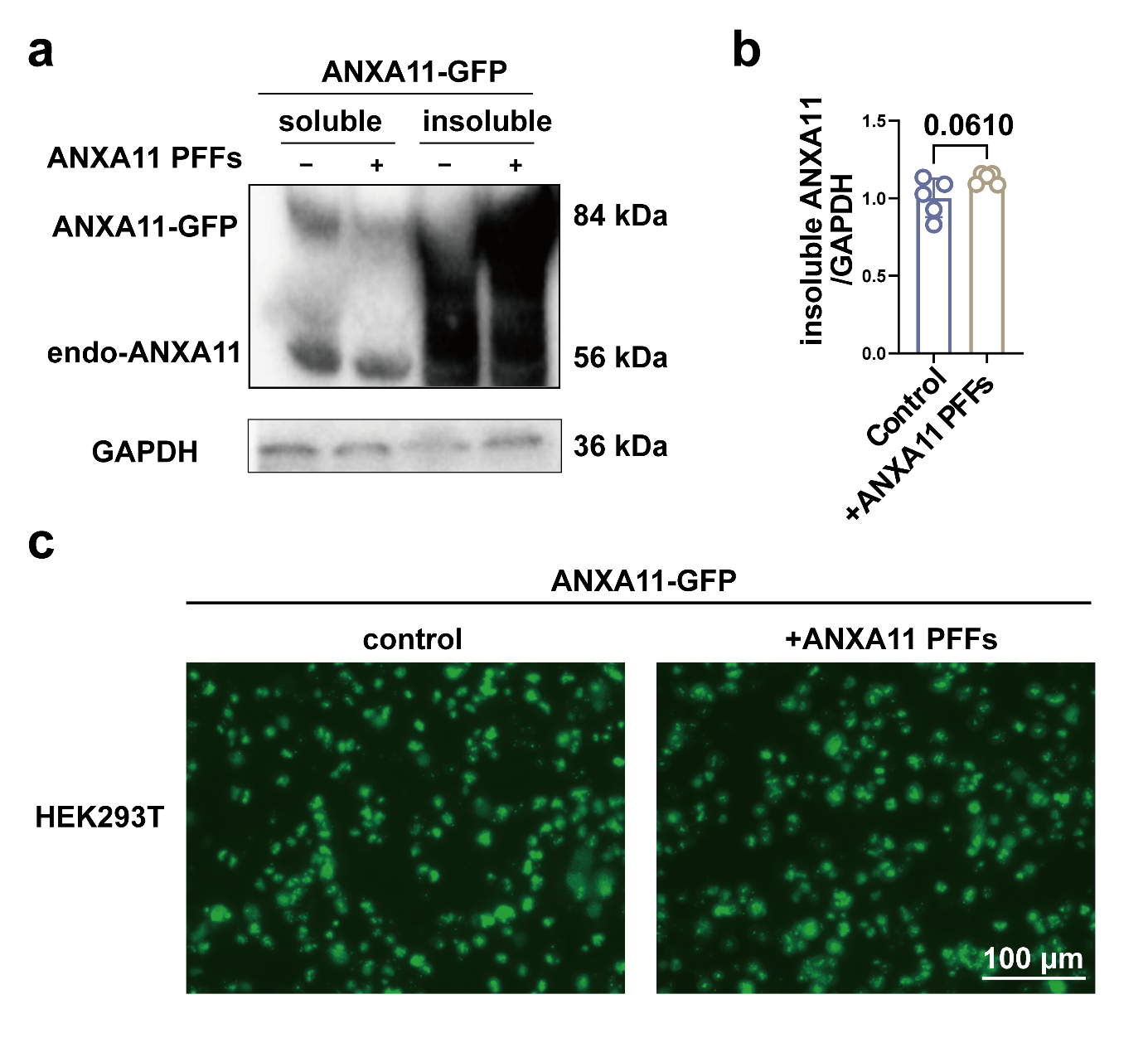
**

**Fig. S1. Exogenous ANXA11 PFFs alone are insufficient to trigger significant aggregation of cytosolic ANXA11-GFP in HEK293T cells. a** WB analysis of Triton X-100 soluble and insoluble fractions from HEK293T cells transiently expressing ANXA11-GFP. Cells were treated with vehicle or ANXA11 PFFs for 24 h. Membranes were probed for ANXA11 (detecting both the 84 kDa ANXA11-GFP fusion protein and the 56 kDa endogenous ANXA11) and GAPDH. **b** Densitometric quantification of insoluble ANXA11 protein levels normalized to GAPDH from **(a)**. The increase in the insoluble fraction upon PFF treatment did not reach statistical significance (*P*=0.0610). **c** Representative fluorescence images of HEK293T cells expressing ANXA11-GFP treated with control or ANXA11 PFFs. While occasional localized puncta may be observed, the overall intracellular distribution of ANXA11-GFP remains largely diffuse, with no statistically significant increase in the formation of massive, solid-like aggregates typically indicative of robust cytosolic seeding. Data are presented as mean ± SEM. Statistical significance was determined using Student’s t-test (**b**). Exact *P*-values are indicated in the corresponding graphs. Scale bars: 100 µm (**c**).


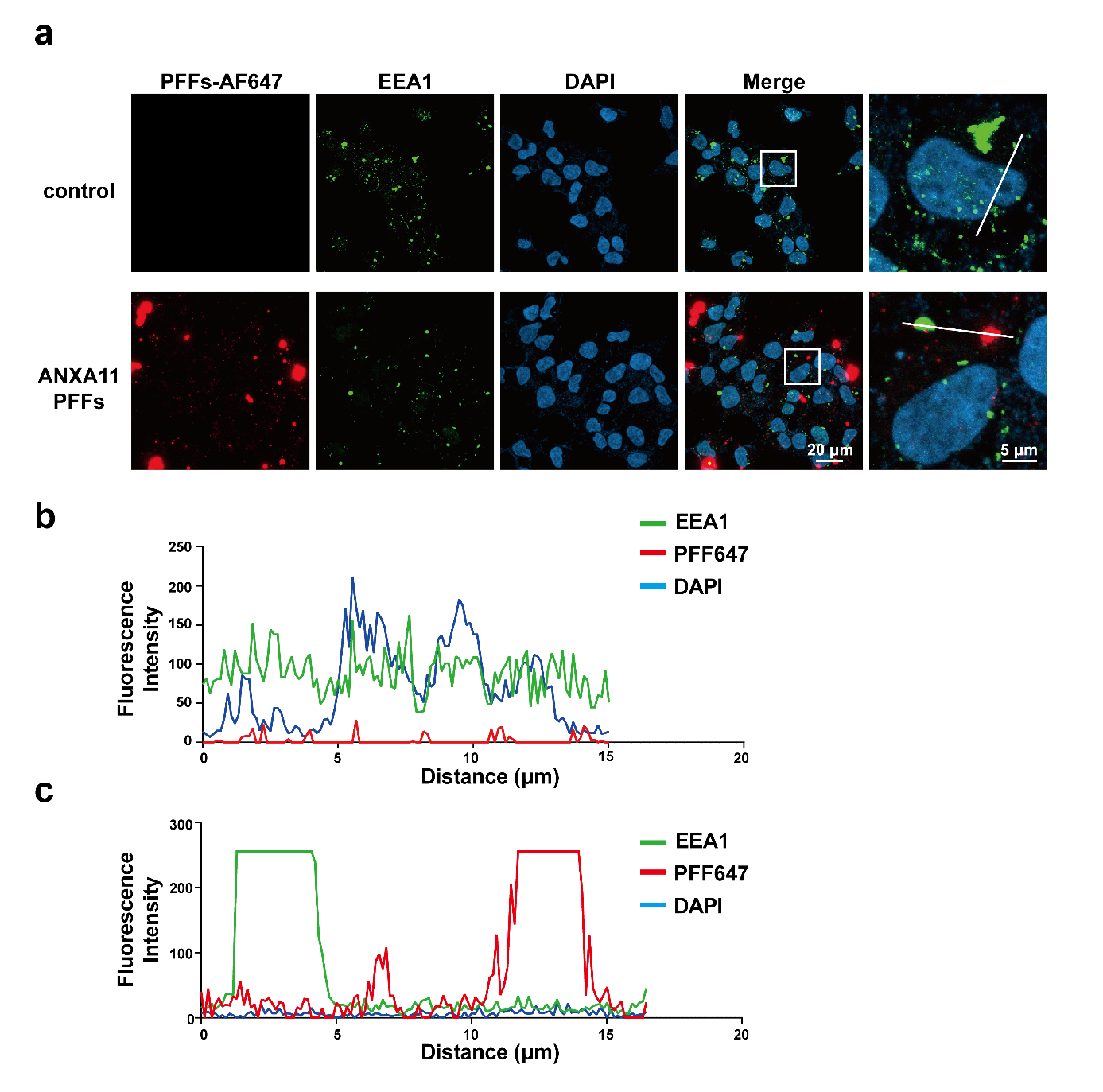


**Fig. S2. Internalized ANXA11 PFFs show minimal accumulation in early endosomes. a** Representative confocal immunofluorescence images of SH-SY5Y cells treated with AF647-labeled ANXA11 PFFs (red) for 24 h. Cells were stained for the early endosome marker EEA1 (green) and nuclei (DAPI, blue). **b-c** Fluorescence intensity profile analysis along the white lines indicated in **(a)**. The non-overlapping peaks of red (PFFs) and green (EEA1) signals confirm that ANXA11 aggregates are not significantly retained in early endosomes, supporting their rapid transport to lysosomes. Scale bars: 20 µm (**a**).


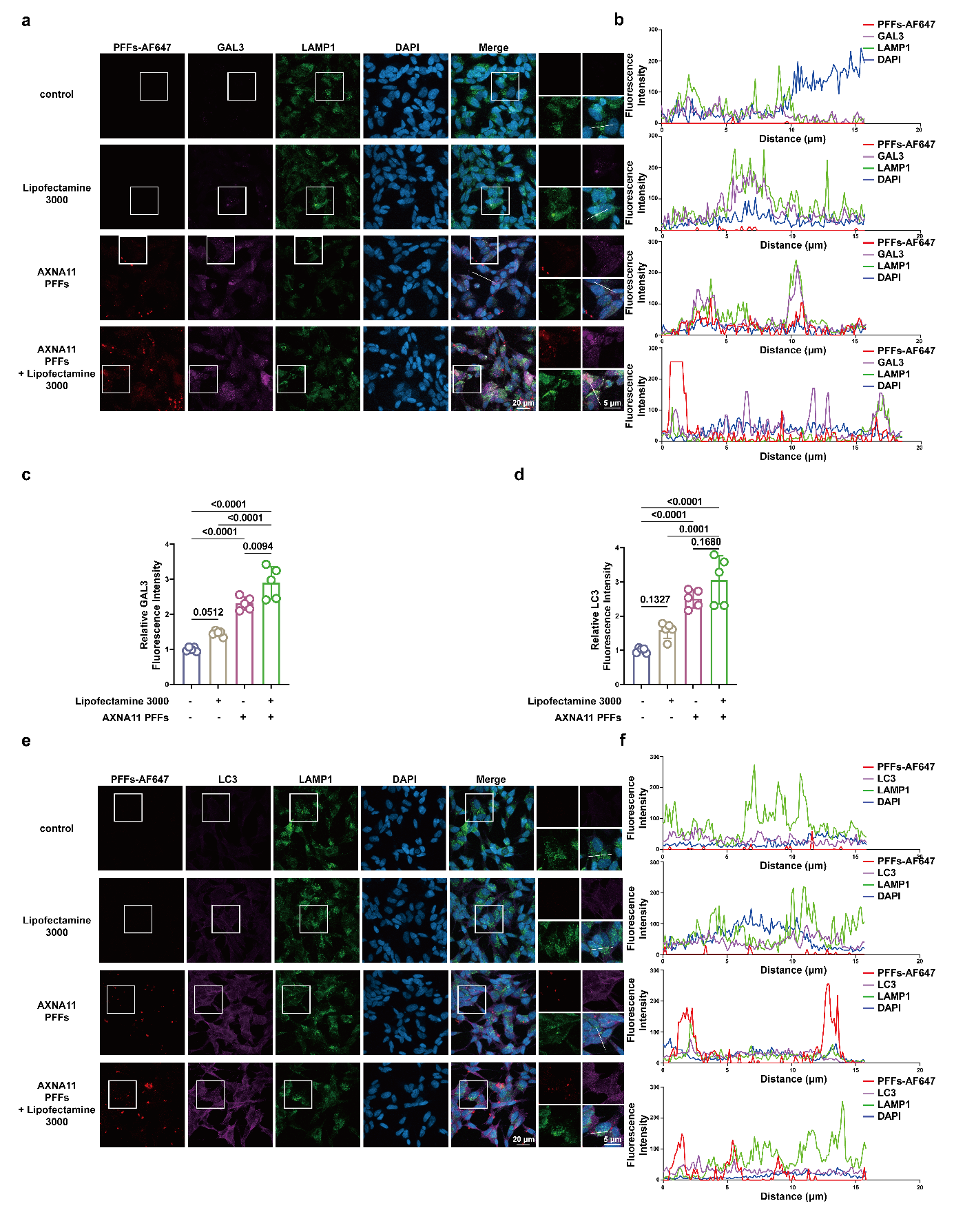


**Fig. S3. Spontaneous endocytosis of ANXA11 PFFs effectively triggers lysosomal membrane permeabilization and lysophagy without lipid-mediated artifacts. a** Representative confocal immunofluorescence images of SH-SY5Y cells subjected to four different treatments: vehicle (control), Lipofectamine 3000 alone, ANXA11 PFFs-AF647 alone (spontaneous endocytosis), and ANXA11 PFFs delivered via Lipofectamine 3000. Cells were stained for the lysosomal rupture marker Galectin-3 (GAL3, purple) and LAMP1 (green). Internalized PFFs are shown in red, and nuclei are stained with DAPI (blue). **b** Fluorescence intensity profile analysis along the white lines indicated in **(a)**, illustrating the spatial colocalization of GAL3, LAMP1, and PFFs across the different treatment conditions. **c** Quantification of relative GAL3 fluorescence intensity from **(a)**. The data demonstrate that spontaneous uptake of ANXA11 PFFs significantly induces GAL3 recruitment compared to controls. Notably, treatment with Lipofectamine 3000 alone does not cause significant LMP, confirming the specificity of fibril-induced damage. **d** Quantification of relative LC3 fluorescence intensity from **(e)**, showing robust lysophagic activation following spontaneous PFF uptake, while Lipofectamine 3000 alone does not significantly trigger LC3 recruitment. **e** Representative confocal images evaluating the lysophagic response under the same four treatment conditions. Cells were stained for the autophagy marker LC3 (purple) and LAMP1 (green), demonstrating the recruitment of autophagy machinery to PFF-containing lysosomes. **f** Fluorescence intensity profile analysis along the white lines in **(e)**, confirming the specific colocalization of LC3 with PFF-positive lysosomes. Data are presented as mean ± SEM. Statistical significance was determined using one-way ANOVA followed by Tukey’s post-hoc test (**c, d**). Exact *P*-values are indicated in the corresponding graphs. Scale bars: 20 µm (**a, e**).

**
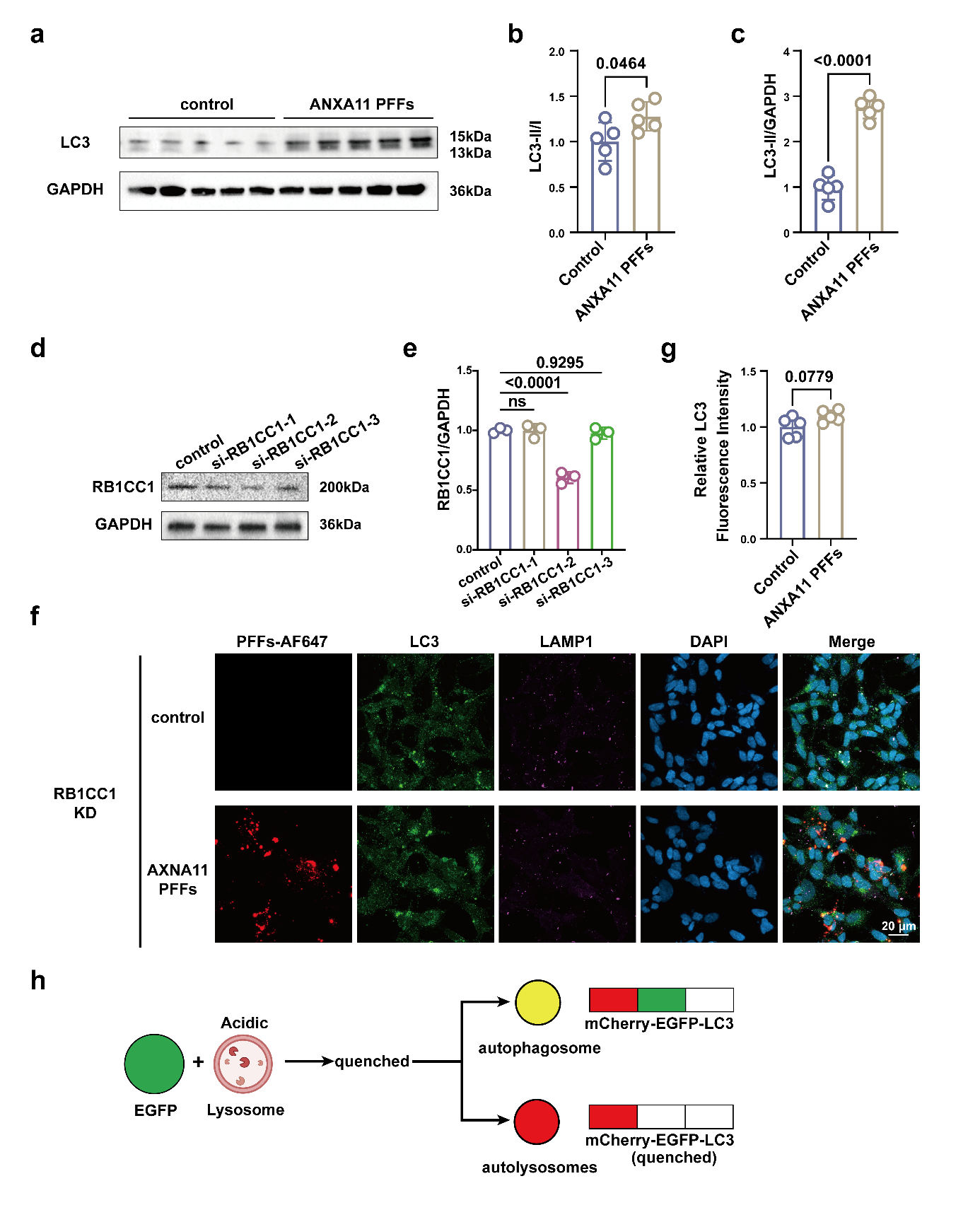
**

**Fig. S4. Validation of ANXA11 PFF-induced autophagic flux and RB1CC1 knockdown efficiency. a** Western blot analysis of LC3 conversion (LC3-I to LC3-II) in cells treated with control or ANXA11 PFFs, confirming autophagy induction.  **b-c** Densitometric quantification of the LC3-II/I ratio **(b)** and LC3-II/GAPDH ratio **(c)** from **(a)**.  **d** Western blot confirming the knockdown efficiency of RB1CC1 using three distinct siRNA sequences (si-RB1CC1-1, -2, -3). Sequence #2 showed the highest efficiency and was used for subsequent experiments. **e** Quantification of RB1CC1 protein levels normalized to GAPDH from **(d)**. **f** Representative immunofluorescence images of Control KD and RB1CC1 KD cells treated with ANXA11 PFFs. Staining for LC3 (green) and LAMP1 (purple) shows that RB1CC1 depletion abolishes the recruitment of LC3 to lysosomes containing PFFs (red). **g** Quantification of relative LC3 fluorescence intensity in PFF-treated cells. **h** Schematic illustration of the mCherry-EGFP-LC3 tandem reporter system used to monitor autophagic flux. In the acidic environment of autolysosomes, EGFP fluorescence is quenched, while mCherry fluorescence persists. Data are presented as mean ± SEM. Statistical significance was determined using Student’s t-test (**b, c, g**) or one-way ANOVA (**e**) followed by Tukey’s post-hoc test. Exact *P*-values are indicated in the corresponding graphs. Scale bars: 20 µm (**f**).

**
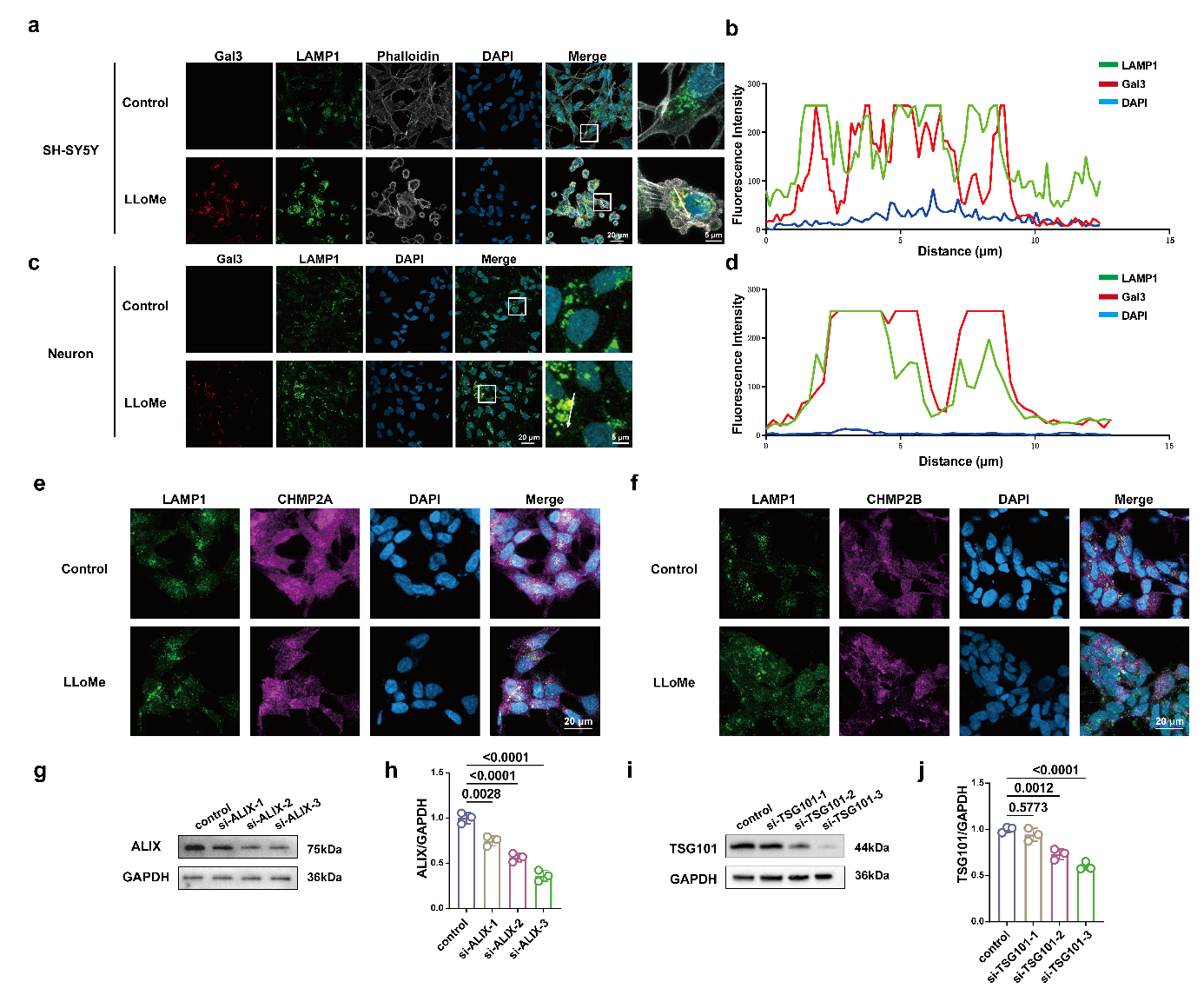
 Fig. S5. Validation of LLOMe-induced lysosomal damage controls and ESCRT knockdown efficiency. a** Representative images of SH-SY5Y cells treated with the lysosomotropic agent LLOMe (positive control). Staining for Gal3 (red) and LAMP1 (green) confirms lysosomal rupture. **b** Fluorescence intensity profile showing colocalization of Gal3 and LAMP1 in LLOMe-treated cells.  **c-d** Similar validation in iPSC-derived neurons **(c)** and corresponding intensity profile **(d)**, demonstrating robust Gal3 recruitment upon LLOMe treatment.  **e-f** Validation of ESCRT-III recruitment controls. LLOMe treatment triggers the rapid recruitment of CHMP2A **(e)** and CHMP2B **(f)** (purple) to LAMP1-positive lysosomes (green). **g** Western blot confirming the knockdown efficiency of ALIX using three distinct siRNA. **h** Quantification of ALIX protein levels from **(g)**. **i** Western blot confirming the knockdown efficiency of TSG101 using three distinct siRNA sequences. **j** Quantification of TSG101 protein levels from **(i)**. Data are presented as mean ± SEM. Statistical significance was determined using one-way ANOVA (**h, j**) followed by Tukey’s post-hoc test. Exact *P*-values are indicated in the corresponding graphs. Scale bars: 20 µm (**a, c, e, f**).


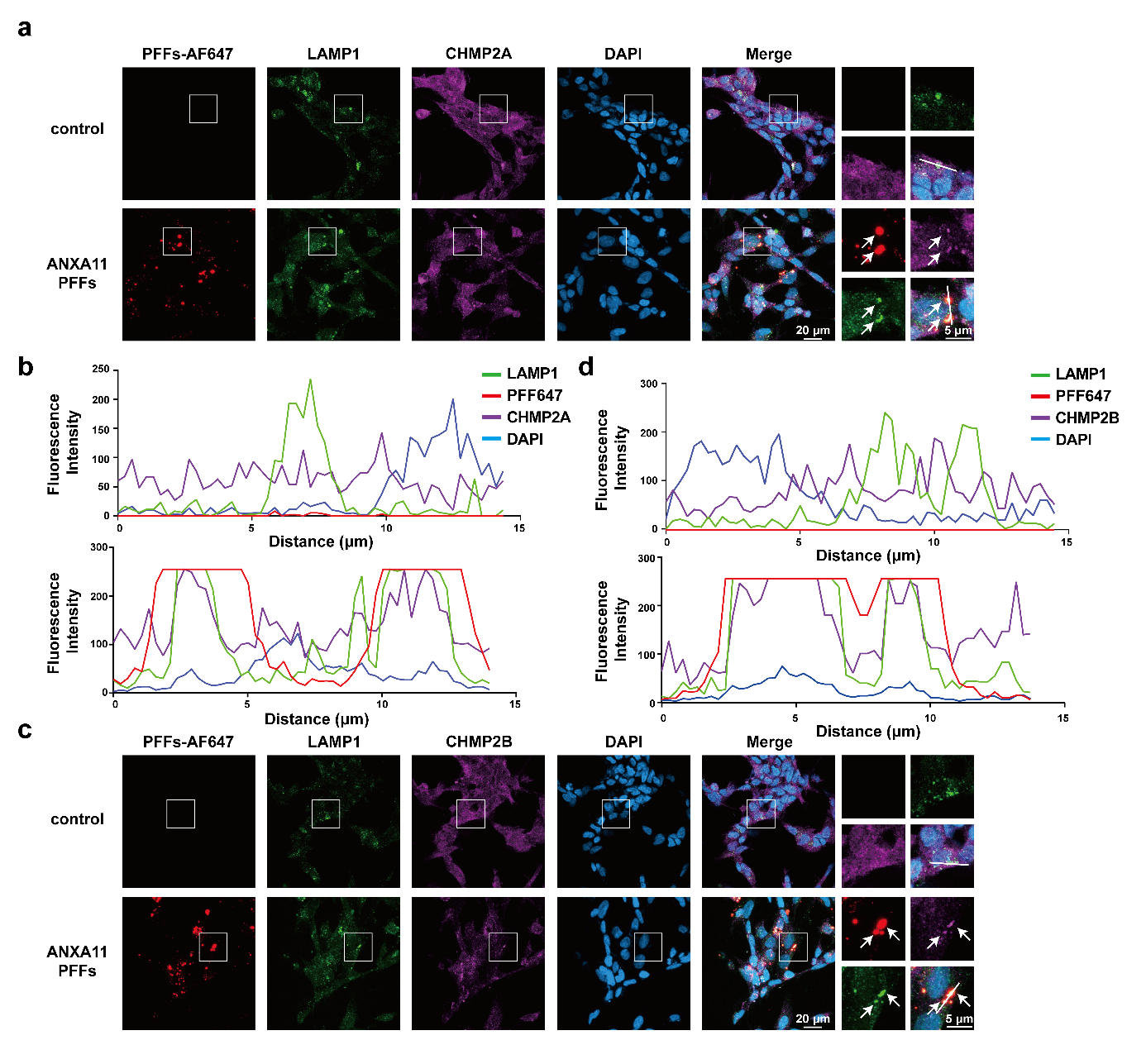


**Fig. S6. Recruitment of ESCRT-III components CHMP2A and CHMP2B to ANXA11 PFF-positive lysosomes. a** Representative confocal images showing the recruitment of the ESCRT-III component CHMP2A (purple) to LAMP1-positive lysosomes (green) containing ANXA11 PFFs-AF647 (red). Arrows indicate colocalization puncta. **b** Fluorescence intensity profile analysis along the white line in **(a)**, demonstrating the spatial overlap of ANXA11 PFFs, LAMP1, and CHMP2A. **c** Representative confocal images showing the recruitment of CHMP2B (purple) to lysosomes (green) containing ANXA11 PFFs-AF647 (red). **d** Fluorescence intensity profile analysis along the white line in **(c)**, confirming the colocalization of CHMP2B with PFF-positive lysosomes. Scale bars: 20 µm (**a, c**).

**
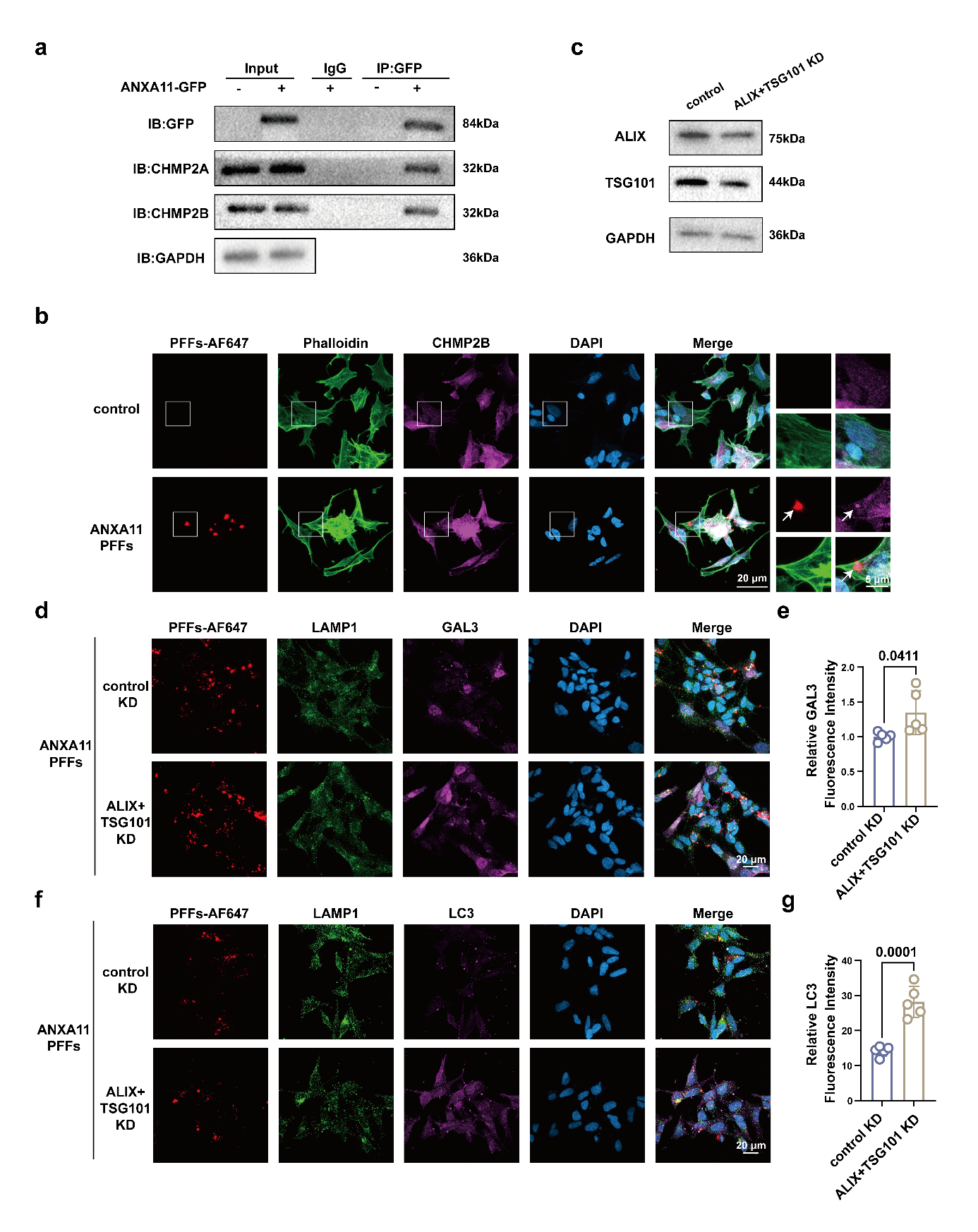
**

**Fig. S7. ANXA11 interacts with ESCRT-III components, and ESCRT deficiency exacerbates lysosomal damage and lysophagic flux. a** Co-immunoprecipitation (Co-IP) assay in HEK293T cells expressing ANXA11-GFP. Immunoblotting (IB) reveals that ANXA11-GFP physically interacts with endogenous CHMP2A and CHMP2B. **b** Immunofluorescence images showing the intracellular distribution of CHMP2B (purple) and ANXA11 PFFs (red) relative to the actin cytoskeleton (Phalloidin, green). Scale bars: 20 µm and 5 µm. **c** Western blot confirming the knockdown efficiency of ALIX and TSG101 in SH-SY5Y cells. GAPDH was used as a loading control. **d** Representative images of Galectin-3 (GAL3, purple) puncta in Control KD and ALIX+TSG101 KD cells treated with ANXA11 PFFs. Scale bars: 20 µm. **e** Quantification of relative GAL3 fluorescence intensity. The significant increase in GAL3 signal in KD cells indicates that impairment of ESCRT-mediated repair exacerbates lysosomal membrane rupture. **f** Confocal images of Control KD and ALIX+TSG101 KD cells treated with ANXA11 PFFs, stained for LC3 (purple) and LAMP1 (green). Scale bars: 20 µm. **g** Quantification of relative LC3 fluorescence intensity. Increased LC3 recruitment suggests a compensatory upregulation of lysophagy upon repair failure. Data are presented as mean ± SEM. Statistical significance was determined using Student’s t-test (**e, g**). Exact *P*-values are indicated in the corresponding graphs. Scale bars: 20 µm (**b, d, f**).**
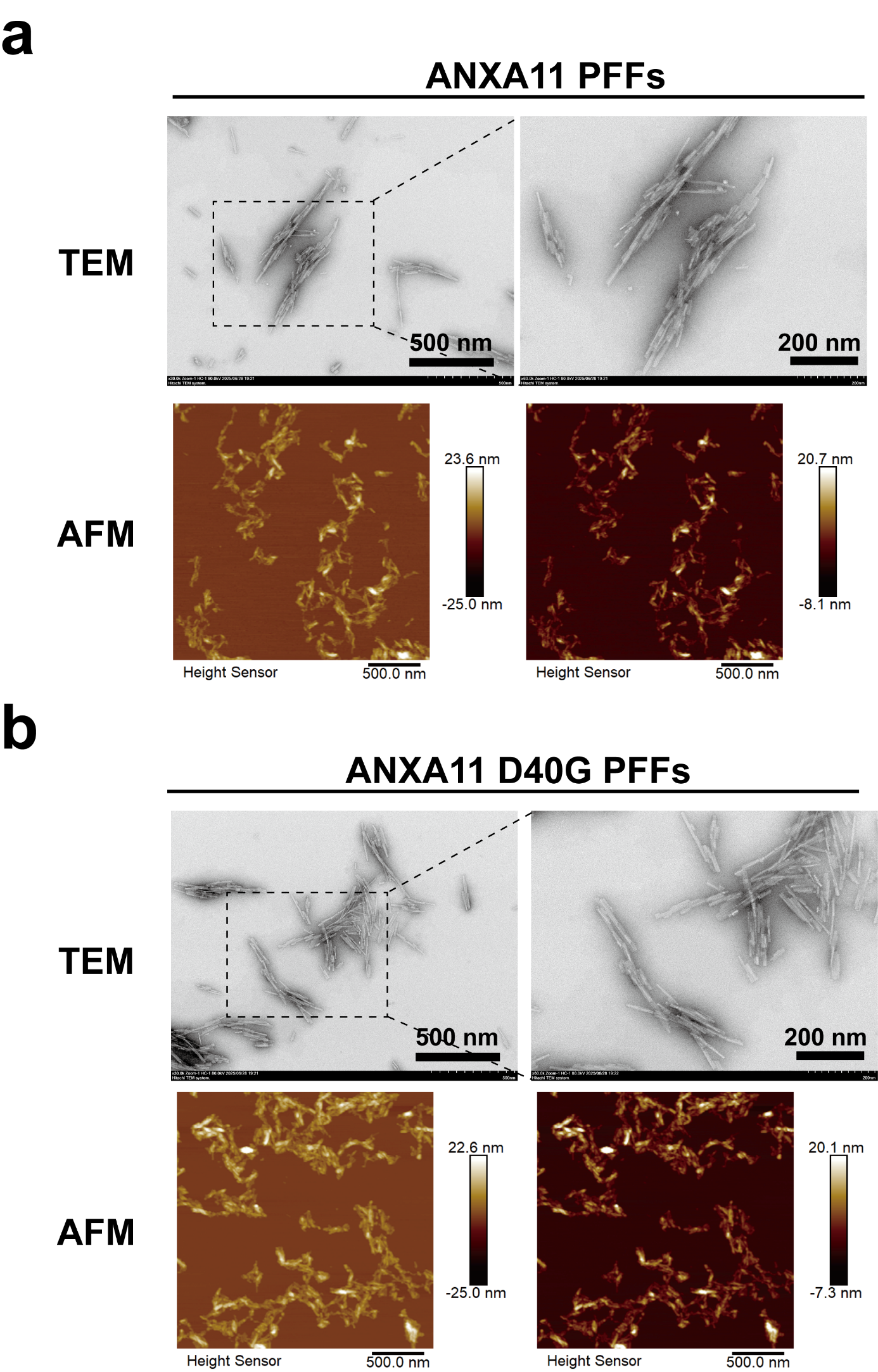
**

**Fig. S8. Morphological characterization of WT and D40G ANXA11 amyloid fibrils. a** Structural analysis of WT ANXA11 PFFs. Top: TEM images showing typical amyloid fibril morphology. Bottom: AFM images providing 3D topographical details of the fibrils. **b** Structural analysis of D40G mutant ANXA11 PFFs. Top: TEM images reveal that D40G fibrils form denser and more clustered networks compared to WT. Bottom: AFM images confirm the increased height and complexity of D40G fibril assemblies.

**
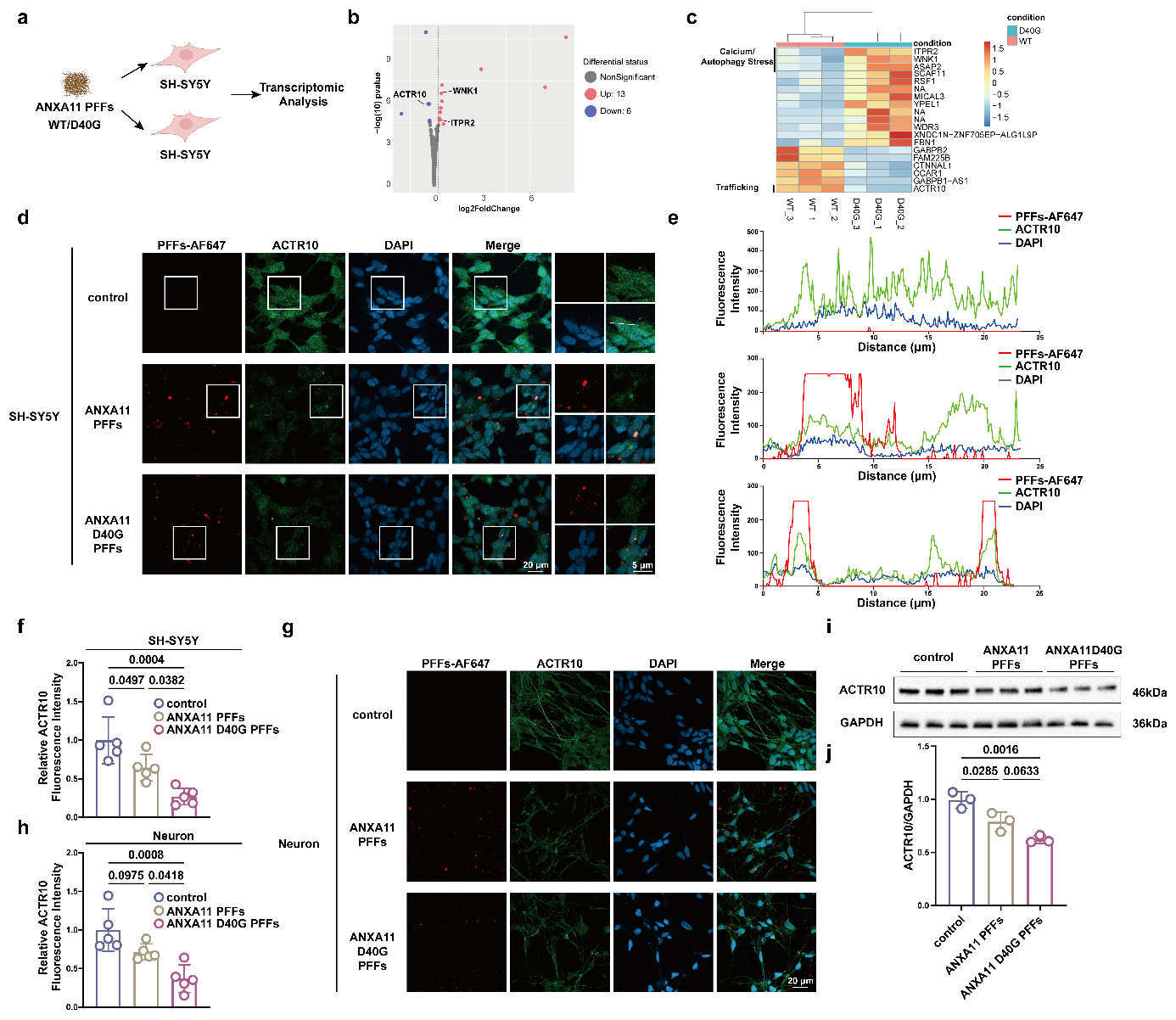
**

**Fig. S9. Transcriptomic profiling reveals D40G-specific dysregulation of lysosomal trafficking and calcium signaling pathways. a** Schematic illustration of the experimental workflow. SH-SY5Y cells were treated with WT or D40G mutant ANXA11 PFFs, followed by bulk RNA sequencing to identify mutation-specific transcriptional changes.  **b** Volcano plot displaying differentially expressed genes (DEGs) in D40G-treated cells compared to the WT group. The x-axis represents the log2 fold change, and the y-axis represents the -log10 P-value. Key genes involved in autophagy regulation (WNK1), calcium homeostasis (ITPR2), and retrograde axonal transport (ACTR10) are highlighted. **c** Heatmap of the identified DEGs. Genes were functionally grouped into "Calcium/Autophagy Stress" (upregulated, including ITPR2 and WNK1) and "Trafficking" (downregulated, including ACTR10). The color scale represents the Z-score of normalized gene expression, visualizing the distinct molecular signature where D40G fibrils simultaneously enhance stress signaling and impair transport machinery. **d** Representative confocal immunofluorescence images showing ACTR10 (green) expression in SH-SY5Y cells treated with vehicle (control), WT ANXA11 PFFs-AF647 (red), or D40G ANXA11 PFFs-AF647 (red) for 24 h. Nuclei were stained with DAPI (blue). **e** Fluorescence intensity profile analysis along the white lines drawn in **(d)**, illustrating the spatial distribution and the pronounced reduction of the ACTR10 signal in the D40G-treated group. **f** Quantification of relative ACTR10 fluorescence intensity in SH-SY5Y cells from **(d)**. **g** Representative confocal immunofluorescence images showing ACTR10 (green) expression in human iPSC-derived neurons treated with WT or D40G ANXA11 PFFs-AF647 (red). Scale bars: 20 µm. **h** Quantification of relative ACTR10 fluorescence intensity in iPSC-derived neurons from **(g)**. **i** Western blot analysis of total ACTR10 protein levels in cells treated with control, WT ANXA11 PFFs, or D40G ANXA11 PFFs. GAPDH served as a loading control. **j** Densitometric quantification of ACTR10 protein levels normalized to GAPDH from **(i)**. The results biochemically confirm the severe depletion of ACTR10 induced by the D40G mutation. Data are presented as mean ± SEM. Statistical significance was determined using one-way ANOVA (**f, h, j**) followed by Tukey’s post-hoc test. Exact *P*-values are indicated in the corresponding graphs. Scale bars: 20 µm (**d, g**).


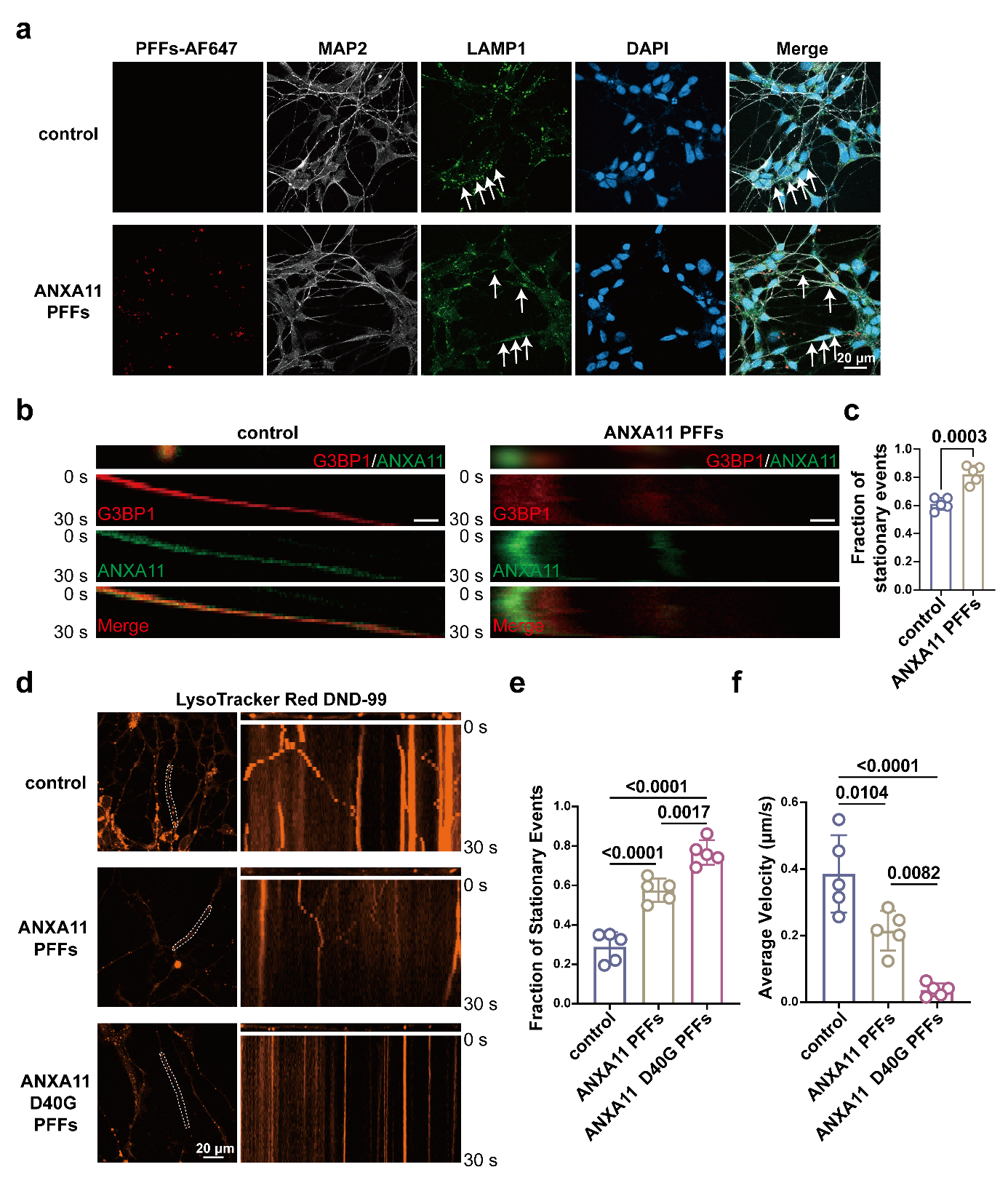


**Fig. S10. ANXA11 amyloid fibrils trigger a catastrophic secondary collapse of RNA granule and lysosomal axonal transport.** **a** Representative confocal immunofluorescence images of human iPSC-derived neurons treated with or without ANXA11 PFFs-AF647 (red). Neurons were immunostained for the dendritic/axonal marker MAP2 (white) and the lysosomal marker LAMP1 (green). Nuclei were counterstained with DAPI (blue). PFFs treatment induces abnormal clustering of lysosomes within the neurites. **b** Representative kymographs generated from dual-color live-cell imaging of human neurons tracking RNA granules (G3BP1, red) and ANXA11 (green) over a 3-minute period. In control neurons, diagonal trajectories indicate robust, processive co-transport. In ANXA11 PFF-treated neurons, vertical trajectories demonstrate a profound secondary collapse of RNA granule transport. **c** Quantification of the fraction of stationary events from the kymographs in **(b)**, confirming the functional paralysis of RNA transport. **d** Representative kymographs of LysoTracker Red DND-99-labeled lysosomes in iPSC-derived neurons treated with vehicle, WT ANXA11 PFFs, or D40G ANXA11 PFFs over a 3-minute period. The D40G mutant induces massive stationary lysosomal aggregates (vertical lines), severely abrogating the processive transport seen in controls. **e-f** Quantitative analysis of lysosomal trafficking from **(d)**, displaying the fraction of stationary events **(e)** and the average transport velocity **(f)**. The D40G mutation induces a significantly more severe lysosomal transport deficit compared to WT fibrils. Data are presented as mean ± SEM. Statistical significance was determined using Student’s t-test (**c**) or one-way ANOVA with Tukey’s post hoc test (**e, f**). Exact *P*-values are indicated in the corresponding graphs. Scale bars: 20 µm (**a, d**) and 5 µm (**b**).


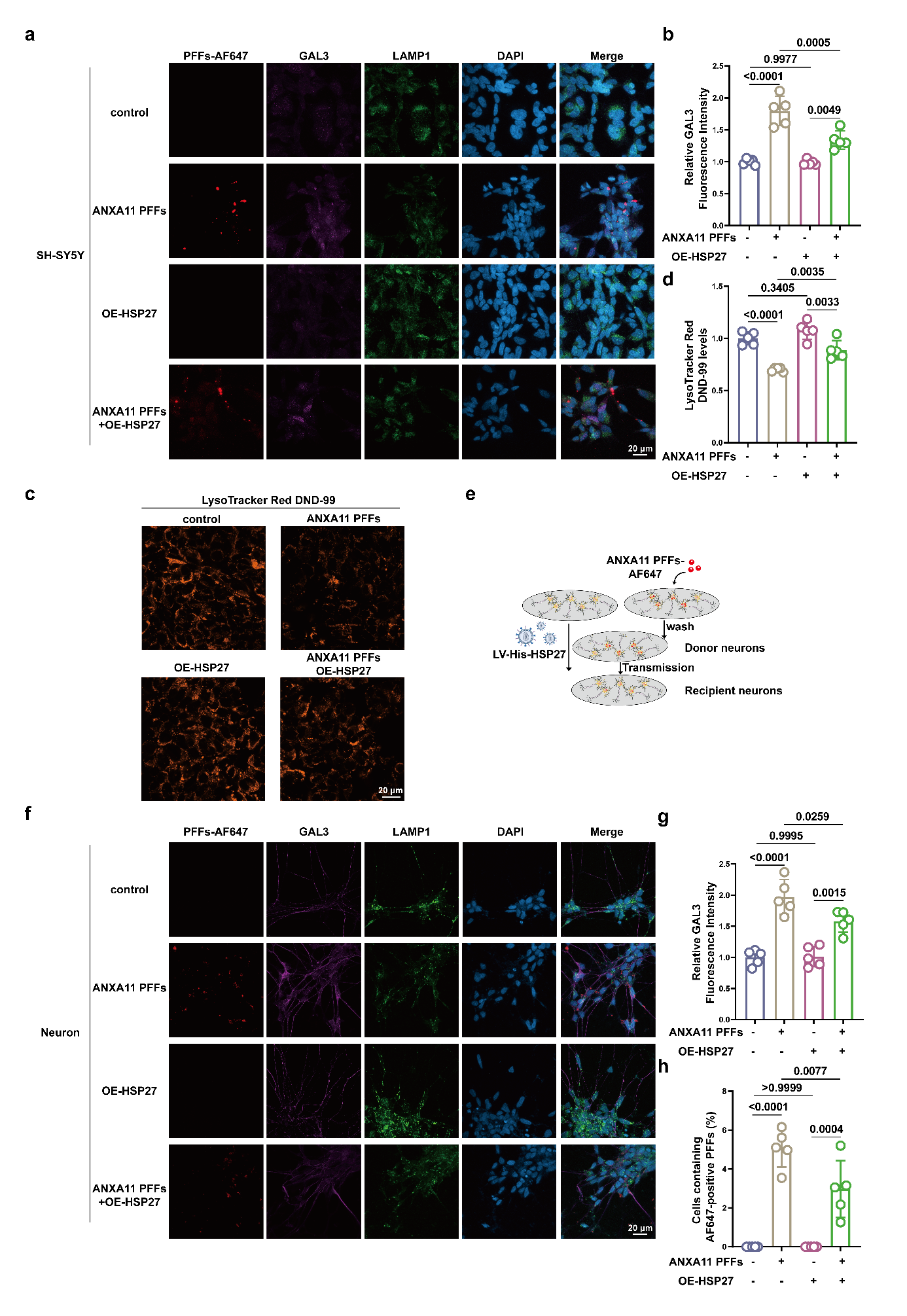


**Fig. S11. Overexpression of HSP27 protects against ANXA11 PFF-induced lysosomal damage and attenuates intercellular propagation. a** Representative confocal immunofluorescence images showing the effect of HSP27 overexpression (OE-HSP27) on lysosomal membrane permeabilization in SH-SY5Y cells. Cells were transfected with control or HSP27 overexpression lentiviral vector and treated with ANXA11 PFFs-AF647 (red). Lysosomes were labeled with LAMP1 (green), Galectin-3 (GAL3) is shown in purple, and nuclei were stained with DAPI (blue). **b** Quantification of relative GAL3 fluorescence intensity from **(a)**. The results demonstrate that HSP27 overexpression significantly reduces the accumulation of GAL3 puncta induced by ANXA11 PFFs. **c** Representative images of LysoTracker Red DND-99 staining in SH-SY5Y cells with or without OE-HSP27, assessing the recovery of lysosomal acidification following ANXA11 PFF exposure. **d** Quantification of LysoTracker Red fluorescence intensity from **(c)**, showing that OE-HSP27 rescues the loss of lysosomal acidity caused by PFF treatment. **e** Schematic illustration of the neuron-to-neuron transmission assay. Recipient iPSC-derived neurons were transduced with a lentiviral vector expressing His-HSP27 (LV-His-HSP27) prior to exposure to conditioned medium/lysate containing AF647-labeled ANXA11 PFFs from donor neurons. **f** Representative confocal images of recipient iPSC-derived neurons stained for LAMP1 (green) and GAL3 (purple). Internalized ANXA11 PFFs-AF647 are shown in red, and nuclei are stained with DAPI (blue). **g** Quantification of relative GAL3 fluorescence intensity in recipient neurons from **(f)**, confirming that OE-HSP27 alleviates lysosomal rupture in a neuronal model. **h** Quantification of the percentage of recipient cells containing AF647-positive ANXA11 inclusions. The data reveal that HSP27 overexpression in recipient neurons significantly reduces the accumulation and propagation of ANXA11 seeds. Data are presented as mean ± SEM. Statistical significance was determined using one-way ANOVA (**b, d, g, h**) followed by Tukey’s post-hoc test. Exact *P*-values are indicated in the corresponding graphs. Scale bars: 20 µm (**a, c, f**).

**
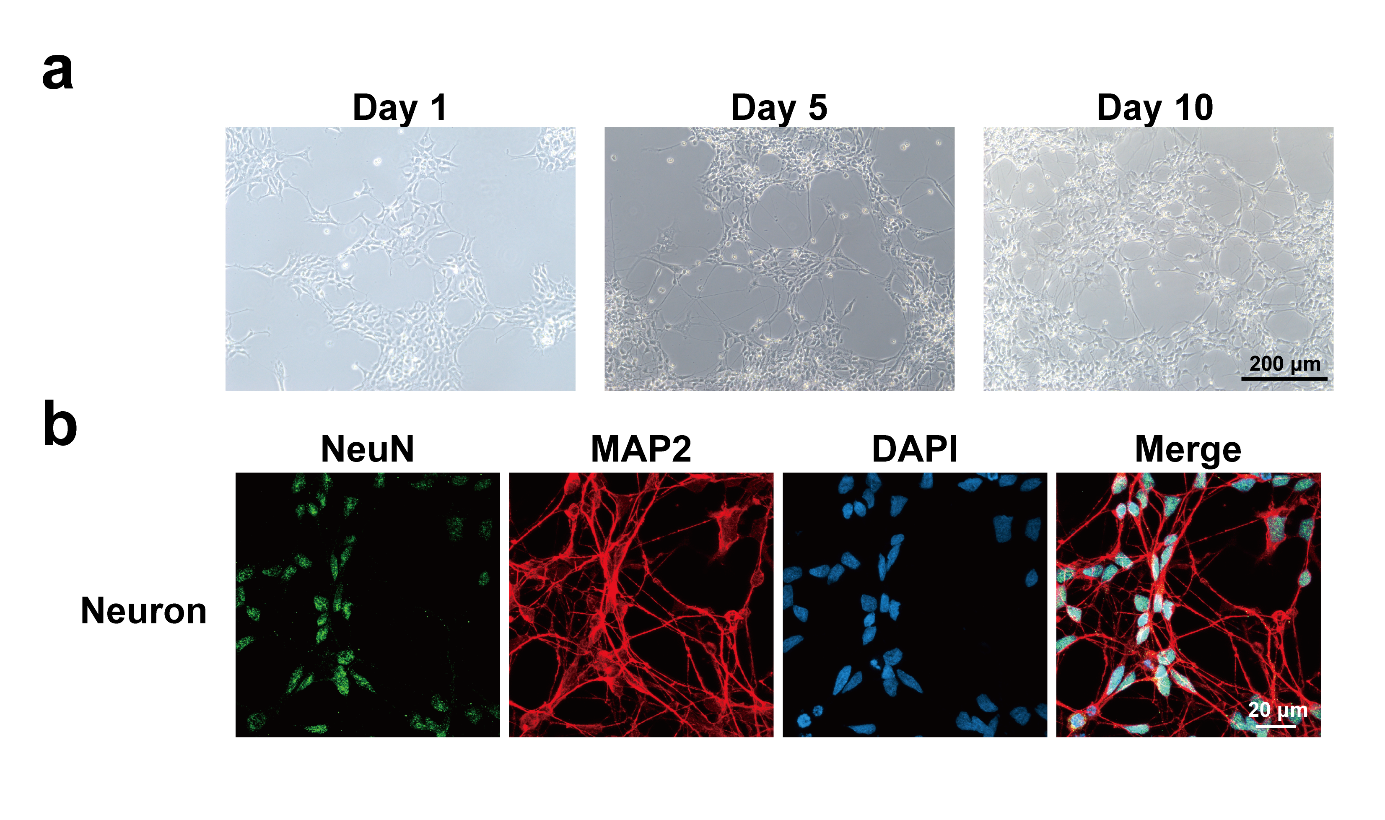
**

**Fig. S12. Generation and characterization of human iPSC-derived neurons. a** Representative brightfield images tracking the differentiation of human iPSCs into neurons at Day 1, Day 5, and Day 10. The cells exhibit progressive neurite outgrowth and morphological maturation. Scale bars: 200 µm.  **b** Immunofluorescence characterization of differentiated neurons. The cells stain positive for the neuronal nuclear marker NeuN (green) and the dendritic marker MAP2 (red), confirming successful neuronal differentiation. Nuclei are counterstained with DAPI (blue). Scale bars: 20 µm.

**
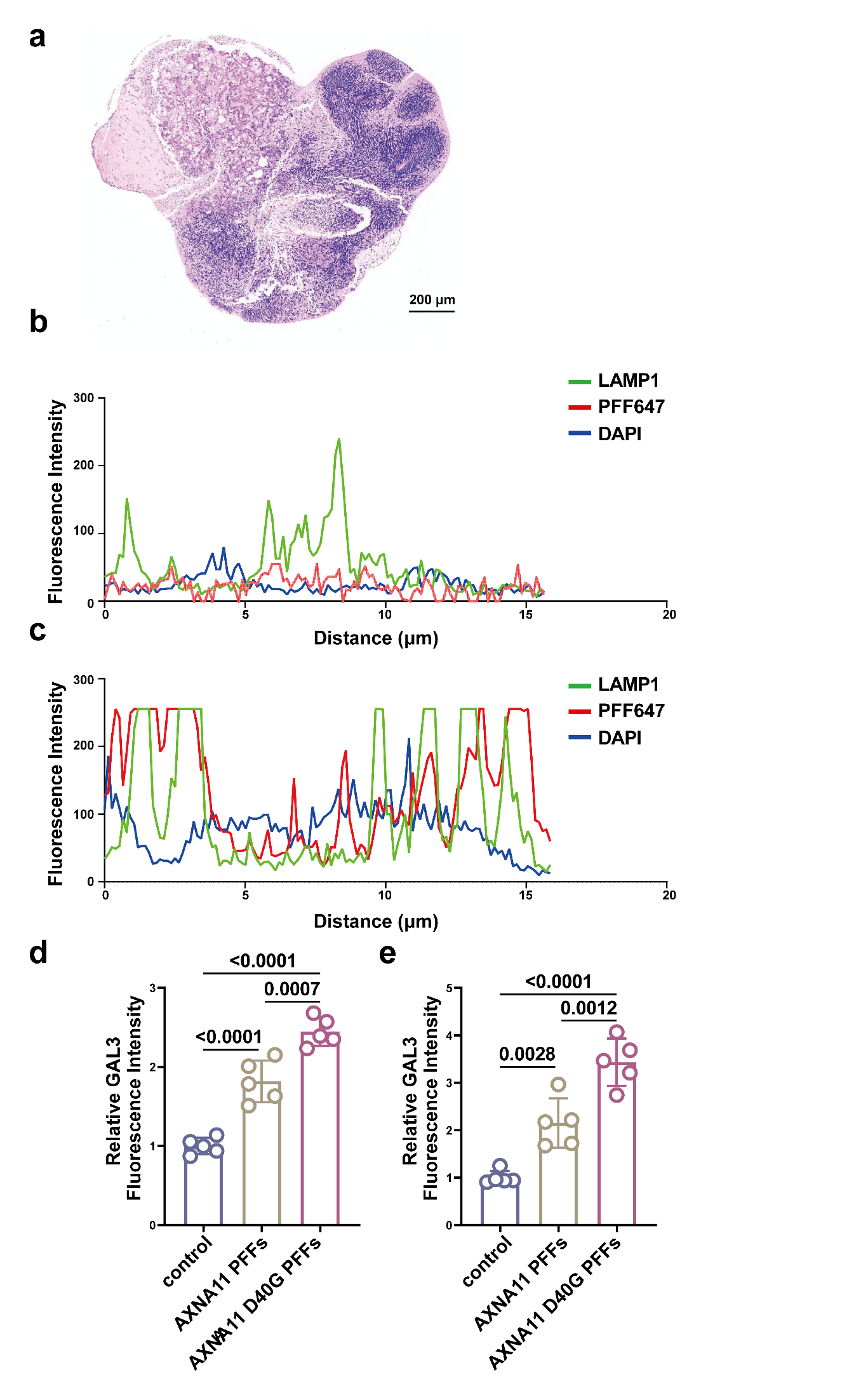
**

**Fig. S13. ANXA11 D40G fibrils induce severe lysosomal pathology in cerebral organoids. a** Representative hematoxylin and eosin (H&E) staining image of a Day 40 cerebral organoid section. The image displays the formation of complex tissue architecture with distinct neuroepithelial structures and laminar organization, confirming the structural maturity of the 3D model. Scale bar: 200 µm. **b-c** Fluorescence intensity profile analyses validating the colocalization of ANXA11 PFFs (red) with LAMP1-positive lysosomes (green) within the tissue of Day 40 cerebral organoids. **d** Quantification of relative GAL3 fluorescence intensity in organoids treated with WT vs. D40G ANXA11 PFFs. **e** Quantification of relative LC3 fluorescence intensity in organoids. The increased recruitment of LC3 in the D40G group indicates a hyperactive lysophagic response to severe membrane damage.


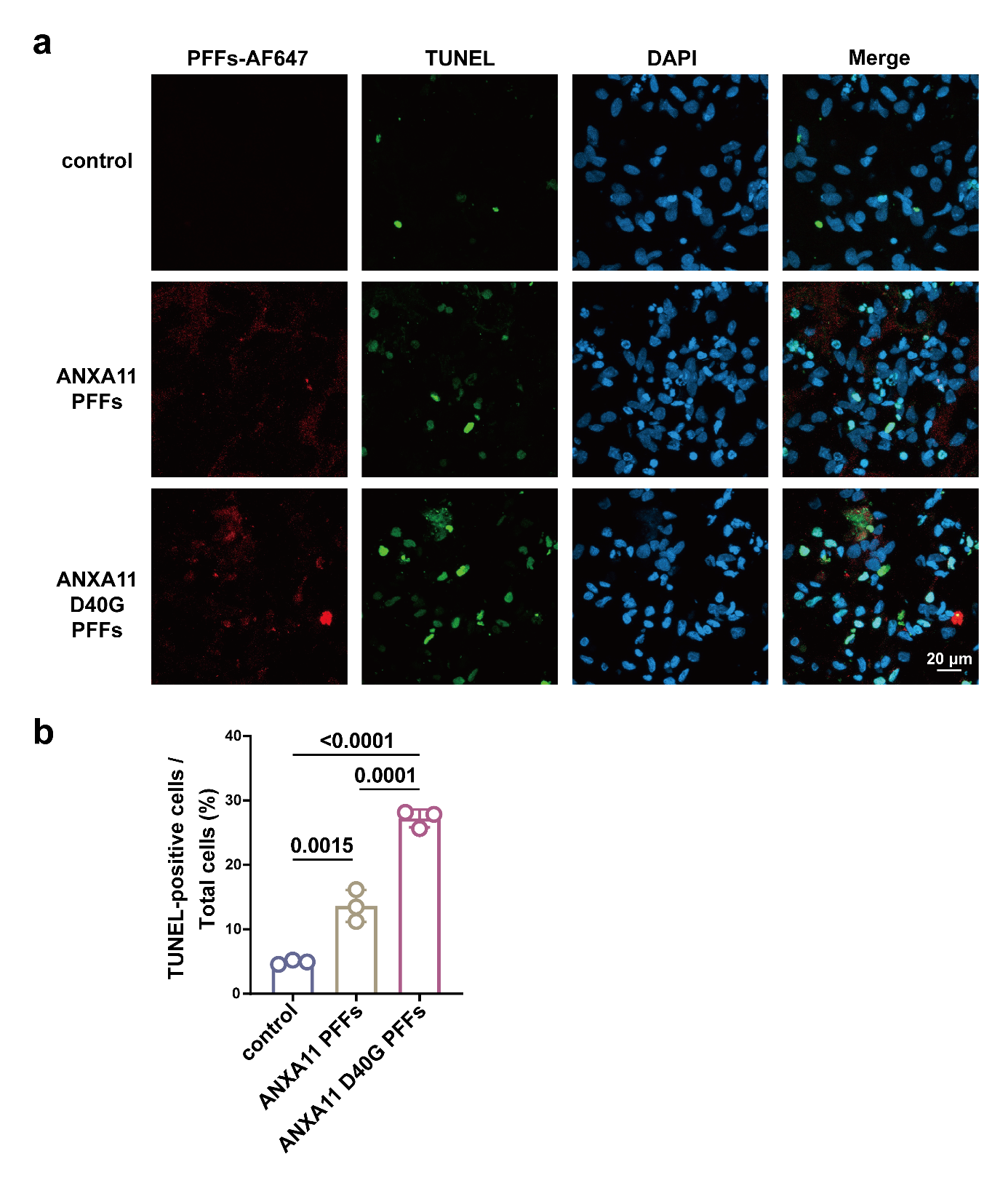


**Fig. S14. ANXA11 D40G fibrils trigger severe apoptotic cell death in human cerebral organoids. a** Representative confocal immunofluorescence images of TUNEL staining (green) in Day 40 human iPSC-derived cerebral organoids treated with WT or D40G ANXA11 PFFs-AF647 (red). Nuclei were counterstained with DAPI (blue). The widespread presence of TUNEL-positive nuclei, particularly in the D40G-treated group, indicates extensive DNA fragmentation and apoptosis within the organoid tissue. Scale bars: 20 µm. **b** Quantification of the apoptotic index, calculated as the percentage of TUNEL-positive cells relative to the total number of DAPI-stained cells.

**Table S1. The main primary antibodies used in this study.**

| **Primary antibodies** | **Company** | **Cat.** | **Dilution** |
| --- | --- | --- | --- |
| LAMP1 | BD Biosciences | 555798 | IF: 1:500 |
| EEA1 | Proteintech | 68065-1-Ig | IF: 1:1000 |
| CTSA | signalway antibody | 32893 | WB:1:1000 |
| CTSB | Cell Signaling Technology | 31718T | WB: 1:1000 |
| CTSD | Cell Signaling Technology | 74089T | WB: 1:1000 |
| Galectin-3 | Proteintech | 82024-1-RR | IF: 1:500 |
| LC3 | Proteintech | 14600-1-AP | IF: 1:500  WB: 1:1000 |
| RB1CC1 | Proteintech | 17250-1-AP | WB: 1:5000 |
| CHMP2A | Proteintech | 10477-1-AP | WB: 1:1000  IF: 1:200 |
| CHMP2B | Proteintech | 12527-1-AP | WB: 1:1000  IF: 1:200 |
| GAPDH | Proteintech | 10494-1-AP | WB: 1:10000 |
| GFP | Proteintech | 50430-2-AP | WB: 1:1000  IP:2μg for 3mg total protein  WB: 1:1000 |
| TSG101 | Proteintech | 28283-1-AP | WB: 1:5000 |
| ALIX | Proteintech | 12422-1-AP | WB: 1:5000 |
| p-HSP27 | Proteintech | 83332-3-RR | WB: 1:5000 |
| HSP27 | Proteintech | 18284-1-AP | WB: 1:10000  IF: 1:500 |
| p38 MAPK | Cell Signaling Technology | 9212S | WB: 1:1000 |
| p-p38 MAPK (T180/Y182) | abcam | ab4822 | WB: 1:250 |
| Phospho-MAPKAPK-2 (Thr334) | Cell Signaling Technology | 3007T | WB: 1:1000 |
| MAPKAPK2 | Proteintech | 13949-1-AP | WB: 1:1000 |
| TOM20 | Proteintech | 11802-1-AP | IF: 1:200 |
| Caspase-3 | Cell Signaling Technology | 9662S | WB: 1:1000 |
| Cleaved Caspase-3 (Asp175) | Cell Signaling Technology | 9661S | WB: 1:1000 |
| Oligomer A11 | Invitrogen | AHB0052 | Dot blot: 1:1000 |
| Amyloid Fibrils  (OC) | Merck | AB2286 | Dot blot: 1:1000 |
| MAP2 | Proteintech | 17490-1-AP | IF: 1:200  mIHC: 1:1000 |
| NeuN | AiFang biological | AFRM0088 | mIHC: 1:500 |
| PAX6 | AiFang biological | AFRM0226 | mIHC: 1:200 |
| TBR1 | AiFang biological | AFRM0110 | mIHC: 1:300 |
| TUJ1 | AiFang biological | AFRM0073 | mIHC: 1:500 |
| ANXA11 | Proteintech | 10479-2-AP | mIHC: 1:1000 |
| ACTR10 | Proteintech | 20101-1-AP | IF: 1:100  WB: 1:1000 |

IF: immunofluorescence; WB: western blot; IP: immunoprecipitation; mIHC: Multiplex immunohistochemical staining
