# Supplementary material for: Lysophagy protects against ANXA11 amyloid fibril toxicity and propagation in FTLD
Source: Transl Neurodegener. 2026 Jun 28;15:29. doi: 10.1186/s40035-026-00561-5 (PMC13310443; doi:10.1186/s40035-026-00561-5)
Supplement: Supplementary file 2 — Additional file 2. Original blots. [file 40035_2026_561_MOESM2_ESM.pdf]

Figure 1f

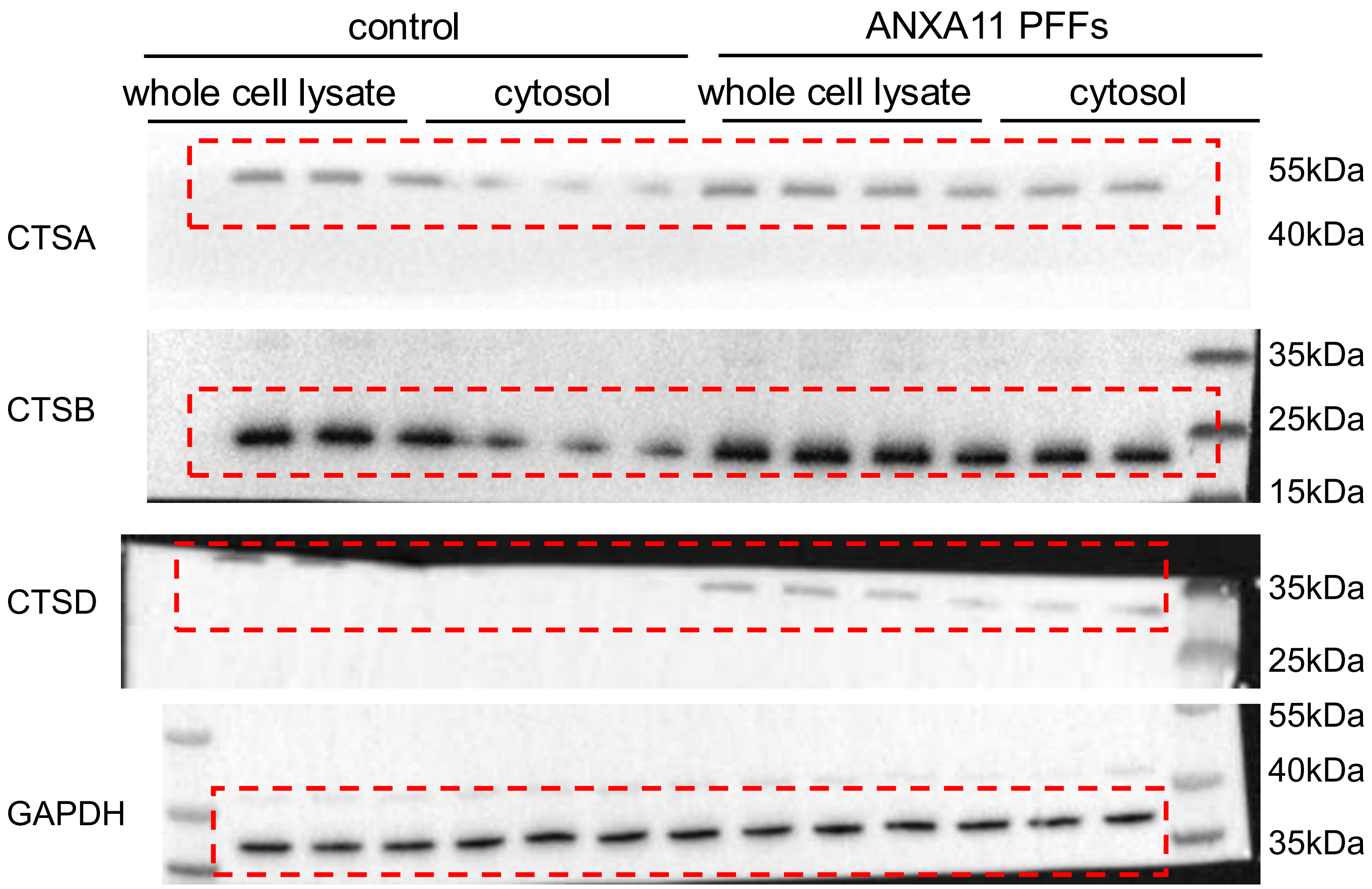
control
ANXA11 PFFs
whole cell lysate
cytosol
whole cell lysate
cytosol
CTSA
CTSB
CTSD
GAPDH
55kDa
40kDa
35kDa
25kDa
15kDa
35kDa
25kDa
55kDa
40kDa
35kDa

Figure 2a

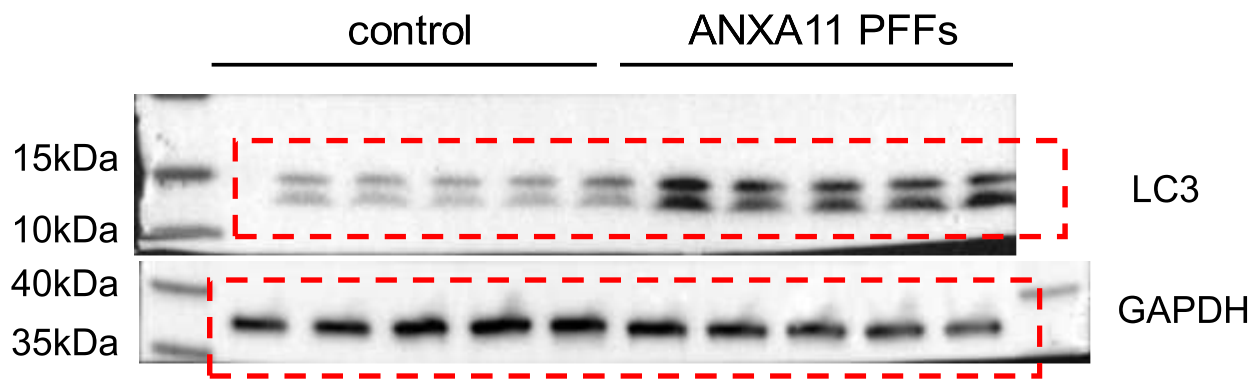

Figure 2g

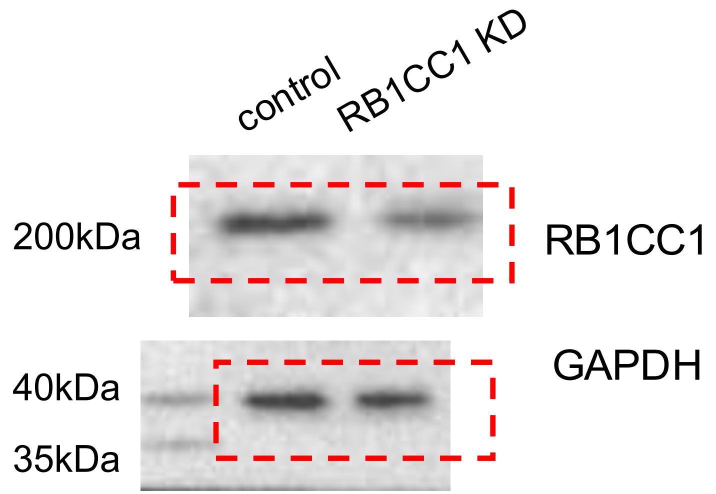

Figure 2h

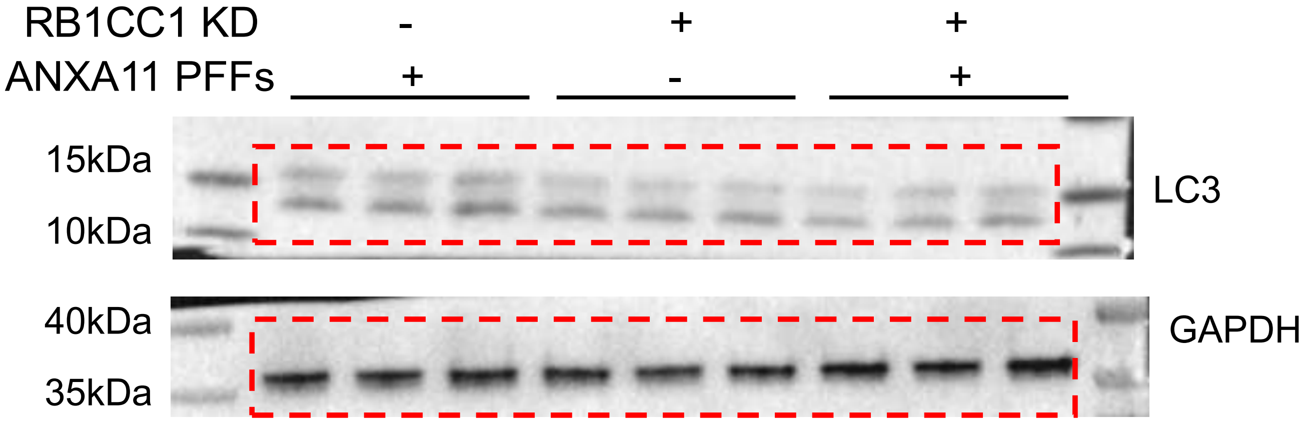

Figure 4a

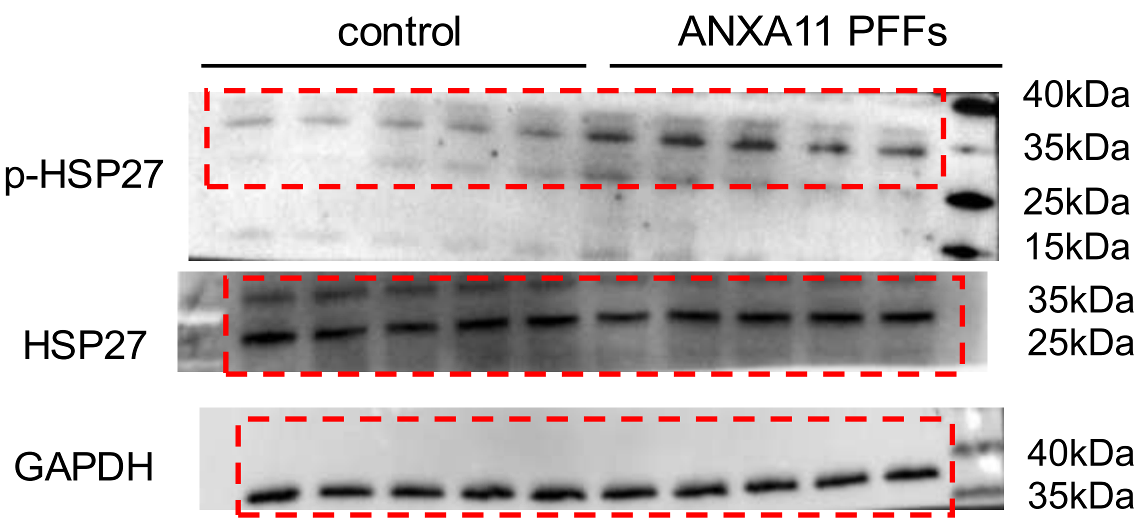

Figure 4m

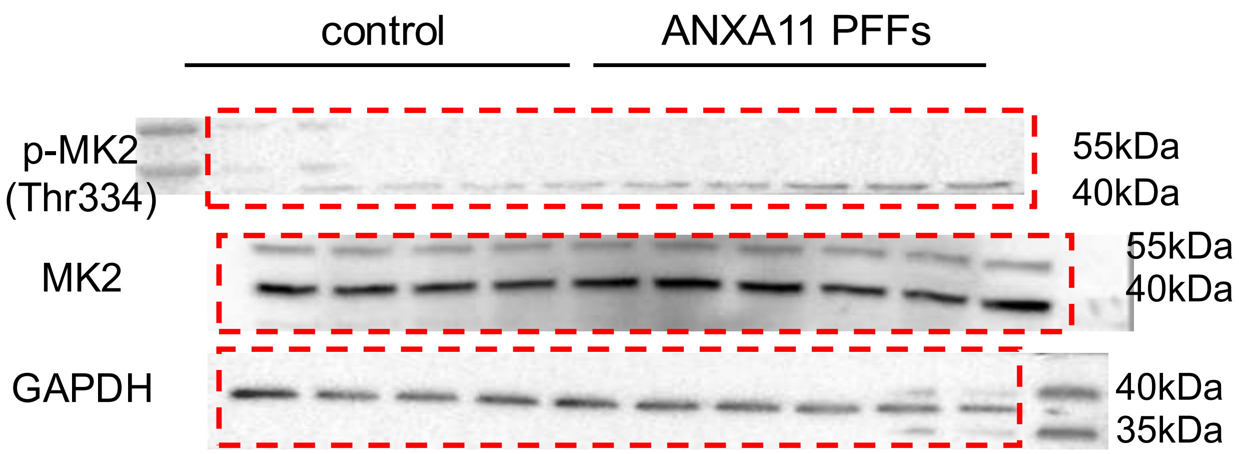

Figure 4e

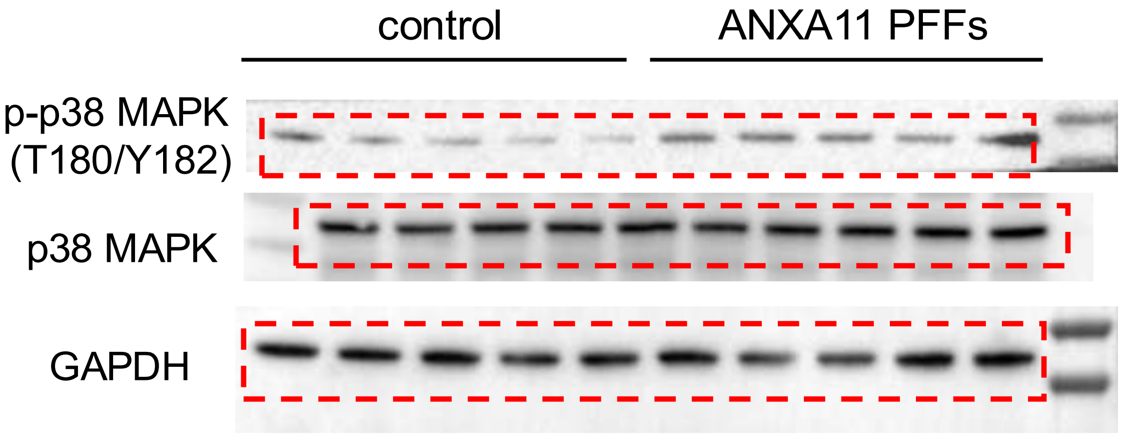

Figure 4h

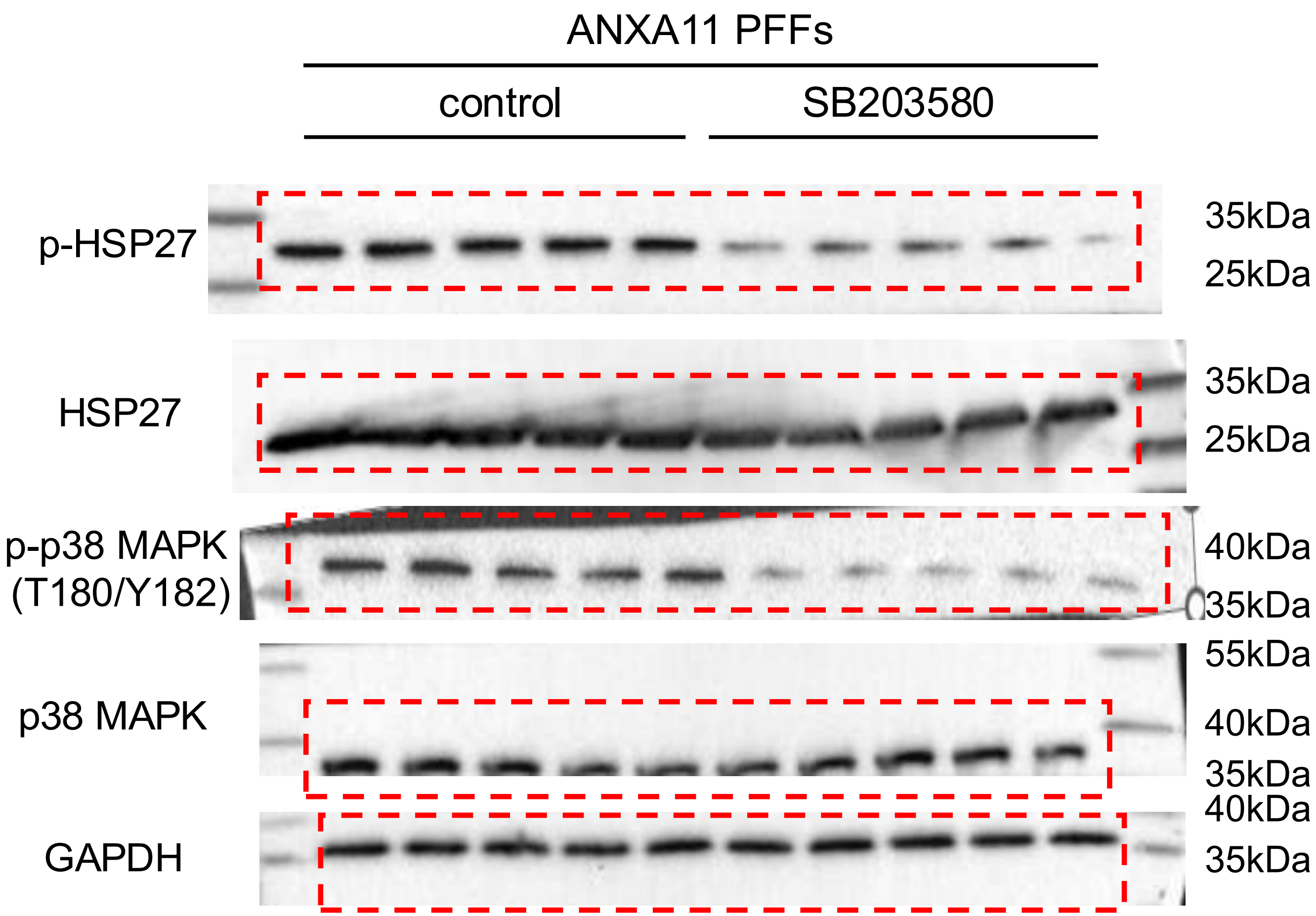

ANXA11 PFFs
control
SB203580
p-HSP27
35kDa
25kDa
HSP27
35kDa
25kDa
p-p38 MAPK
(T180/Y182)
40kDa
35kDa
p38 MAPK
55kDa
40kDa
35kDa
GAPDH
40kDa
35kDa

Figure 4p

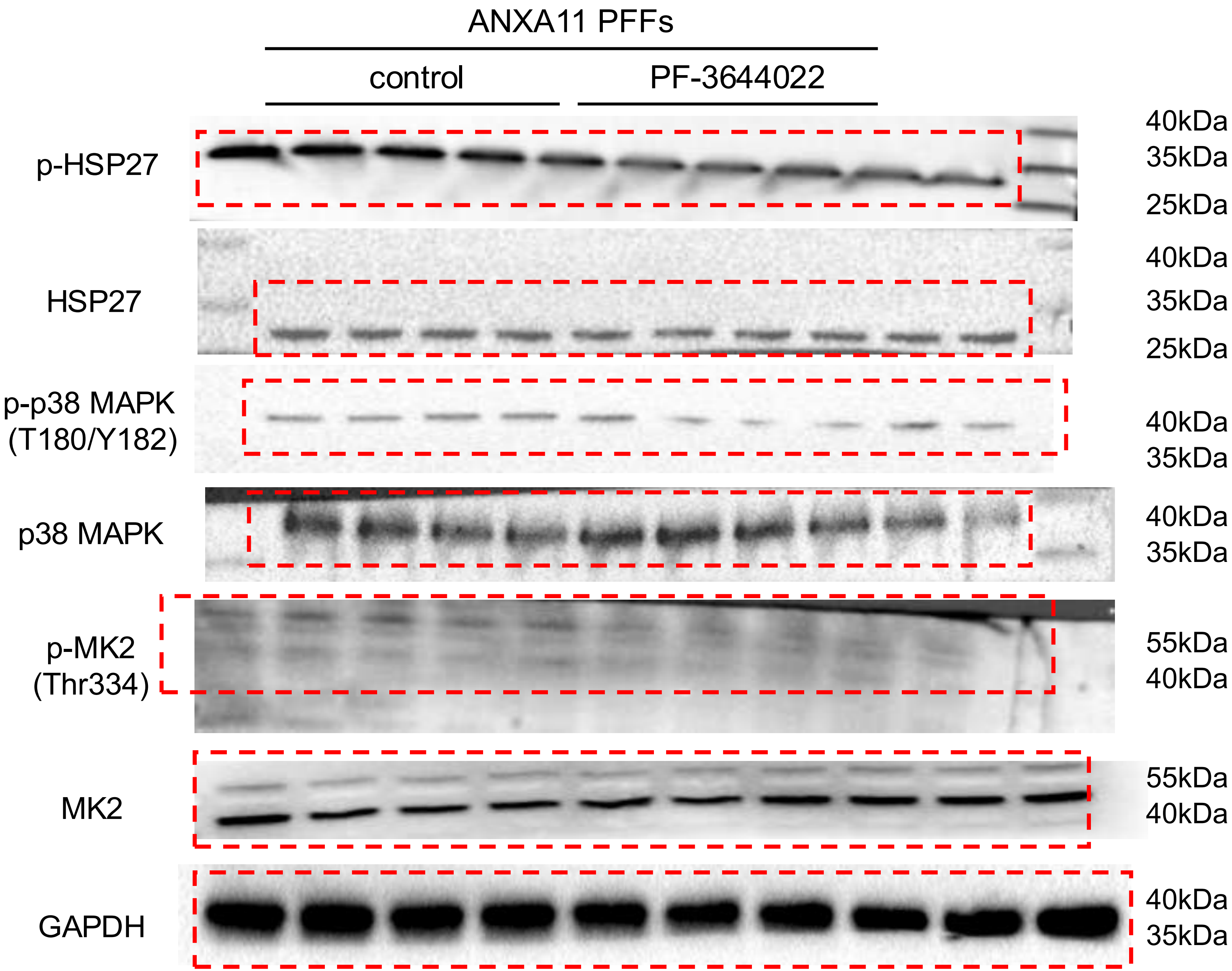
ANXA11 PFFs
control
PF-3644022
p-HSP27
HSP27
p-p38 MAPK
(T180/Y182)
p38 MAPK
p-MK2
(Thr334)
MK2
GAPDH
40kDa
35kDa
25kDa
40kDa
35kDa
25kDa
40kDa
35kDa
40kDa
35kDa
55kDa
40kDa
55kDa
40kDa
40kDa
35kDa

Figure 5c

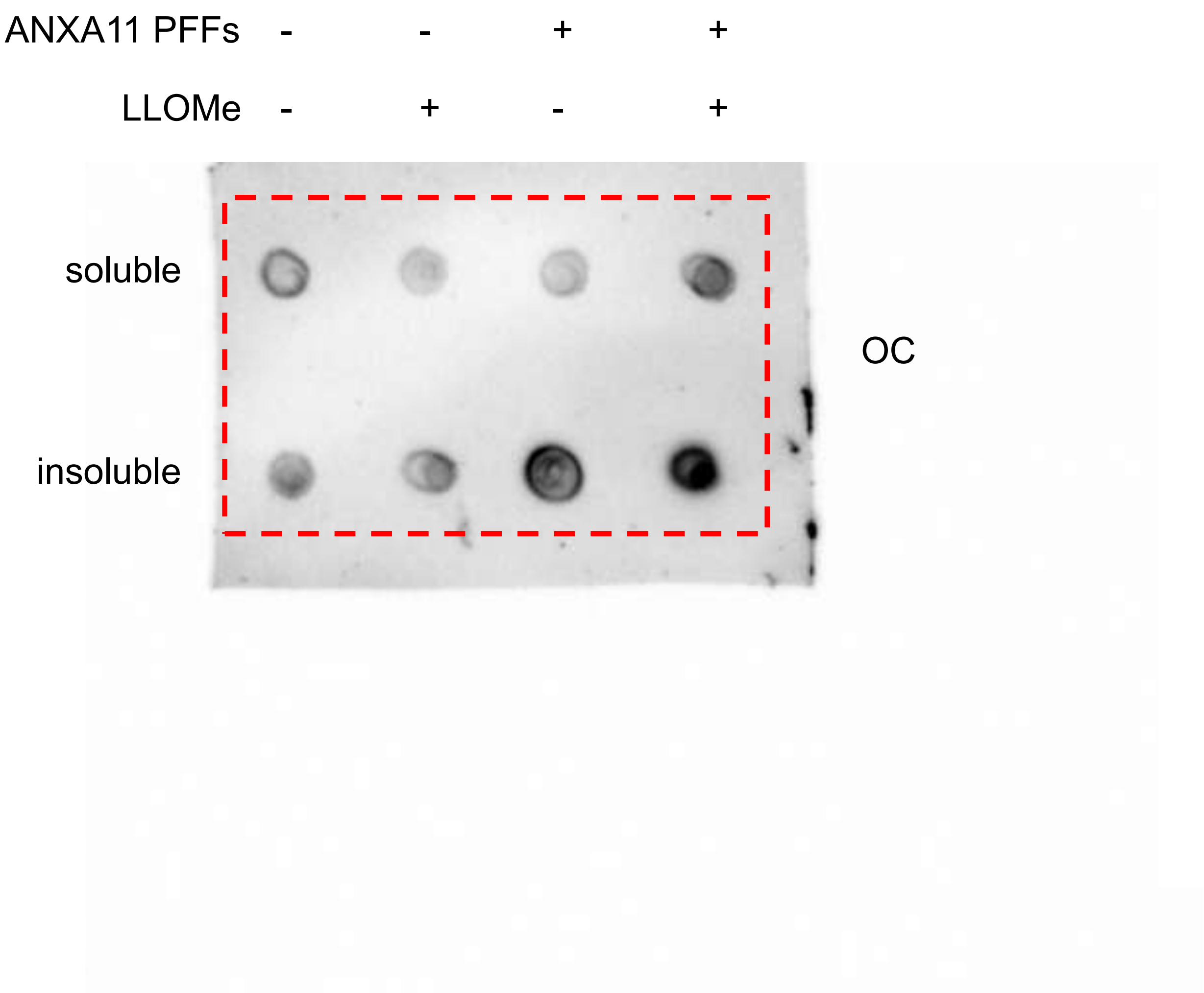

Figure 5d

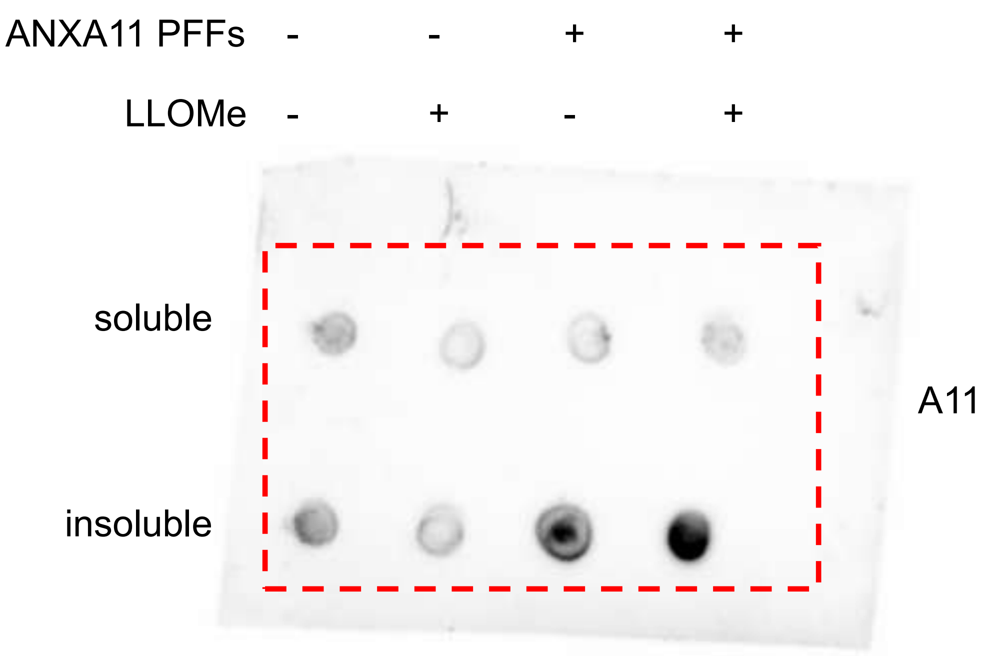

Figure 6h

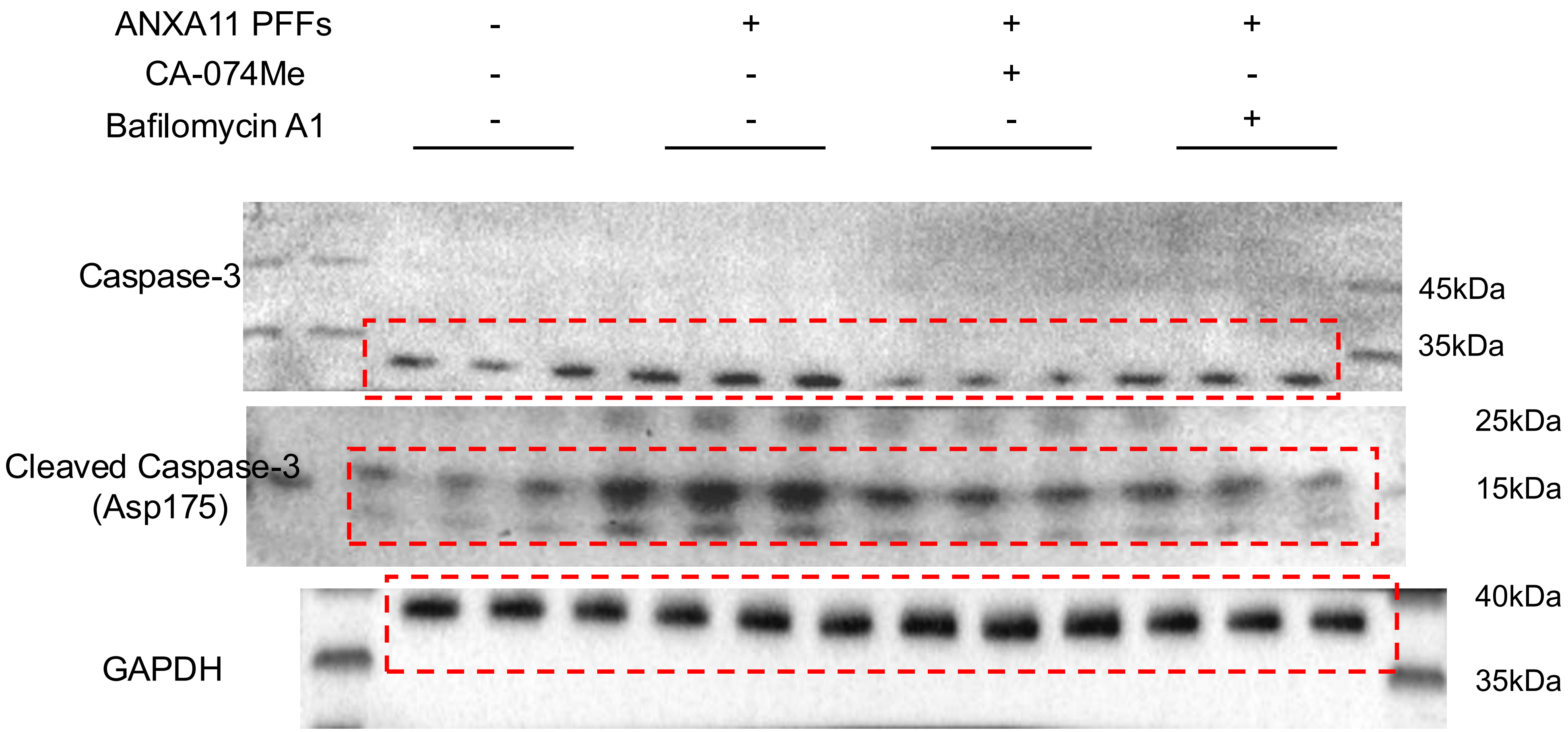

ANXA11 PFFs - + + +
CA-074Me - - + -
Bafilomycin A1 - - - +
Caspase-3
45kDa
35kDa
25kDa
Cleaved Caspase-3
(Asp175)
15kDa
40kDa
GAPDH
35kDa

Figure 7j

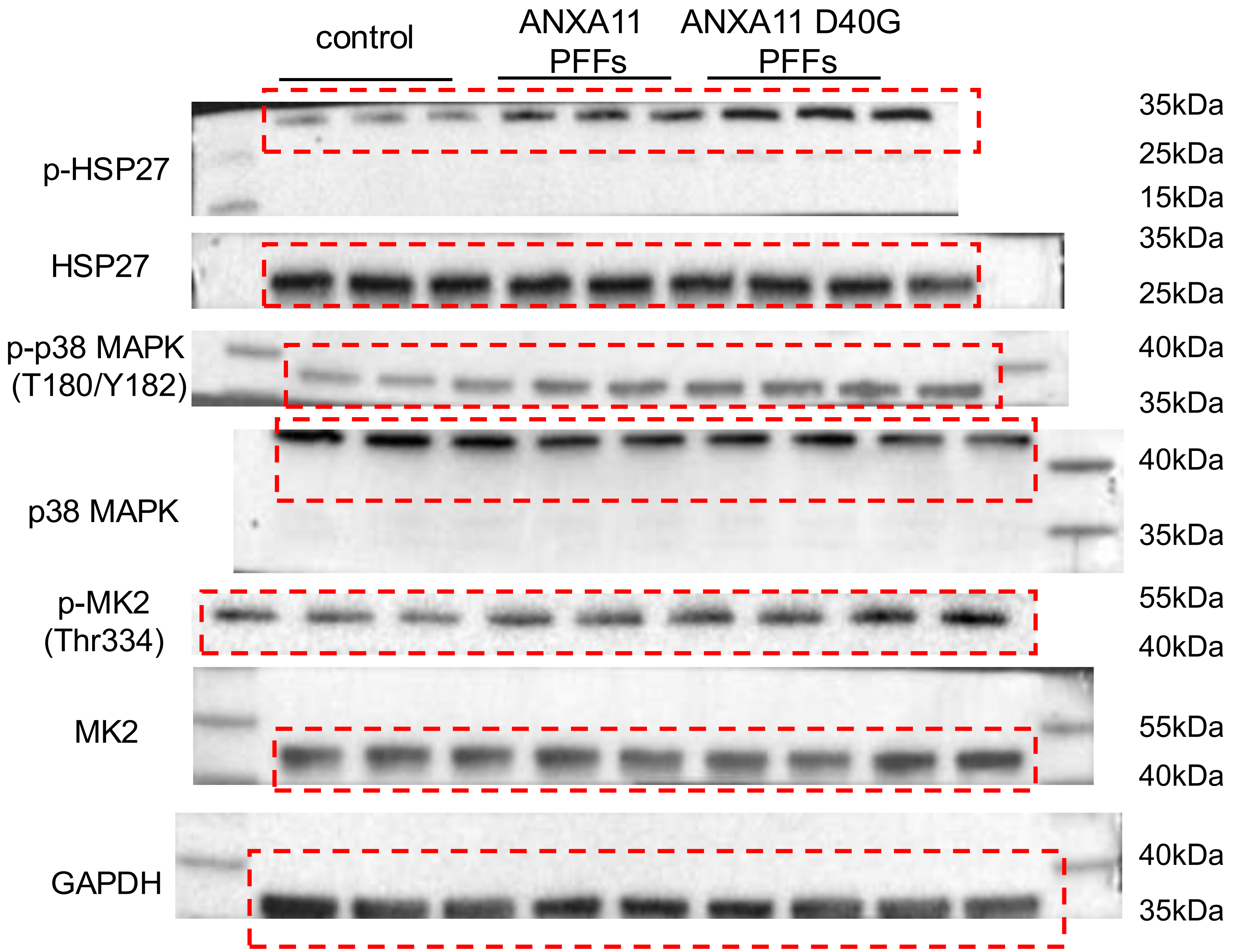
control
ANXA11 PFFs
ANXA11 D40G PFFs
p-HSP27
HSP27
p-p38 MAPK (T180/Y182)
p38 MAPK
p-MK2 (Thr334)
MK2
GAPDH
35kDa
25kDa
15kDa
35kDa
25kDa
40kDa
35kDa
40kDa
35kDa
55kDa
40kDa
55kDa
40kDa
40kDa
35kDa

Figure S1a

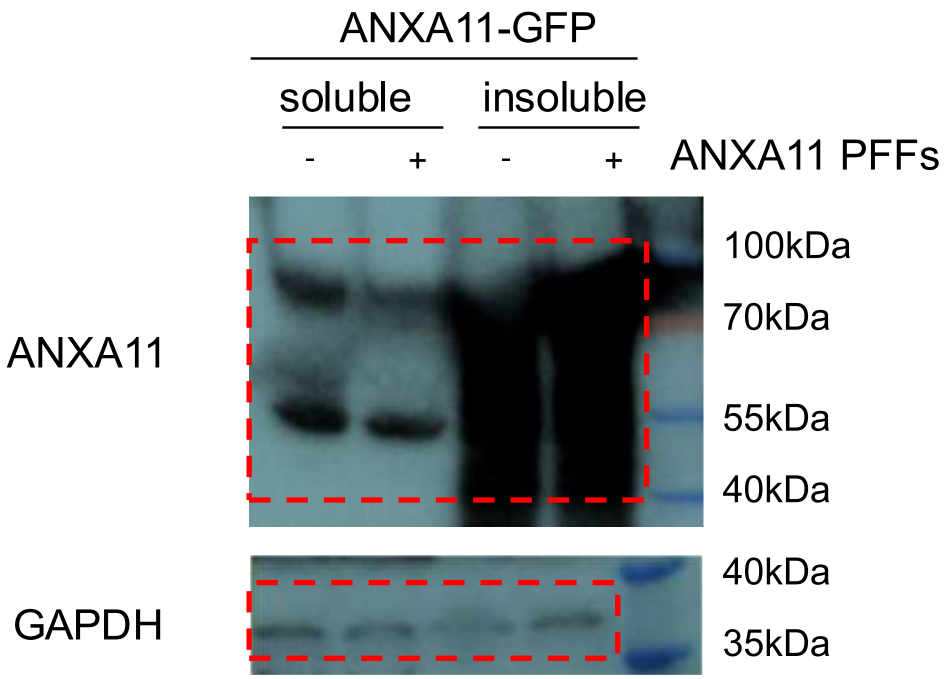
ANXA11-GFP
soluble
insoluble
- + - + ANXA11 PFFs
ANXA11
100kDa
70kDa
55kDa
40kDa
GAPDH
40kDa
35kDa

Figure S4a

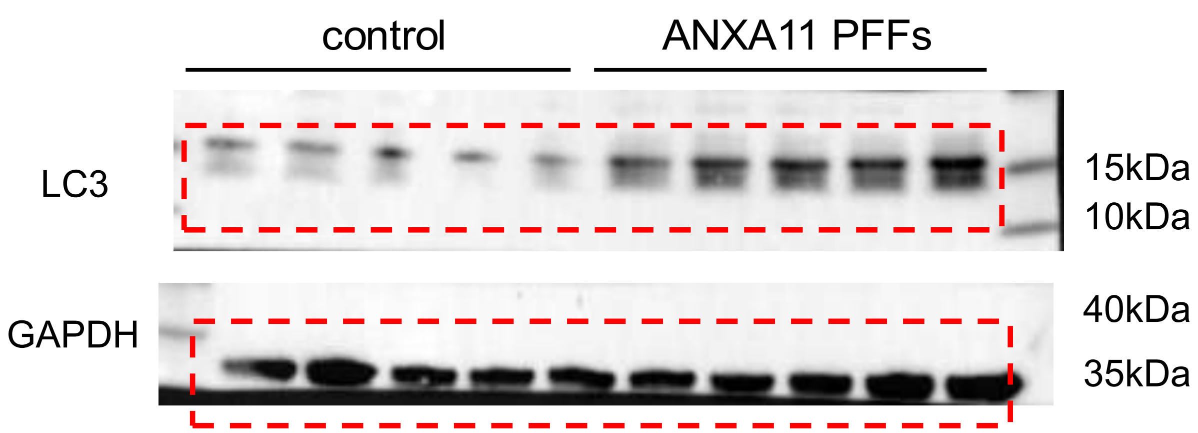

Figure S4d

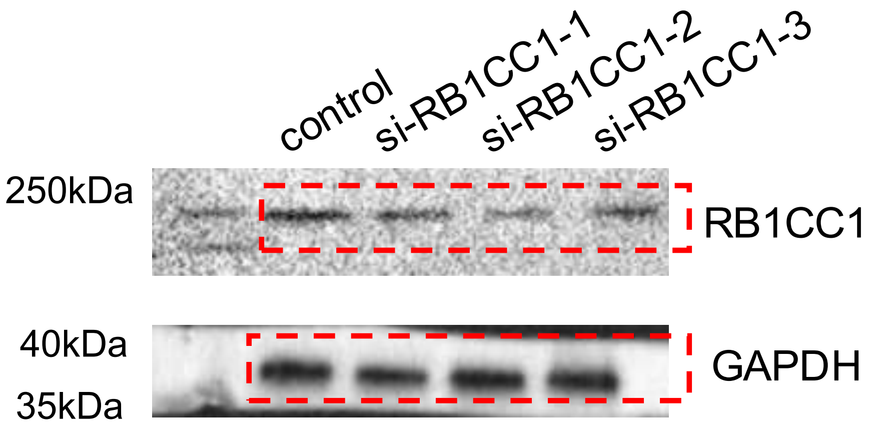

Figure S5g

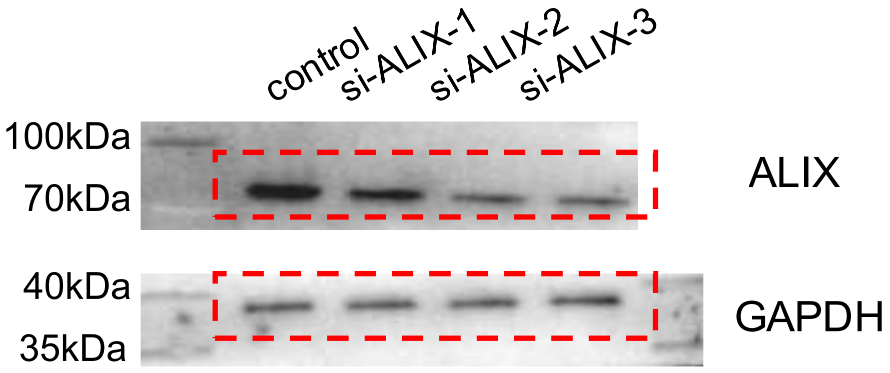

Figure S5i

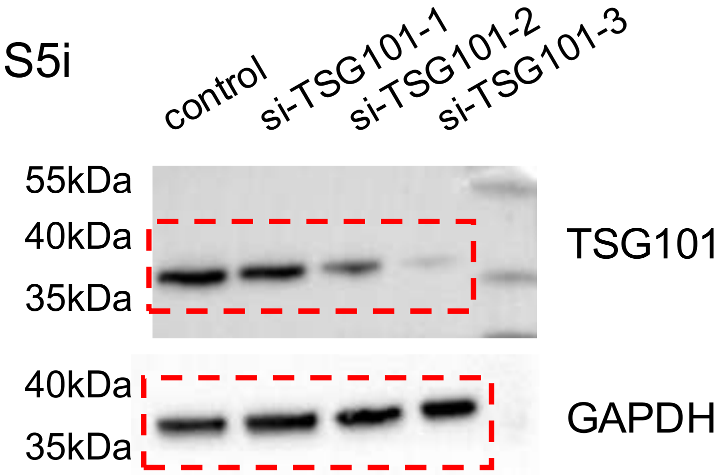

Figure 7a

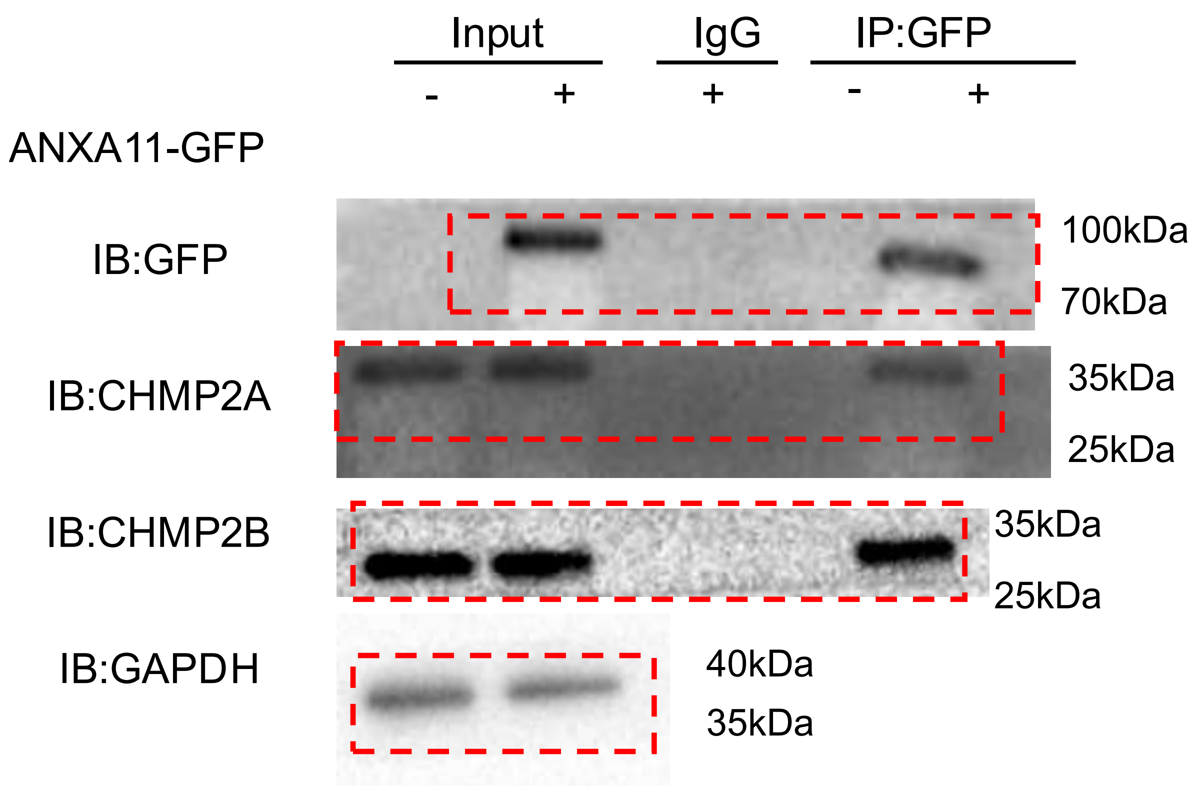

Figure 7c

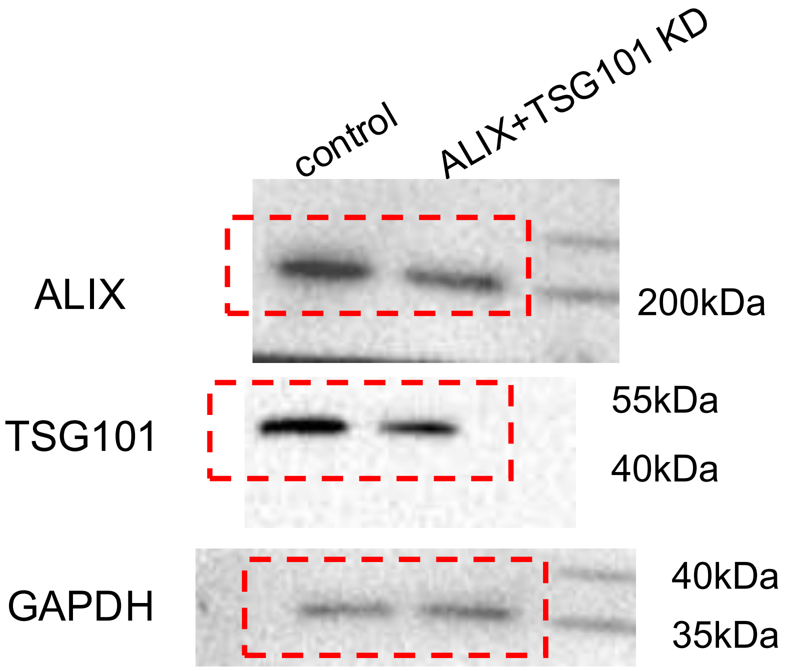

Figure S9i

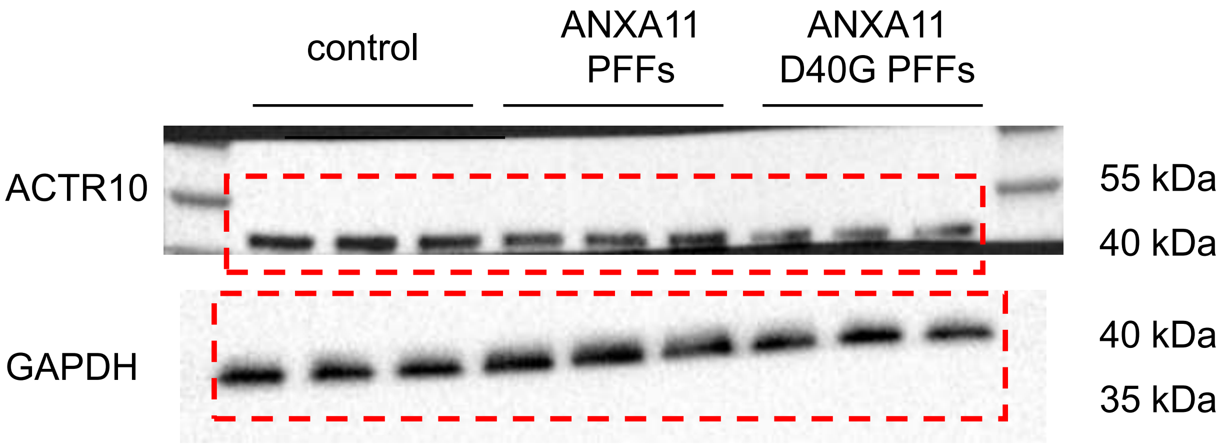
control
ANXA11 PFFs
ANXA11 D40G PFFs
ACTR10
55 kDa
40 kDa
GAPDH
40 kDa
35 kDa
